# Efficacy of probiotics for treatment of acute or persistent diarrhoea in children from birth till 10 years: Systematic review and meta-analysis

**DOI:** 10.7189/jogh.14.04236

**Published:** 2024-12-20

**Authors:** Anmol Minaz, Ridwa Alam, Uswa Jiwani, Khadija Vadsaria, Ahmad Khan, Aqsa Ishaq, Samar Sultan, Marium Mohsin, Ashraf Sharif, Yasir Bin Nisar, Jai K Das, Sajid Soofi, Shabina Ariff

**Affiliations:** 1 Centre of Excellence in Women and Child Health, Aga Khan University, Karachi, Pakistan; 2 University Library, Aga Khan University, Karachi, Pakistan; 3 Department of Maternal, Newborn, Child and Adolescent Health and Ageing, World Health Organization, Geneva, Switzerland; 4 Institute for Global Health and Development, Aga Khan University, Karachi, Pakistan; 5 Department of Paediatrics and Child Health, Aga Khan University, Karachi, Pakistan

## Abstract

**Background:**

Numerous studies have investigated the efficacy of probiotics in treating acute and persistent diarrhoea. However, probiotics have not been established as a recommended management option for diarrhoeal illness by the World Health Organization (WHO). Therefore, we conducted a systematic review of randomised controlled trials to assess the efficacy of probiotics for the management of acute and persistent diarrhoea in children.

**Methods:**

A systematic search on PubMed, CINAHL, Wiley Cochrane Library, Scopus, Clinicaltrials.gov, and WHO International Clinical Trials Registry Platform (ICTRP) was performed. All studies published in the year 2000 and onwards that assessed the use of probiotics in the management of acute and persistent diarrhoea in children aged 0–10 years were included. The risk of bias was assessed using the Cochrane Risk of Bias II (RoB-2) tool and the quality of evidence was assessed using the Grading of Recommendations, Assessment, Development, and Evaluations (GRADE) approach. This review was commissioned by WHO for revision of their guidelines for childhood diarrhoea management.

**Results:**

The review included 98 studies with a total of 17 236 participants. Studies were categorised based on the WHO definition of diarrhoea or author-specified definition. In studies considering the WHO definition of diarrhoea, the probiotics group was more likely to achieve clinical cure (risk ratio = 1.12 (95% confidence interval (CI) = 1.01, 1.24, studies = 14)) and reduce the duration of diarrhoea (mean difference = −13.27 hours (95% CI = −16.72, −9.83, studies = 33)) than the control group in children with acute diarrhoea. However, the effect size was small, and statistical heterogeneity was very high, leading to low certainty of evidence. In children with persistent diarrhoea, probiotics reduced the duration of diarrhoea by 95 hours (mean difference = −96.45 (95% CI = −110.53, −82.37, studies = 2)), but the certainty of the evidence was very low.

**Conclusions:**

The results from this systematic review suggest low certainty of evidence for the effect of probiotics on clinical cure and duration of diarrhoea in children. There was significant diversity in the genus, species, dosages, and duration of treatment in the trial and administration. High levels of heterogeneity reduced the certainty of evidence. Large-scale randomised clinical trials are needed to evaluate specific probiotic strains and doses. In addition, cost-effective analysis studies are needed to be explored in future research.

**Registration:**

The protocol for this review was registered with the International Prospective Register of Systematic Reviews (PROSPERO: CRD42023449200).

Diarrhoeal diseases are the second leading cause of morbidity and mortality in children under five, accounting for an estimated 370 000 deaths in 2019 worldwide [1]. Most childhood diarrhoeal cases and the associated morbidity and mortality occur in low- and middle-income countries (LMICs). Children in these nations experience, on average, three diarrhoeal episodes every year [1], causing the death of one in every eighth child [2,3].

The World Health Organization (WHO) defines diarrhoea as the passage of three or more loose or liquid stools in a day or more frequent passage than is normal for an individual [4]. Diarrhoea typically manifests as a symptom of digestive tract infection caused by bacterial, viral, and parasitic organisms. It is classified into different clinical types: acute diarrhoea lasting less than 14 days, characterised by either watery or bloody consistency, and persistent diarrhoea lasting for 14–28 days [4].

The effective management of diarrhoea in children is crucial to reduce the associated mortality, prevent long-term consequences, and ensure optimal growth and development [5,6]. The WHO recommends rehydration with oral rehydration solution (ORS), zinc supplements, and nutrient-rich food as a mainstay of diarrhoea management. Along with the recommended care, probiotics, defined as ‘live microorganisms that may confer health benefits when administered in adequate amounts’ [7], have emerged as a potential intervention for the treatment of acute watery or persistent diarrhoea in children [8].

Several mechanisms of action for the beneficial effects of probiotics have been described in the literature, including immunomodulation, gut microbiome modulation, and improvement in barrier function [9]. However, the studies describing these mechanisms have limitations, and there is insufficient evidence as to how those effects are modulated and translated to improved clinical outcomes [9].

Numerous studies have investigated the efficacy of probiotics in treating acute and persistent diarrhoea. Chen et al. found probiotics to accelerate improvement in the faecal consistency [10]. Huang et al. reported probiotics reduce the duration of hospital stay, improve treatment efficacy, and decrease diarrhoea duration when added to standard treatment in children with acute diarrhoea [11]. However, a systematic review on the efficacy of probiotics for acute gastroenteritis reported lower duration of diarrhoea and hospitalisation in children under 18 years, with limitations such as higher heterogeneity, non-exclusive use of probiotics (also included synbiotics), low certainty of evidence and findings limited to Japan [12]. Additionally, one study in the review assessing the effects of probiotics in acute infectious diarrhoea in both children and adults did not support the use of probiotics due to marked heterogeneity between studies and a high or unclear risk of bias. Further, the review excluded persistent diarrhoea cases and studies involving yogurt [13].

Given the varied and unconvincing evidence from the existing clinical trials and systematic reviews, probiotics have not been established as a recommended management option for diarrhoeal illness by WHO [14]. In addition, the optimal probiotic strains, dosages, and treatment durations remain undefined. WHO commissioned this review to update its existing guidelines on childhood diarrhoea management and to synthesise existing literature regarding the effectiveness of probiotics. Therefore, a comprehensive evaluation of the existing literature through a systematic review is necessary to provide a synthesis of the current evidence and to ascertain the potential benefits and limitations of probiotic interventions for acute watery or persistent diarrhoea in children aged 0 to 10 years.

## METHODS

The protocol for this review was registered with the International Prospective Register of Systematic Reviews (PROSPERO: CRD42023449200). The systematic review adhered to the 2020 Preferred Reporting Items for Systematic Reviews and Meta-Analyses (PRISMA) guidelines to identify, appraise, and synthesise relevant studies.

### Eligibility criteria

Detailed eligibility criteria for this review are shown in **Table 1**.

**Table 1:** Inclusion and exclusion criteria

Inclusion criteria
Population
*Children (0–10 y with acute watery (≤14 d) or persistent diarrhoea (>14 d and <1 mo))*
Setting
*Low-income countries*
*Middle-income countries*
*High-income countries*
Study design
*Randomised controlled trials (RCTs)*
Type of interventions: probiotics containing single or multiple organism strains (live or heat-killed)
*Studies that compared the effect of probiotics vs. no probiotics (standard management similar in both the groups) in managing acute watery or persistent diarrhoea, or*
*Studies that compared the effect of probiotics vs. placebo in managing acute watery or persistent diarrhoea, or*
*Studies that compared the different strains or doses of probiotics*
Control arm
*Placebo (as defined by author), or*
*Placebo (as defined by author), or*
*Probiotics (for probiotics vs probiotics comparison)*
**Exclusion criteria**
Studies published before the year 2000
Studies on prevention of diarrhoea, animal studies, studies without outcome of interest, and studies on diarrhoea due to other causes (nosocomial, functional, or iatrogenic causes)
Children with chronic or bloody diarrhoea (dysentery)
Studies including prebiotics, synbiotics, or different standard management than the intervention group

### Primary outcomes

The following are the primary outcomes included in our study.

– Clinical cure: studies with either the WHO definition or an author-specified definition of diarrhoea, were considered to define this outcome. According to the WHO definition of diarrhoea, clinical cure was defined as the passage of less than three stools in 24 hours. In studies with author-defined diarrhoea, the definition of clinical cure was based on stool consistency, frequency, or a combination of both.

– Clinical deterioration: clinical deterioration was defined as the need for intravenous (IV) fluid rehydration, hospitalisation, or the worsening or onset of new symptoms.

– Duration of diarrhoea: the duration of diarrhoea was measured in hours, based on either the WHO definition or an author-specified definition.

– Adverse Events (AEs) and Serious Adverse Events (SAEs): complications that occurred during treatment, as defined by the study authors.

– Mortality: the total number of deaths that occurred during the study period.

### Search methods for identification of studies

The search strategy was developed based on PICO using free-text and MeSH terms to retrieve eligible studies (Appendix S1 in the **Online Supplementary Document**). Electronic searches were conducted in databases, including PubMed, CINAHL, Wiley Cochrane Library, and Scopus. We attempted to identify all the relevant studies regardless of the language. We applied date restriction to the studies where studies published from 2000 onwards were included. We also searched clinical trial registries, including clinicaltrials.gov and the WHO International Clinical Trials Registry Platform (ICTRP), to ensure that all relevant studies were included. Further, the bibliographies of previous relevant systematic reviews and all included studies were also searched.

### Data collection and analysis

#### Selection of studies

All records identified by the search were imported to Covidence for screening (Covidence systematic review software VHI, Melbourne, Australia). After deleting the duplicates, two review authors independently reviewed the titles and abstracts of the studies. All studies meeting the eligibility criteria were considered for the full-text screening. The disagreements encountered at this stage were discussed among the two reviewers or resolved by contacting the third reviewer. A similar process was followed for the full-text screening. The reasons for excluding studies at the full-text screening stage were recorded.

#### Data extraction and management

Data extraction was independently completed by two review authors onto a standardised data extraction form in Microsoft Excel. The extraction form has also been piloted. The data extracted was compared and reviewed to identify inconsistencies or conflicts. In cases of inconsistencies, the study was reviewed again, and discussion was held to come to a consensus.

Furthermore, for the studies where the full-text articles were not available, the list was shared with the authors for its retrieval using other sources. In addition, the study authors were contacted and followed up requesting the full-text articles. Studies were marked as not retrieved after exhausting all possible options. A similar process was conducted for the conference abstracts, presentations, and studies identified through the registries.

The quality assessment of the included RCTs was conducted using the Cochrane Risk of Bias tool II (RoB-2) [15]. Two reviewers independently assessed the risk of bias for each outcome of each study using this tool. Differences in risk of bias assessment were resolved by discussion or involving a third reviewer. Each potential source of bias was graded as low, high, or some concern. The following domains were assessed in the RoB-2: randomisation process (D1), deviation from intended intervention (D2), missing outcome data (D3), measurement of the outcome (D4), and selection of the reported result (D5).

### Statistical analysis

The meta-analysis was conducted on Review Manager (RevMan) 5.4.1 software (The Cochrane Collaboration, Copenhagen, Denmark, 2020). For continuous outcomes, the mean difference and the corresponding 95% confidence interval (CI) were considered. For studies that do not report exact mean and standard deviation (SD), Hozo’s method was used to calculate mean and SD from median, range, interquartile range, or CI [16]. For studies with missing SDs, data was imputed from studies with similar context. For dichotomous outcomes, the risk ratio (RR) and the corresponding 95% CI were reported. For studies with more than two comparison groups, any irrelevant groups were excluded from the analysis, and relevant groups were merged if appropriate. For trials with more than two intervention and control arms, the study data was entered multiple times for analysis, and relevant comparisons were made between intervention and control arms.

Forest plots were created for all the outcomes. The results of trials that provided data unsuitable for inclusion in pooled analyses or outcomes reporting geometric mean were described in the textual content of this review.

#### Assessment of heterogeneity

Statistical heterogeneity was assessed by *I*^2^ statistics and by visually inspecting forest plots to detect non-overlapping CIs. Heterogeneity was considered significant if *I*^2^ was greater than 50% and *P* < 0.10 in the Chi-square (χ^2^) test for heterogeneity. Based on prior clinical knowledge, clinical and methodological heterogeneity in the included studies was expected. Therefore, we tried to explain any observed statistical heterogeneity using sub-group analysis.

#### Assessment of reporting biases

For outcomes including more than 10 trials, a funnel plot was created and examined to explore possible publication bias. A regression approach was used to assess funnel plot asymmetry.

### Subgroup analysis

We undertook pre-planned subgroup analysis according to different probiotic genus, strains, age groups, time points at which outcome was assessed (clinical cure), and funding source. For this, studies were further categorised based on WHO definition of diarrhoea or author-specified definition.

### Sensitivity analysis

A sensitivity analysis was conducted to examine studies that controlled potential confounders. For this review, we conducted a sensitivity analysis based on the risk of bias.

### Certainty of evidence (GRADE)

A summary of the finding tables was generated for all outcomes presenting the quality of evidence according to the Grading of Recommendations, Assessment, Development, and Evaluation (GRADE) criteria [17]. It covers consideration of within-study risk of bias, directness of evidence, heterogeneity, precision of effect estimates, and risk of publication bias. For GRADE, we only considered studies with a low risk of bias. The quality of the evidence was then rated as ‘high’, ‘moderate’, ‘low’, or ‘very low’ for each outcome.

## RESULTS

**Figure 1:**
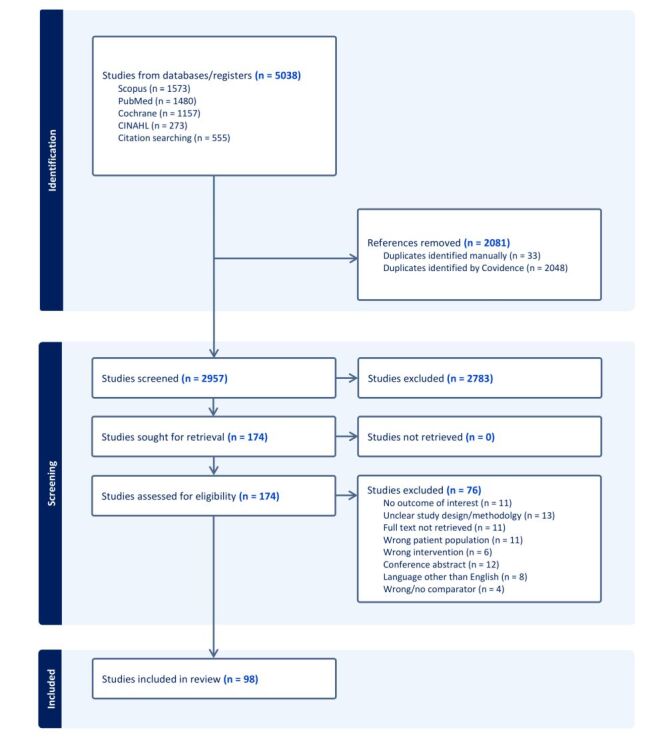
Study flow diagram (PRISMA).

The search of electronic databases, conducted on 27 July 2023, along with the search of trial registries and bibliographies, yielded 5038 citations as shown in **Figure 1****.** A total of 2957 records were left after removing all the duplicates. After screening titles and abstracts, we identified 174 citations for full-text review. Overall, 76 citations were excluded, including twelve that were conference abstracts and eleven studies for which full text was not retrieved.

### Study characteristics

A total of 98 studies were included in this review (Appendix S2 in the **Online Supplementary Document**). None of the included trials were cluster randomised. Two studies out of 98 studies looked at persistent diarrhoea [18,19]. A description of study characteristics is shown in **Table 2**. Of the total studies, two studies [20,21] had three arms and tested two different doses of probiotics with a control group, and one study [22] tested probiotics at two different temperatures with a control arm. One of the trials was a multi-country trial [23], and four studies [24–27] did not report on the country where the study was conducted.

**Table 2:** Characteristics of included studies (n = 98)

**Study characteristics**	**Number of studies (n)**
Age of study participants	
≤5 y*	71
*≤2 y*	18
*Other*	9
*Not specified*	6
Type of diarrhoea evaluated	
*Acute diarrhoea*	96
*Persistent diarrhoea*	2
Year of publication	
*2000–2010*	41
*2011–2020*	45
*After 2020*	12
Number of arms	
*Two arms*	80
*Three or more arms*	18
Study location	
*Asia*	60
*Africa*	5
*Europe*	6
*Asia/Europe (Turkey)*	8
*North America*	4
*South America*	9
*Australia*	1
*Multi-country trial*	1
*Not reported*	4
Income region classification of study country	
*Lower-income region*	-
*Lower-middle income region*	49
*Upper-middle income region*	26
*High-income region*	18
*Not specified*	4
Study setting	
*Inpatient*	60
*Outpatient*	16
*Other (Both, Emergency Department, community setting, etc.)*	22
Total study participants, n (range)	
*17 236 (27–971)*	

*Inclusive of age group ≤2 y.

#### Participant characteristics

The 98 studies included and randomised a total of 17 236 participants with a range of 27–971 individuals. A total of 71 studies included participants with age less than or equal to five years, with 18 studies included participants aged ≤2 years. Nine studies also included participants who were more than five years old. Six studies did not mention age range in the inclusion criteria.

Fifty-five studies excluded participants with previous antibiotic use, and two studies recruited participants irrespective of antibiotic use. Moreover, two trials excluded participants who were administered zinc. A total of 44 studies mentioned malnutrition in their exclusion criteria.

#### Interventions

A number of different probiotics were tested. Three studies [28–30] compared probiotics with other probiotics and included no control group. Out of the total, 31 studies tested multi-strain probiotics (combination of different organisms) in their intervention arms, 62 studies tested single-strain probiotics and five studies tested both single and multi-strain probiotics. The most common probiotic strain among all the studies was *Saccharomyces boulardii* followed by strains from genus Lactobacillus (*Lactobacillus rhamnosus*, *Lactobacillus reuteri*, *Lactobacillus acidophillus*). Some studies also evaluated *Bacillus clausii*, *Bifidobacterium lactis*, *Bifidobacterium bifidum* and *Escherichia coli*.

A total of sixty studies used a placebo, and eight studies used yogurt in the control group [31–38].

#### Risk of bias

Risk of bias assessment was completed according to outcomes reported in the meta-analyses. For WHO-defined clinical cure, eight studies had a high risk of bias; four had some concerns, and two had a low risk of bias (Figure S1 in the **Online Supplementary Document**). Studies were graded on multiple domains of ROB. Of the eight studies with a high risk of bias, four were considered high risk for deviation from the intended intervention, three for missing outcome data, and five studies on measurement of the outcomes.

For studies considering WHO-defined diarrhoea and reported clinical deterioration, two studies had a high bias risk, four had some concerns, and five had a low bias risk (Figure S2 in the **Online Supplementary Document**). For the two studies classified as high risk, one was marked high risk for missing outcome data and the other for measurement of the outcome.

The risk of bias for the duration of diarrhoea (WHO-defined diarrhoea) was high in 14 studies, with some concerns in 14 studies, and low risk in five studies (Figure S3 in the **Online Supplementary Document**). Among the studies with a high risk of bias, one was considered high risk on the domain of randomisation process, four studies for deviation from the intended intervention domain, five studies on missing outcome data, 11 studies on the measurement of the outcome, and one on the selection of the reported result.

Out of the 42 studies that reported AEs, 15 studies had a high risk of bias, 23 had some concerns, and four were low risk of bias studies (Figure S4 in the **Online Supplementary Document**). In the high-risk-of-bias studies, two were considered in the domain of randomisation process, one in deviation from the intended intervention domain, five in missing outcome data, 11 in the measurement of the outcome domain, and one in the selection of the reported result domain.

Moreover, of the 15 studies that reported SAEs, two had high risks of bias, eight were marked as some concerns, and five were low risk (Figure S5 in the **Online Supplementary Document**). One study was marked as high risk in the missing outcome data domain, whereas two studies were high risk for the measurement of the outcome. For the outcome of mortality, all studies were considered low risk of bias.

For two studies on persistent diarrhoea, both were considered as some concerns for risk of bias for the duration of diarrhoea outcome.

### Outcome results (for acute diarrhoea)

#### Clinical cure

A total of 44 studies reported clinical cure. Studies were categorised based on the WHO definition of diarrhoea or author-specified definition. Fourteen studies defined clinical cure according to the WHO criteria of diarrhoea. Among the studies with author-defined diarrhoea, 11 based their definition of clinical cure on consistency, three on frequency, and four had unclear features attributed to the resolution of diarrhoea. Additionally, 12 studies reported clinical cure based on both consistency and frequency of diarrhoea.

The overall results demonstrated that the probiotic group was 21% more likely to achieve clinical cure compared to the control group RR = 1.21 (95% CI = 1.12, 1.30; studies = 44, total participants = 6318) (Figure S6 in the **Online Supplementary Document**). However, the results showed very high heterogeneity (*I*^2^ = 97%). The summary of findings for all outcomes is shown in **Table 3**. In studies with the WHO-defined clinical cure, similar results were drawn RR = 1.12 (95% CI = 1.01, 1.24; studies = 14, total participants = 2618), suggesting a marginally significant difference between the probiotic and control group (Figure S7 in the **Online Supplementary Document**), with significant heterogeneity (*I*^2^ = 97%). Sensitivity analysis for low risk of bias studies included five studies (total participants = 1207), of which three studies showed results favouring probiotics (Figure S8 in the **Online Supplementary Document**).

**Table 3:** Summary of results for outcomes of acute diarrhoea

Outcome	Number of studies, n	Point estimate (95% CI)	Effect	Heterogeneity	Evidence certainty
Clinical cure (overall)	44	1.21 (1.12, 1.30)	Probiotics better	97%	-
Clinical cure *(WHO-define*d)	14	1.12 (1.01, 1.24)	Probiotics better	97%	-
Clinical cure (sensitivity analysis-ROB)	5	1.23 (1.01, 1.49)	Probiotics better	96%	Low
Clinical deterioration (overall)	18	1.04 (0.82, 1.33)	No difference	4%	-
Clinical deterioration (WHO-defined)	11	1.12 (0.81, 1.55)	No difference	12%	-
Clinical deterioration (sensitivity analysis-ROB)	6	1.16 (0.83, 1.60)	No difference	0%	Low
Duration of diarrhoea (overall)	67	−15.56 (−18.30, −12.82)	Probiotics better	95%	-
Duration of diarrhoea (WHO-defined)	33	−13.27 (−16.72, −9.83)	Probiotics better	92%	-
Duration of diarrhoea (sensitivity analysis-ROB)	11	−7.20 (−13.36, −1.03)	Probiotics better	81%	Low
Adverse events	42	0.94 (0.81, 1.08)	No difference	0%	-
Adverse events (sensitivity analysis-ROB)	11	0.91 (0.79, 1.06)	No difference	0%	Low
Serious adverse events	15	0.69 (0.26, 1.83)	No difference	15%	-
Serious adverse events (sensitivity analysis-ROB)	6	0.81 (0.23, 2.81)	No difference	32%	Low
Mortality*	4	0.17 (0.03, 0.98)	No difference	0%	Low

CI – confidence interval, ROB – risk of bias, WHO – World Health Organization

*The cause of mortality was not explicitly mentioned in the studies.

Twelve studies assessed clinical cure at four different time points, including day three, five, seven, and 14 after the initiation of probiotics. On day seven, none of the studies showed a statistically significant effect of the probiotic RR = 1.35 (95% CI = 1.14, 1.60; studies = 3, total participants = 320). For the remaining time points (days three, five, and 14), the results were comparable in both groups (Figure S9 in the **Online Supplementary Document**).

#### Clinical deterioration

Overall, 18 studies reported clinical deterioration. In total, 151 / 2613 participants in the probiotic group and 128 / 2237 participants in the control group reported to be clinically deteriorated. The results did not show a statistically significant difference between the intervention and control arms RR = 1.04 (95% CI = 0.82, 1.33; studies = 18, total participants = 4850) (Figure S10 in the **Online Supplementary Document**), and the heterogeneity of the findings was very low (*I*^2^ = 4%).

Using a similar approach of categorising studies based on the WHO definition of diarrhoea, we found 11 studies (total participants = 3603) that reported clinical deterioration. In the probiotic group, clinical deterioration was reported in 107 / 1989 participants, whereas in the control group, it was reported in 80 / 1614 participants. However, the findings were not statistically significant (Figure S11 in the **Online Supplementary Document**). Sensitivity analysis for low risk of bias studies included six studies (total participants = 2626) showing comparable results (Figure S12 in the **Online Supplementary Document**).

#### Duration of diarrhoea

Overall, 71 studies reported the duration of diarrhoea, and among these, data from four studies was not included in the meta-analysis due to un-extractable data [22,39–41]. The results showed a reduction in the duration of diarrhoea by approximately 16 hours in the probiotic group compared to the control group (studies = 67, total participants = 11 830) (Figure S13 in the **Online Supplementary Document**). However, the heterogeneity was very high (*I*^2^ = 95%), showing low confidence in our findings.

Of 71 studies, 33 (total participants = 7598) reported diarrhoea using WHO’s definition. These studies showed a reduction of diarrhoea by 13 hours in the probiotic group compared to the control group (Figure S14 in the **Online Supplementary Document**). The heterogeneity was still high (*I*^2^ = 92%). On sensitivity analysis, low-risk studies showed a decrease in diarrhoea duration by seven hours in the intervention group compared to the control group (studies = 11, total participants = 3660). The level of heterogeneity remained substantial (Figure S15 in the **Online Supplementary Document**).

#### Adverse events

A total of 53 studies reported AEs or SAEs. Among 42 studies where AEs were reported, 25 did not provide specific definitions or descriptions of these events, and 17 reported some form of description. AEs were reported as zero in 31 studies. Overall, 274 / 4111 AEs were reported in the probiotic group compared to 284 / 3787 events in the control group. Despite fewer events in the probiotic group, this difference did not demonstrate a statistically significant result compared to the control group RR = 0.94 (95% CI = 0.81, 1.08) (Figure S16 in the **Online Supplementary Document**). The sensitivity analysis also showed comparable results (Figure S17 in the **Online Supplementary Document**).

One study reported AEs that were coded using definitions from the Medical Dictionary for Regulatory Activities, version 19.0 [42]. Three studies [43–45] reported fever in both groups. Lahiri et al. [43] reported severe dehydration in both probiotic and control groups, whereas Salazar-Lindo et al. [46] observed it only in the probiotic group. Two studies [27,44] reported vomiting as an AE in the control group, while Lahiri et al. [43] reported it for the probiotic group. Abdominal pain was noted in the probiotic group in two studies [26,27]. Additionally, one study [43] reported nasopharyngitis in both probiotic and control groups, whereas another study [27] reported it for control groups only.

Moreover, Mourey et al. [44] also considered AEs as an increase in diarrhoea symptoms requiring IV fluid therapy/rescue medicine (observed in both groups). Additionally, one study [45] reported diarrhoea as one of the AEs. Other AEs observed in this study were dehydration, pyrexia, ear infection, and wheezing. All events were observed in both the probiotic and control groups, except for wheezing. Billoo et al. [47] reported side effects in both groups. Two studies [20,48] reported complications in the form of electrolyte imbalance, septicaemia, and renal failure in both groups. Additionally, one study [49] discussed that probiotics administration was not associated with AEs, but did not provide any numbers or descriptions of AEs.

Other symptoms reported in the probiotic group included rhinitis [26], constipation [50], limb injury, traumatic hematoma, and hypersensitivity [27]. In the control group only, AEs reported were rash [46], severe weakness [44], acute otitis media [26], influenza, cough, dermatitis, and rhinorrhoea [27].

#### Serious adverse events

In total, 15 studies reported SAEs, and out of these, 10 studies did not describe SAEs. A total of 12 SAEs were reported in the probiotic group (n = 1733) compared to 18 in the control group (n = 1747). These results did not show any statistical significance (Figure S18 in the **Online Supplementary Document**). The sensitivity analysis based on the risk of bias also did not demonstrate any significant findings (Figure S19 in the **Online Supplementary Document**). Sindhu et al. [51] mentioned SAEs as hospitalisation due to respiratory infections, vulval abscess, and measles. Another study [45] reported dehydration, worsening diarrhoea, diarrhoea haemorrhagic, febrile convulsion, ileus paralytic, mental status changes, metabolic acidosis, and seizure as SAEs in the probiotic group. In the control group, bronchiolitis, vomiting, impaired gastric emptying, and dehydration, worsening gastroenteritis, pneumonia, lethargy, lower respiratory tract infection, and pyrexia were reported. Additionally, Freedman et al. [42] reported no SAEs in the intervention group compared to the placebo group, while Ghosh et al. [52] reported two SAEs in the form of severe dehydration requiring hospitalisation in the placebo group.

#### Mortality

In total, five studies reported mortality [20,33,43,53,54]. Four studies did not mention if mortality was related to diarrhoea, whereas one study [33] reported one death due to secondary hemophagocytic syndrome. For our meta-analysis, we reported the former four studies. Overall, one death was reported in the probiotic group (n = 781) and seven deaths in the control group (n = 594) (Figure S20 in the **Online Supplementary Document**).

One study [20] reported no death in the probiotic group and one death in the placebo group, whereas another study [43] reported no deaths in either group. Pernica et al. [53] was a pilot study leading up to a multicentre trial [54], both analysing 7-day and 60-day mortality as secondary outcomes. While the pilot study [53] reported no deaths observed in either the probiotic or control groups, the trial [54] reported one death (1 / 135) in the probiotic group and six deaths (6 / 137) in the control group.

### Outcome results (for persistent diarrhoea)

Two studies included children with persistent diarrhoea [18,19] (**Table 4**). Both studies defined persistent diarrhoea as diarrhoea lasting 14 days or more. Moreover, both the studies were double-blinded. Overall, the mean difference for the duration of diarrhoea was −96.45 hours (95% CI = −110.53, −82.37; n = 324) between the probiotic group and the control group showing a significant effect of probiotics. The heterogeneity was found to be 0% (Figure S21 in the **Online Supplementary Document**). The mean difference for the duration of diarrhoea was relatively higher (mean difference = −114 hours (95% CI = −151.65, −76.35)) in the study by Gaón et al. [19] compared to the mean difference of −93.6 hours (95% CI = −108.78, −78.42) reported by Basu et al. [18].

**Table 4:** Summary of results for outcomes of persistent diarrhoea

Outcome	Number of studies (n)	Point estimate (95% CI)	Effect	Heterogeneity	Evidence certainty
Duration of diarrhoea	2	−96.45 (−110.53, −82.37)	Probiotics better	0%	Very low

CI – confidence interval

For clinical cure outcome, data from one trial suggested a greater likelihood of achieving clinical cure in the probiotic group compared to the control group RR = 8.38 (95% CI = 2.86, 24.57). For clinical deterioration outcome, Gaón et al. [19] reported no participants had worsening of symptoms (increased frequency of faecal motion, recurrence of dehydration, or GI symptoms) for the probiotic or control group.

AEs were recorded in Gaón et al. [19], but numbers were not mentioned by the author of the study. Basu et al. [18] reported complications like septicaemia (in both the probiotic and control groups) and renal failure (in the probiotic group) during the study period.

### Subgroup analysis

The subgroup analysis did not show any statistically significant results except for the duration of diarrhoea and mortality (Figures S22–37 in the **Online Supplementary Document**). The duration of diarrhoea was seen to be different when studies with single-strain probiotics were compared to studies with multiple strains. However, the heterogeneity within the subgroup remained high, showing low confidence in the findings (Figure S24 in the **Online Supplementary Document**). The results for mortality outcome when comparing single-strain probiotics with multi-strain probiotics also showed some differences, but the confidence intervals were wide (Figure S25 in the **Online Supplementary Document**). Similar results were also observed for subgroup analysis comparing different probiotic genus for duration of diarrhoea and mortality (Figures S28–29 in the **Online Supplementary Document**). Hence, there was significant diversity observed in the genus, species, dosages administered and duration of treatment across the studies.

### Certainity of evidence (GRADE)

A summary of findings for probiotic compared to control for treating acute watery and persistent diarrhoea is shown in **Table 5** and **Table 6**. For this grading, analysis was limited only to studies at low risk of bias.

**Table 5:** Summary of GRADE findings for acute diarrhoea outcomes

Certainty assessment							No. of patients		Effect		Certainty	Importance
**No. of studies**	**Study design**	**Risk of bias**	**Inconsistency**	**Indirectness**	**Imprecision**	**Other considerations**	**Probiotic**	**Placebo**	**Relative (95% CI)**	**Absolute (95% CI)**		
Clinical Cure (<3 stools in 24 h) – acute watery diarrhoea												
5	Randomised trials	Not serious	Serious*	Not serious	Serious†	None	555/604 (91.9%)	495/603 (82.1%)	RR 1.23 (1.01, 1.49)	189 more per 1000 (from 8 more to 402 more)	Low	Critical
Mortality – acute watery diarrhoea												
4	Randomised trials	Not serious	Not serious	Not serious	Very serious†	None	1/781 (0.1%)	7/594 (1.2%)	RR 0.17 (0.03, 0.98)	10 fewer per 1000 (from 11 fewer to 0 fewer)	Low	Critical
Duration of diarrhoea (hours) – acute watery diarrhoea												
11	Randomised trials	Not serious	Serious‡	Not serious	Serious§	None	1827	1833	-	x̄ 7.2 h lower (13.36 lower to 1.03 lower)	Low	Critical
Clinical deterioration (including IV fluid use, hospitalisations, worsening of symptoms) – acute watery diarrhoea												
6	Randomised trials	Not serious	Not serious	Not serious	Very serious†	None	72/1309 (5.5%)	63/1317 (4.8%)	RR 1.16 (0.83, 1.60)	8 more per 1000 (from 8 fewer to 29 more)	Low	Important
Serious adverse events – acute watery diarrhoea												
6	Randomised trials	Not serious	Not serious	Not serious	Very serious‖	None	12/1266 (0.9%)	16/1274 (1.3%)	RR 0.81 (0.23, 2.81)	2 fewer per 1000 (from 10 fewer to 23 more)	Low	Critical
Adverse events – acute watery diarrhoea (assessed with: vomiting, abdominal pain, rash)												
11	Randomised trials	Not serious	Not serious	Not serious	Very serious†	None	246/2265 (10.9%)	262/2053 (12.8%)	RR 0.91 (0.79, 1.06)	11 fewer per 1000 (from 27 fewer to 8 more)	Low	Important

CI – confidence interval, RR – risk ratio, x̄ – mean difference

*I^2^ = 96%.

**†**CIs include the null effect.

**‡***I*^2^ = 81%

§If 12 h is considered clinically important, the 95% CI crosses a decision-making threshold.

**‖**CI includes the null value.

**Table 6:** Summary of GRADE findings for persistent diarrhoea outcomes

Certainty assessment							No. of patients		Effect		Certainty	Importance
**No. of studies**	**Study design**	**Risk of bias**	**Inconsistency**	Indirectness	**Imprecision**	**Other considerations**	**Probiotic**	**Placebo**	**Relative (95% CI)**	**Absolute (95% CI)**		
Duration of diarrhoea (hours) – persistent diarrhoea												
2	Randomised trials	Serious*	Not serious	Serious†	Serious‡	None	177	147	-	x̄ = 96.45 h lower (110.53 lower to 82.37 lower	Very low	Critical

CI – confidence interval, x̄ – mean difference

*Both studies show overall some concerns of bias.

†Different definitions of the outcome are used.

**‡**Small sample size not meeting optimal information size.

## DISCUSSION

The findings from this systematic review indicate that there is low certainty of evidence that probiotics are effective in achieving clinical cure or reducing the duration of diarrhoea. Similarly, with low certainty of evidence, we found probiotics had no effect in preventing clinical deterioration in children with acute diarrhoea. Probiotics were not associated with adverse events or serious adverse events, but the number of studies reporting these were limited.

WHO does not currently recommend the use of probiotics in acute watery or persistent diarrhoea in children [14]. On the other hand, the European Society for Paediatric Gastroenterology, Hepatology and Nutrition (ESPGHAN) recommends the use of different probiotic strains like L. *rhamnosus* GG, S. *boulardii*, and L. *reuteri* in children with acute diarrhoea. However, their recommendation is based on evidence with a low to very low certainty, and their grade of recommendation is considered weak [55].

Building on these contrasting viewpoints, this review aligns with previous systematic reviews that have largely remained inconclusive about the efficacy of probiotics [13,56]. In contrast to our findings from strain-specific sub-group analysis, a review by Szajewska et al. [57] showed L. *rhamnosus* GG to reduce the duration of diarrhoea by approximately 20 hours. However, high heterogeneity and methodological limitations of the included trials were also mentioned in the review. Another review on S. *boulardii* was in line with our findings, noting significant effect of S. *boulardii* in reducing the duration of diarrhoea, and the quality of evidence was marked as very low [58].

A significant contributing factor to this inconclusiveness is the marked statistical heterogeneity observed across studies. The heterogeneity was similar even when sub-group analyses were performed according to single/multi-strain probiotics, probiotics genus, probiotics strains, funding information, risk of bias, or definition of diarrhoea used in the studies. One potential explanation for this heterogeneity is the variability in the outcome definition and the inclusion criteria used. Further, differences in the viability of the probiotics or the quality control procedures, including the temperatures at which they were stored, may also have influenced the trial outcomes. Additionally, the timing of probiotic initiation is a crucial factor; probiotics administered within 48 hours of diarrhoea onset might have different effects compared to those initiated after 48 hours of diarrhoea duration.

As discussed, one of the important limitations of this review is the heterogeneity in the findings leading to difficulty in drawing definitive conclusions. Moreover, the absence of study protocols for many included studies can be considered a challenge, making it difficult to assess the risk of bias. Additionally, we identified publication bias through funnel plots, indicating another limitation of this review. Despite our review indicating no association between probiotics and adverse events or serious adverse events, the lack of details about what constitutes adverse events or serious adverse events is considered a deficiency in reporting. Therefore, better reporting in terms of adverse events or serious adverse events is needed to evaluate the safety of probiotics.

## CONCLUSIONS

The findings from this systematic review will not only contribute to the existing body of knowledge but also inform health care providers, policymakers, and parents/guardians about the potential benefits of probiotics as an adjunctive therapy for acute watery or persistent diarrhoea in children. Furthermore, any identified gaps in the literature will provide valuable insights for future research and guide the development of evidence-based guidelines on the use of probiotics in this paediatric population. However, the results from this systematic review should be interpreted with great caution as the certainty of evidence for clinical cure and duration of diarrhoea was found to be low. Therefore, large-scale, multi-country clinical trials looking at specific probiotic strains and doses are needed. These trials should be designed with standardisation of outcome definitions, inclusion criteria, viability of probiotics, and other interventions given to trial participants. Also, there is a need for more clinical trials to explore the role of probiotics in persistent diarrhoea. Additionally, it is crucial to explore cost-effectiveness analyses concerning the use of probiotics in cases of acute diarrhoea.

## Additional material


Online Supplementary Document
