# Supplementary material for: Efficacy of probiotics for treatment of acute or persistent diarrhoea in children from birth till 10 years: Systematic review and meta-analysis
Source: J Glob Health. 2024 Dec 20;14:04236. doi: 10.7189/jogh.14.04236 (PMC11659791; doi:10.7189/jogh.14.04236)
Supplement: Online Supplementary Document [file jogh-14-04236-s001.pdf]

Efficacy of Probiotics for Treatment of Acute or Persistent Diarrhea in Children from Birth till Ten Years: Systematic Review and Meta-Analysis

Online Supplementary Document

Appendix S1

Search Strategy for the systematic review

PubMed

(Child[MeSH] OR Pediatrics[MeSH] OR Infant, Newborn[MeSH] OR Schools, Nursery[MeSH] OR child* OR neonatal OR infancy OR infant Or infantile OR baby OR babies OR toddler OR kid OR juvenile OR pediatric OR pediatric* OR paediatric* OR preterm OR premature OR birthweight OR "school age" OR "nursery school" OR preschool) AND (Diarrhea[MeSH] OR diarrh* OR Rotavirus[MeSH] OR rotavirus OR Dehydration[MeSH] OR dehydrat* OR Gastroenteritis[MeSH] OR gastroenteritis) AND (Probiotics[MeSH] OR probiotic OR lactobacill* OR lactococc* OR bifidobacter* OR enterococc* OR streptococc* OR saccharomyces OR escherichia OR bacill*) AND ("Randomized Controlled Trial" [Publication Type] OR "Randomized Controlled Trials as Topic"[Mesh] OR random* OR "RCT" OR trial)

Filters: Human, English

Cochrane

ID Search Hits

#1 MeSH descriptor: [Infant, Newborn] explode all trees 20567

#2 MeSH descriptor: [Child] explode all trees 78113

#3 MeSH descriptor: [Pediatrics] explode all trees 1179

#4 MeSH descriptor: [Schools, Nursery] explode all trees 43

#5 (child* OR neonatal OR infant OR infancy OR infantile OR baby OR toddler OR kid OR juvenile OR pediatric OR pediatric* OR paediatric* OR preterm OR premature OR birthweight OR "birth weight" OR "school age" OR "nursery school" OR preschool):ti,ab,kw (Word variations have been searched) 260927

#6 MeSH descriptor: [Diarrhea] explode all trees 7244

#7 MeSH descriptor: [Rotavirus] explode all trees 291

#8 MeSH descriptor: [Dehydration] explode all trees 855

#9 MeSH descriptor: [Gastroenteritis] explode all trees 10595

#10 (diarrh* OR rotavirus OR dehydrat* OR gastroenteritis):ti,ab,kw (Word variations have been searched) 37078

#11 MeSH descriptor: [Probiotics] explode all trees 3058

#12 (probiotic* OR lactobacill* OR lactococc* OR bifidobacter* OR enterococc* OR streptococc* OR saccharomyces OR escherichia OR bacill*):ti,ab,kw (Word variations have been searched) 23870

#13 MeSH descriptor: [Randomized Controlled Trial] explode all trees 25732

#14 MeSH descriptor: [Randomized Controlled Trials as Topic] explode all trees 47396

#15 (random* OR RCT OR trial):ti,ab,kw (Word variations have been searched) 1414279

#16 #1 OR #2 OR #3 OR #4 OR #5 260942

#17 #6 OR #7 OR #8 OR #9 OR #10 46065

#18 #11 OR #12 23924

#19 #13 OR #14 OR #15 1414334

#20 #16 AND #17 AND #18 AND #19 1256

#21 #16 AND #17 AND #18 AND #19 in Trials, English 1157

SCOPUS

TITLE-ABS-KEY ((child* OR neonat* OR newborn OR infant OR infancy OR infantile OR baby OR toddler OR kid OR juvenile OR pediatric* OR paediatric* OR preterm OR premature OR birthweight OR "scho ol age" OR "nursery school" OR preschool) AND (diarrh* OR rotavirus OR dehydrat* OR gastroenteritis) AND (probiotic* OR lactobacill* OR lactococc* OR bifidobacter* OR enterococc* OR streptococc* OR saccharomyces OR escherichia OR bacill*) AND (random* OR "RCT" OR trial)) AND (LIMIT-TO (DOCTYPE , "ar")) AND (LIMIT-TO (LANGUAGE , "English")) AND (EXCLUDE (SUBJAREA , "VETE")) AND (EXCLUDE (EXACTKEYWORD , "Animals") OR EXCLUDE (EXACTKEYWORD , "Animal"))

CINAHL

((MH "Child+") OR (MH "Infant+") OR (MH "Infant, Newborn, Diseases+") OR (MH "Pediatrics+") OR (MH "Schools, Nursery") OR child* OR child OR neonat* OR neonate OR infant OR infantile OR infancy OR baby OR toddler OR kid OR juvenile OR pediatric OR pediatric* OR paediatric* OR preterm OR premature OR “birth weight” OR birthweight OR “school age*” OR “nursery school” OR preschool) AND ((MH “Diarrhea”) OR (MH “HIV

Enteropathy”) OR (MH “Food Poisoning+”) OR (MH “Dehydration”) OR (MH “Rotavirus Infections”) OR (MH “Gastroenteritis+”) OR diarrh* OR rotavirus OR dehydrat* OR gastroenteritis) AND ((MH "Probiotics+") OR (MH "Bifidobacterium") OR (MH "Lactobacillus+") OR (MH "Streptococcus Salivarius") OR (MH "Saccharomyces") OR probiotic OR lactobacill* OR lactococc* OR bifidobacter* OR enterococc* OR streptococc* OR saccharomyces OR escherichia OR bacill*) AND ((MH "Random Assignment") OR RANDOM* OR RCT OR trial OR (MH "Randomized Controlled Trials+")) English Language; Human AND Apply related words; Apply equivalent subjects

clinicaltrials.gov

Interventional Studies | diarrh* OR rotavirus OR gastroenteritis OR dehydrat* | probiotic OR probiotics OR lactobacill* OR lactococc* OR bifidobacter* OR enterococc* OR streptococc* OR saccharomyces OR escherichia OR bacill* | Child

ICTRP

(child* OR neonat* OR infancy OR infant Or infantile OR baby OR babies OR toddler OR kid OR juvenile OR pediatric OR pediatric* OR paediatric* OR preterm OR premature OR birthweight OR "school age" OR "nursery school" OR preschool) AND (diarrh* OR rotavirus OR gastroenteritis OR dehydrat*) AND (probiotic OR probiotics OR lactobacill* OR lactococc* OR bifidobacter* OR enterococc* OR streptococc* OR saccharomyces OR escherichia OR bacill*)

Appendix S2

List of included studies

Abbaskhanian (2012)
Abbaskhanian, A., Rezai, M. S., Karami, H., & Hasnpour, A. (2012). The effect of fermented yogurt on rotavirus diarrhea in children. *6*(5), 1600-1604. https://www.cochranelibrary.com/central/doi/10.1002/central/CN-00904250/full

Agarwal (2002)
Agarwal, K. N., & Bhasin, S. K. (2002). Feasibility studies to control acute diarrhoea in children by feeding fermented milk preparations Actimel and Indian Dahi. *Eur J Clin Nutr*, *56 Suppl 4*, S56-59. https://doi.org/10.1038/sj.ejcn.1601664

Aggarwal (2014)
Aggarwal, S., Upadhyay, A., Shah, D., Teotia, N., Agarwal, A., & Jaiswal, V. (2014). Lactobacillus GG for treatment of acute childhood diarrhoea: an open labelled, randomized controlled trial. *Indian J Med Res*, *139*(3), 379-385.

Altcheh (2022)
Altcheh, J., Carosella, M. V., Ceballos, A., D'Andrea, U., Jofre, S. M., Marotta, C., Mugeri, D., Sabbaj, L., Soto, A., Josse, C., Montestruc, F., & McFarland, L. V. (2022). Randomized, direct comparison study of Saccharomyces boulardii CNCM I-745 versus multi-strained Bacillus clausii probiotics for the treatment of pediatric acute gastroenteritis. *Medicine (Baltimore)*, *101*(36), e30500. https://doi.org/10.1097/MD.0000000000030500

Basu (2007a)
Basu, S., Chatterjee, M., Ganguly, S., & Chandra, P. K. (2007a). Effect of Lactobacillus rhamnosus GG in persistent diarrhea in Indian children: a randomized controlled trial. *J Clin Gastroenterol*, *41*(8), 756-760. https://doi.org/10.1097/01.mcg.0000248009.47526.ea

Basu (2007b)
Basu, S., Chatterjee, M., Ganguly, S., & Chandra, P. K. (2007). Efficacy of Lactobacillus rhamnosus GG in acute watery diarrhoea of Indian children: a randomised controlled trial. *J Paediatr Child Health*, *43*(12), 837-842. https://doi.org/10.1111/j.1440-1754.2007.01201.x

Basu (2009)
Basu, S., Paul, D. K., Ganguly, S., Chatterjee, M., & Chandra, P. K. (2009). Efficacy of high-dose Lactobacillus rhamnosus GG in controlling acute watery diarrhea in Indian children: a randomized controlled trial. *J Clin Gastroenterol*, *43*(3), 208-213. https://doi.org/10.1097/MCG.0b013e31815a5780

Bhat (2018)
Bhat, S., Shreekrishna, G. N., & Savio, C. D. (2018). Efficacy of probiotics in acute diarrhoea in children. *Int J Contemp Pediatr*, *5*(4), 1646-1650.

Billoo (2006)
Billoo, A. G., Memon, M. A., Khaskheli, S. A., Murtaza, G., Iqbal, K., Saeed Shekhani, M., & Siddiqi, A. Q. (2006). Role of a probiotic (Saccharomyces boulardii) in management and prevention of diarrhoea. *World J Gastroenterol*, *12*(28), 4557-4560. https://doi.org/10.3748/wjg.v12.i28.4557

Boudraa (2001)
Boudraa, G., Benbouabdellah, M., Hachelaf, W., Boisset, M., Desjeux, J. F., & Touhami, M. (2001). Effect of feeding yogurt versus milk in children with acute diarrhea and carbohydrate malabsorption. *J Pediatr Gastroenterol Nutr*, *33*(3), 307-313. https://doi.org/10.1097/00005176-200109000-00015

Burande (2013)
Burande, M. (2013). Comparison of efficacy of Saccharomyces boulardii strain in the treatment of acute diarrhea in children: a prospective, single-blind, randomized controlled clinical trial. *4*(3), 205-208. https://doi.org/10.4103/0976-500X.114603

Burki (2017)
Burki, M. F. K., & Jabeen, F. (2017). Efficacy of saccharomyces boullardii in children with acute diarrhea. *28*(2), 112-116. https://www.cochranelibrary.com/central/doi/10.1002/central/CN-01404945/full

Canani (2007)
Canani, R. B., Cirillo, P., Terrin, G., Cesarano, L., Spagnuolo, M. I., De Vincenzo, A., Albano, F., Passariello, A., De Marco, G., Manguso, F., & Guarino, A. (2007). Probiotics for treatment of acute diarrhoea in children: randomised clinical trial of five different preparations. *BMJ*, *335*(7615), 340. https://doi.org/10.1136/bmj.39272.581736.55

Chen (2010)
Chen, C. C., Kong, M. S., Lai, M. W., Chao, H. C., Chang, K. W., Chen, S. Y., Huang, Y. C., Chiu, C. H., Li, W. C., Lin, P. Y., Chen, C. J., & Li, T. Y. (2010). Probiotics have clinical, microbiologic, and immunologic efficacy in acute infectious diarrhea. *Pediatr Infect Dis J*, *29*(2), 135-138. https://doi.org/10.1097/inf.0b013e3181b530bf

Chen (2023)
Chen, K., Jin, S., Ma, Y., Cai, L., Xu, P., Nie, Y., Luo, L., Yu, Q., Shen, Y., Ma, W., Zhou, Z., & Liu, C. (2023). Adjunctive efficacy of Lactis XLTG11 for Acute diarrhea in children: A randomized, blinded, placebo-controlled study. *Nutrition*, *111*, 112052. https://doi.org/10.1016/j.nut.2023.112052

Chen (2020)
Chen, K., Xin, J., Zhang, G., Xie, H., Luo, L., Yuan, S., Bu, Y., Yang, X., Ge, Y., & Liu, C. (2020). A combination of three probiotic strains for treatment of acute diarrhoea in hospitalised children: an open label, randomised controlled trial. *Benef Microbes*, *11*(4), 339-346. https://doi.org/10.3920/BM2020.0046

Correa (2011)
Corrêa, N. B., Penna, F. J., Lima, F. M., Nicoli, J. R., & Filho, L. A. (2011). Treatment of acute diarrhea with Saccharomyces boulardii in infants. *J Pediatr Gastroenterol Nutr*, *53*(5), 497-501. https://doi.org/10.1097/MPG.0b013e31822b7ab0

Costa-Ribeiro (2003)
Costa-Ribeiro, H., Ribeiro, T. C., Mattos, A. P., Valois, S. S., Neri, D. A., Almeida, P., Cerqueira, C. M., Ramos, E., Young, R. J., & Vanderhoof, J. A. (2003). Limitations of probiotic therapy in acute, severe dehydrating diarrhea. *J Pediatr Gastroenterol Nutr*, *36*(1), 112-115. https://doi.org/10.1097/00005176-200301000-00021

Cui (2016)
Cui, Y., Ye, P., Zhao, Y., Xu, H., Dong, J., Luo, J., & Fu, Y. (2016). Effect of bifidobacterium tetravaccine tablets-assisted therapy on T lymphocyte subpopulation of children with rotavirus enteritis. *9*(8), 15804-15811. https://www.cochranelibrary.com/central/doi/10.1002/central/CN-01727292/full

Dalgic (2011)
Dalgic, N., Sancar, M., Bayraktar, B., Pullu, M., & Hasim, O. (2011). Probiotic, zinc and lactose-free formula in children with rotavirus diarrhea: are they effective? *Pediatr Int*, *53*(5), 677-682. https://doi.org/10.1111/j.1442-200X.2011.03325.x

Das (2016)
Das, S., Gupta, P. K., & Das, R. R. (2016). Efficacy and Safety of Saccharomyces boulardii in Acute Rotavirus Diarrhea: Double Blind Randomized Controlled Trial from a Developing Country. *J Trop Pediatr*, *62*(6), 464-470. https://doi.org/10.1093/tropej/fmw032

Dhongade (2022)
Dhongade, A. R., Joshi, S. S., Kulkarni, S. V., Jadhav, S. S., Nargundkar, N., Deshmukh, D., & Mehta, A. D. (2022). The Efficacy and Safety of a Bacillus Probiotic Combination for the Treatment of Acute Diarrhea in Children: A Double-blind, Randomized, Placebo-controlled Multicentric Study. *Pediatric Infectious Disease*, *4*(3), 86-91.

Dinleyici (2015a)
Dinleyici, E. C., Dalgic, N., Guven, S., Metin, O., Yasa, O., Kurugol, Z., Turel, O., Tanir, G., Yazar, A. S., Arica, V., Sancar, M., Karbuz, A., Eren, M., Ozen, M., Kara, A., & Vandenplas, Y. (2015). Lactobacillus reuteri DSM 17938 shortens acute infectious diarrhea in a pediatric outpatient setting. *J Pediatr (Rio J)*, *91*(4), 392-396. https://doi.org/10.1016/j.jped.2014.10.009

Dinleyici (2015b)
Dinleyici, E. C., Kara, A., Dalgic, N., Kurugol, Z., Arica, V., Metin, O., Temur, E., Turel, O., Guven, S., Yasa, O., Bulut, S., Tanir, G., Yazar, A. S., Karbuz, A., Sancar, M., Erguven, M., Akca, G., Eren, M., Ozen, M., & Vandenplas, Y. (2015a). Saccharomyces boulardii CNCM I-745 reduces the duration of diarrhoea, length of emergency care and

hospital stay in children with acute diarrhoea. *Benef Microbes*, *6*(4), 415-421. https://doi.org/10.3920/BM2014.0086

Dinleyici (2014)
Dinleyici, E. C., & Vandenplas, Y. (2014). Lactobacillus reuteri DSM 17938 effectively reduces the duration of acute diarrhoea in hospitalised children. *Acta Paediatr*, *103*(7), e300-305. https://doi.org/10.1111/apa.12617

Dubey (2008)
Dubey, A. P., Rajeshwari, K., Chakravarty, A., & Famularo, G. (2008). Use of VSL[sharp]3 in the treatment of rotavirus diarrhea in children: preliminary results. *J Clin Gastroenterol*, *42 Suppl 3 Pt 1*, S126-129. https://doi.org/10.1097/MCG.0b013e31816fc2f6

Dutta (2011)
Dutta, P., Mitra, U., Dutta, S., Rajendran, K., Saha, T. K., & Chatterjee, M. K. (2011). Randomised controlled clinical trial of Lactobacillus sporogenes (Bacillus coagulans), used as probiotic in clinical practice, on acute watery diarrhoea in children. *Trop Med Int Health*, *16*(5), 555-561. https://doi.org/10.1111/j.1365-3156.2011.02745.x

El-Soud (2015)
El-Soud, N. H. A., Said, R. N., Mosallam, D. S., Barakat, N. A. M., & Sabry, M. A. (2015). Bifidobacterium lactis in treatment of children with acute diarrhea. A randomized double blind controlled trial. *3*(3), 403-407. https://doi.org/10.3889/oamjms.2015.088

Erdogan (2012)
Erdogan, O., Tanyeri, B., Torun, E., Gonullu, E., Arslan, H., Erenberk, U., & Oktem, F. (2012). The comparition of the efficacy of two different probiotics in rotavirusgastroenteritis in children. *J Trop Med*, *2012*, 787240. https://doi.org/10.1155/2012/787240

Eren (2010)
Eren, M., Dinleyici, E. C., & Vandenplas, Y. (2010). Clinical efficacy comparison of Saccharomyces boulardii and yogurt fluid in acute non-bloody diarrhea in children: a randomized, controlled, open label study. *Am J Trop Med Hyg*, *82*(3), 488-491. https://doi.org/10.4269/ajtmh.2010.09-0529

Francavilla (2012)

Francavilla, R., Lionetti, E., Castellaneta, S., Ciruzzi, F., Indrio, F., Masciale, A., Fontana, C., La Rosa, M. M., Cavallo, L., & Francavilla, A. (2012). Randomised clinical trial: Lactobacillus reuteri DSM 17938 vs. placebo in children with acute diarrhoea--a double-blind study. *Aliment Pharmacol Ther*, *36*(4), 363-369. https://doi.org/10.1111/j.1365-2036.2012.05180.x

Freedman (2015)

Freedman, S. B., Sherman, P. M., Willan, A., Johnson, D., Gouin, S., & Schuh, S. (2015). Emergency Department Treatment of Children With Diarrhea Who Attend Day Care: A Randomized Multidose Trial of a Lactobacillus helveticus and Lactobacillus rhamnosus Combination Probiotic. *Clin Pediatr (Phila)*, *54*(12), 1158-1166. https://doi.org/10.1177/0009922815569200

Freedman (2018)
Freedman, S. B., Williamson-Urquhart, S., Farion, K. J., Gouin, S., Willan, A. R., Poonai, N., Hurley, K., Sherman, P. M., Finkelstein, Y., Lee, B. E., Pang, X. L., Chui, L., Schnadower, D., Xie, J., Gorelick, M., & Schuh, S. (2018). Multicenter Trial of a Combination Probiotic for Children with Gastroenteritis. *N Engl J Med*, *379*(21), 2015-2026. https://doi.org/10.1056/NEJMoa1802597

Gaón (2003)
Gaón, D., García, H., Winter, L., Rodríguez, N., Quintás, R., González, S. N., & Oliver, G. (2003). Effect of Lactobacillus strains and Saccharomyces boulardii on persistent diarrhea in children. *Medicina (B Aires)*, *63*(4), 293-298.

Ghosh (2021)
Ghosh, A., Sundaram, B., Bhattacharya, P., Mohanty, N., Dheivamani, N., Mane, S., Acharyya, B., Kamale, V., Poddar, S., Khobragade, A., & et al. (2021). Effect of Saccharomyces boulardii CNCM-I 3799 and Bacillus subtilis CU-1 on Acute Watery Diarrhea: A Randomized Double-Blind Placebo-Controlled Study in Indian Children. *24*(5), 423-431. https://doi.org/10.5223/pghn.2021.24.5.423

Grandy (2010)
Grandy, G., Medina, M., Soria, R., Terán, C. G., & Araya, M. (2010). Probiotics in the treatment of acute rotavirus diarrhoea. A randomized, double-blind, controlled trial using two different probiotic preparations in Bolivian children. *BMC Infect Dis*, *10*, 253. https://doi.org/10.1186/1471-2334-10-253

Guandalini (2000)
Guandalini, S., Pensabene, L., Zikri, M. A., Dias, J. A., Casali, L. G., Hoekstra, H., Kolacek, S., Massar, K., Micetic-Turk, D., Papadopoulou, A., de Sousa, J. S., Sandhu, B., Szajewska, H., & Weizman, Z. (2000). Lactobacillus GG administered in oral rehydration solution to children with acute diarrhea: a multicenter European trial. *J Pediatr Gastroenterol Nutr*, *30*(1), 54-60. https://doi.org/10.1097/00005176-200001000-00018

Hamid (2019)
Hamid, F., Quaium, S. M. M. A., Rahman, A., Hussain, T., & Islam, T. M. D. T. (2019). Clinical efficacy of Bacillus clausii probiotic in the management of acute diarrhoea in children. *Chattagram Maa-O-Shishu Hospital Medical College Journal*, *18*(1), 14-17.

Hegar (2015)
Hegar, B., Waspada, I. M., Gunardi, H., & Vandenplas, Y. (2015). A double blind randomized trial showing probiotics to be ineffective in acute diarrhea in Indonesian

children. *Indian J Pediatr*, *82*(5), 410-414. https://doi.org/10.1007/s12098-014-1408-5

Henker (2007)
Henker, J., Laass, M., Blokhin, B. M., Bolbot, Y. K., Maydannik, V. G., Elze, M., Wolff, C., & Schulze, J. (2007). The probiotic Escherichia coli strain Nissle 1917 (EcN) stops acute diarrhoea in infants and toddlers. *Eur J Pediatr*, *166*(4), 311-318. https://doi.org/10.1007/s00431-007-0419-x

Henker (2008)
Henker, J., Laass, M. W., Blokhin, B. M., Maydannik, V. G., Bolbot, Y. K., Elze, M., Wolff, C., Schreiner, A., & Schulze, J. (2008). Probiotic Escherichia coli Nissle 1917 versus placebo for treating diarrhea of greater than 4 days duration in infants and toddlers. *Pediatr Infect Dis J*, *27*(6), 494-499. https://doi.org/10.1097/INF.0b013e318169034c

Heydarian (2010)
Heydarian, F., Kianifar, H. R., Ahanchian, H., Khakshure, A., Seyedi, J., & Moshirian, D. (2010). A comparison between traditional yogurt and probiotic yogurt in non-inflammatory acute gastroenteritis. *Saudi Med J*, *31*(3), 280-283.

Hong (2018)
Hong Chau, T. T., Minh Chau, N. N., Hoang Le, N. T., Chung The, H., Voong Vinh, P., Nguyen To, N. T., Ngoc, N. M., Tuan, H. M., Chau Ngoc, T. L., Kolader, M. E., Farrar, J. J., Wolbers, M., Thwaites, G. E., & Baker, S. (2018). A Double-blind, Randomized, Placebo-controlled Trial of Lactobacillus acidophilus for the Treatment of Acute Watery Diarrhea in Vietnamese Children. *Pediatr Infect Dis J*, *37*(1), 35-42. https://doi.org/10.1097/INF.0000000000001712

Huang (2014)
Huang, Y. F., Liu, P. Y., Chen, Y. Y., Nong, B. R., Huang, I. F., Hsieh, K. S., & Chen, K. T. (2014). Three-combination probiotics therapy in children with salmonella and rotavirus gastroenteritis. *J Clin Gastroenterol*, *48*(1), 37-42. https://doi.org/10.1097/MCG.0b013e31828f1c6e

Kesavelu (2022)
Kesavelu, D., Shyamala, J., & Kurup, A. (2022). Single-Strain Probiotics For The Management Of Acute Diarrhea In Children: a Randomized Comparative Study. *20*(9), 5277-5283. https://doi.org/10.14704/nq.2022.20.9.NQ44611

Khan (2012)
Khan, A., Javed, T., & Chishti, A. L. (2012). Clinical efficacy of use of probiotic "Saccharomyces boulardii" In children with acute watery diarrhea. *36*(3), 122-127. https://www.cochranelibrary.com/central/doi/10.1002/central/CN-00901880/full

Khan (2017)
Khan, M. A., Khattak, M. B., Munir, A., & Naz, I. (2017). Efficacy of lactobacillus reuteri in acute watery diarrhea. *28*(11), 7-11. https://www.cochranelibrary.com/central/doi/10.1002/central/CN-01441519/full

Khan (2021)
Khan, N. A., & Munir, S. S. (2021). To determine outcome of probiotics in treatment of acute diarrhoea in children. *15*(8), 1829-1831. https://doi.org/10.53350/pjmhs211581829

Khanna, V., Alam, S., & Malik, A. (2005). Efficacy of tyndalized Lactobacillus acidophilus in acute diarrhea. *Indian J Pediatr, 72*(11), 935-938. https://doi.org/10.1007/BF02731667

Kianifar (2009)
Kianifar, H. R., Farid, R., Ahanchian, H., Jabbari, F., Moghiman, T., & Sistanian, A. (2009). Probiotics in the treatment of acute diarrhea in young children. *34*(3), 204-207. https://www.cochranelibrary.com/central/doi/10.1002/central/CN-00754381/full

Kurugöl, Z., & Koturoğlu, G. (2005). Effects of Saccharomyces boulardii in children with acute diarrhoea. *Acta Paediatr, 94*(1), 44-47. https://doi.org/10.1111/j.1651-2227.2005.tb01786.x

Lahiri (2015a)
Lahiri, K., D'Souza, J., & Gahlowt, P. (2015a). Beneficial role of probiotic in acute childhood diarrhea. *J Harmoniz Res Med and Hlth Sci, 2*, 26-30.

Lahiri (2015b)
Lahiri, K., Jadhav, K., Gahlowt, P., Najmuddin, F., & Padmashree, Y. (2015). Bacillus clausii as an adjuvant therapy in acute childhood diarrhoea. *IOSR-JDMS, 14,* 74-76.

Lahiri (2008)
Lahiri, K. R. (2008). GMA-CO Clinical Study Report: ENTER_L_01486. *Sanofi-Aventis. Available online: https://www. sanofi. com/media/Project/One-Sanofi-Web/sanofi-com/common/docs/clinicalstudy-results/ENTER_L. _01486_summary. pdf (accessed on 19 April 2018).*

Lahiri (2022)
Lahiri, K. R., Singh, R., Apte, M., Patil, M., Taksande, A., Varona, R., Chatterjee, G., Verma, M., Brette, S., & Perez, M. I. (2022). Efficacy and safety of Bacillus clausii (O/C, N/R, SIN, T) probiotic combined with oral rehydration therapy (ORT) and zinc in acute diarrhea in children: a randomized, double-blind, placebo-controlled study in India. *8*(1), 9. https://doi.org/10.1186/s40794-022-00166-6

Lai (2019)
Lai, H. H., Chiu, C. H., Kong, M. S., Chang, C. J., & Chen, C. C. (2019). Probiotic Lactobacillus casei: Effective for Managing Childhood Diarrhea by Altering Gut Microbiota and Attenuating Fecal Inflammatory Markers. *Nutrients, 11*(5). https://doi.org/10.3390/nu11051150

Lee (2015)
Lee, D. K., Park, J. E., Kim, M. J., Seo, J. G., Lee, J. H., & Ha, N. J. (2015). Probiotic bacteria, B. longum and L. acidophilus inhibit infection by rotavirus in vitro and decrease

the duration of diarrhea in pediatric patients. *Clin Res Hepatol Gastroenterol*, *39*(2), 237-244. https://doi.org/10.1016/j.clinre.2014.09.006

Lee (2001)
Lee, M. C., Lin, L. H., Hung, K. L., & Wu, H. Y. (2001). Oral bacterial therapy promotes recovery from acute diarrhea in children. *Acta Paediatr Taiwan*, *42*(5), 301-305.

Lievin-Le Moal (2007)
Liévin-Le Moal, V., Sarrazin-Davila, L. E., & Servin, A. L. (2007). An experimental study and a randomized, double-blind, placebo-controlled clinical trial to evaluate the antisecretory activity of Lactobacillus acidophilus strain LB against nonrotavirus diarrhea. *Pediatrics*, *120*(4), e795-803. https://doi.org/10.1542/peds.2006-2930

Mahyar (2021)
Mahyar, A., Ayazi, P., Pashaei, H., Arad, B., Oveisi, S., & Esmaeili, S. (2021). The Effect of the Yeast Probiotic Saccharomyces boulardii on Acute Diarrhea in Children. *12*(4). https://doi.org/10.5812/compreped.117391

Mao (2008)
Mao, M., Yu, T., Xiong, Y., Wang, Z., Liu, H., Gotteland, M., & Brunser, O. (2008). Effect of a lactose-free milk formula supplemented with bifidobacteria and streptococci on the recovery from acute diarrhoea. *Asia Pac J Clin Nutr*, *17*(1), 30-34.

Maugo (2021)
Maugo, B., Jowi, C. Y., Murila, F., & Laving, A. (2021). EFFICACY OF BACILLUS CLAUSII IN REDUCING DURATION OF ILLNESS IN ACUTE DIARRHOEA IN CHILDREN 6-59 MONTHS OF AGE ADMITTED WITH SEVERE DEHYDRATION. *East African Medical Journal*, *98*(4).

Misra (2009)
Misra, S., Sabui, T. K., & Pal, N. K. (2009). A randomized controlled trial to evaluate the efficacy of lactobacillus GG in infantile diarrhea. *J Pediatr*, *155*(1), 129-132. https://doi.org/10.1016/j.jpeds.2009.01.060

Mourey (2020)
Mourey, F., Sureja, V., Kheni, D., Shah, P., Parikh, D., Upadhyay, U., Satia, M., Shah, D., Troise, C., & Decherf, A. (2020). A Multicenter, Randomized, Double-blind, Placebo-controlled Trial of Saccharomyces boulardii in Infants and Children With Acute Diarrhea. *Pediatr Infect Dis J*, *39*(11), e347-e351. https://doi.org/10.1097/INF.0000000000002849

Narayanappa (2008)
Narayanappa, D. (2008). Randomized double blinded controlled trial to evaluate the efficacy and safety of Bifilac in patients with acute viral diarrhea. *Indian J Pediatr*, *75*(7), 709-713. https://doi.org/10.1007/s12098-008-0134-2

Nixon (2012)
Nixon, A. F., Cunningham, S. J., Cohen, H. W., & Crain, E. F. (2012). The effect of Lactobacillus GG on acute diarrheal illness in the pediatric emergency department. *Pediatr*

Emerg Care, *28*(10), 1048-1051. https://doi.org/10.1097/PEC.0b013e31826cad9f

Ozkan (2007)
Ozkan, T. B., Sahin, E., Erdemir, G., & Budak, F. (2007). Effect of Saccharomyces boulardii in children with acute gastroenteritis and its relationship to the immune response. *J Int Med Res*, *35*(2), 201-212. https://doi.org/10.1177/147323000703500204

Park (2017)
Park, M. S., Kwon, B., Ku, S., & Ji, G. E. (2017). The Efficacy of Bifidobacterium longum BORI and Lactobacillus acidophilus AD031 Probiotic Treatment in Infants with Rotavirus Infection. *Nutrients*, *9*(8). https://doi.org/10.3390/nu9080887

Pashapour (2006)
Pashapour, N., & Iou, S. G. (2006). Evaluation of yogurt effect on acute diarrhea in 6-24-month-old hospitalized infants. *Turk J Pediatr*, *48*(2), 115-118.

Pernica (2022)
Pernica, J. M., Arscott-Mills, T., Steenhoff, A. P., Mokomane, M., Moorad, B., Bapabi, M., Lechiile, K., Mangwegape, O., Batisani, B., Mawoko, N., Muthoga, C., Vanniyasingam, T., Ewusie, J., Lowe, A., Bonsu, J. M., Gezmu, A. M., Smieja, M., Mazhani, L., Stordal, K., . . . Goldfarb, D. M. (2022). Optimising the management of childhood acute diarrhoeal disease using a rapid test-and- treat strategy and/or Lactobacillus reuteri DSM 17938: a multicentre, randomised, controlled, factorial trial in Botswana. *BMJ Glob Health*, *7*(4). https://doi.org/10.1136/bmjgh-2021-007826

Pernica (2017)
Pernica, J. M., Steenhoff, A. P., Mokomane, M., Moorad, B., Lechiile, K., Smieja, M., Mazhani, L., Cheng, J., Kelly, M. S., Loeb, M., Stordal, K., & Goldfarb, D. M. (2017). Rapid enteric testing to permit targeted antimicrobial therapy, with and without Lactobacillus reuteri probiotics, for paediatric acute diarrhoeal disease in Botswana: A pilot, randomized, factorial, controlled trial. *PLoS One*, *12*(10), e0185177. https://doi.org/10.1371/journal.pone.0185177

Phavichitr (2013)
Phavichitr, N., Puwdee, P., & Tantibhaedhyangkul, R. (2013). Cost-benefit analysis of the probiotic treatment of children hospitalized for acute diarrhea in Bangkok, Thailand. *Southeast Asian J Trop Med Public Health*, *44*(6), 1065-1071.

Rafeey (2008)
Rafeey, M., Ostadrahimi, A., Boniadi, M., Ghorashi, Z., Alizadeh, M. M., & Hadafey, V. (2008). Lactobacillus acidophilus yogurt and supplement in children with acute diarrhea: a clinical trial. *2*(1), 13-18. https://www.cochranelibrary.com/central/doi/10.1002/central/CN-00707630/full

Rerksuppaphol (2010)
Rerksuppaphol, S., & Rerksuppaphol, L. (2010). Lactobacillus acidophilus and Bifidobacterium bifidum stored at ambient temperature are effective in the treatment of acute diarrhoea. *Ann Trop Paediatr*, *30*(4), 299-304.

https://doi.org/10.1179/146532810X12858955921159

Riaz (2012)
Riaz, M., Alam, S., Malik, A., & Ali, S. M. (2012). Efficacy and safety of Saccharomyces boulardii in acute childhood diarrhea: a double blind randomised controlled trial. *Indian J Pediatr*, *79*(4), 478-482. https://doi.org/10.1007/s12098-011-0573-z

Ritchie (2010)
Ritchie, B. K., Brewster, D. R., Tran, C. D., Davidson, G. P., McNeil, Y., & Butler, R. N. (2010). Efficacy of Lactobacillus GG in aboriginal children with acute diarrhoeal disease: a randomised clinical trial. *J Pediatr Gastroenterol Nutr*, *50*(6), 619-624. https://doi.org/10.1097/MPG.0b013e3181bbf53d

Rosenfeldt (2002a)
Rosenfeldt, V., Michaelsen, K. F., Jakobsen, M., Larsen, C. N., Møller, P. L., Pedersen, P., Tvede, M., Weyrehter, H., Valerius, N. H., & Paerregaard, A. (2002). Effect of probiotic Lactobacillus strains in young children hospitalized with acute diarrhea. *Pediatr Infect Dis J*, *21*(5), 411-416. https://doi.org/10.1097/00006454-200205000-00012

Rosenfeldt (2002b)
Rosenfeldt, V., Michaelsen, K. F., Jakobsen, M., Larsen, C. N., Møller, P. L., Tvede, M., Weyrehter, H., Valerius, N. H., & Paerregaard, A. (2002a). Effect of probiotic Lactobacillus strains on acute diarrhea in a cohort of nonhospitalized children attending day-care centers. *Pediatr Infect Dis J*, *21*(5), 417-419. https://doi.org/10.1097/00006454-200205000-00013

Salazar-Lindo (2007)
Salazar-Lindo, E., Figueroa-Quintanilla, D., Caciano, M. I., Reto-Valiente, V., Chauviere, G., & Colin, P. (2007). Effectiveness and safety of Lactobacillus LB in the treatment of mild acute diarrhea in children. *J Pediatr Gastroenterol Nutr*, *44*(5), 571-576. https://doi.org/10.1097/MPG.0b013e3180375594

Salazar-Lindo (2004)
Salazar-Lindo, E., Miranda-Langschwager, P., Campos-Sanchez, M., Chea-Woo, E., & Sack, R. B. (2004). Lactobacillus casei strain GG in the treatment of infants with acute watery diarrhea: a randomized, double-blind, placebo controlled clinical trial. *4*, 18. https://doi.org/10.1186/1471-2431-4-18

Sarker (2005)
Sarker, S. A., Sultana, S., Fuchs, G. J., Alam, N. H., Azim, T., Brüssow, H., & Hammarström, L. (2005). Lactobacillus paracasei strain ST11 has no effect on rotavirus but ameliorates the outcome of nonrotavirus diarrhea in children from Bangladesh. *Pediatrics*, *116*(2), e221-228. https://doi.org/10.1542/peds.2004-2334

Schnadower (2018)
Schnadower, D., Tarr, P. I., Casper, T. C., Gorelick, M. H., Dean, J. M., O'Connell, K. J., Mahajan, P., Levine, A. C., Bhatt, S. R., Roskind, C. G., Powell, E. C., Rogers, A. J., Vance, C., Sapien, R. E., Olsen, C. S., Metheney, M., Dickey, V. P., Hall-Moore, C., &

Freedman, S. B. (2018). Lactobacillus rhamnosus GG versus Placebo for Acute Gastroenteritis in Children. *N Engl J Med*, *379*(21), 2002-2014. https://doi.org/10.1056/NEJMoa1802598

Sharif (2016)
Sharif, A. R., & Sharif, M. R. (2016). The effect of bacterial and yeast probiotics on acute watery diarrhea in children.

Sharma (2022)
Sharma, A. K., Puri, N., Mathur, M., & Mathur, A. (2022). A RANDOMIZED CONTROLL TRIAL TO COMPARE THE EFFICACY OF SACCHAROMYCES BOULARDII, BACILLUS CLAUSII, AND LACTOBACILLUS RHAMNOSUS GG PREPARATION IN THE TREATMENT OF ACUTE DIARRHEA IN CHILDREN. *15*(4), 63-67. https://doi.org/10.22159/ajpcr.2022.v15i4.44199

Sharma (2015)
Sharma, D., Choudhary, M., Beniwal, M., Dabi, D., & Pandita, A. (2015). Traditional Yoghurt and Probiotic in Treatment of Acute Childhood Diarrhoea: A Blinded Randomized Controlled Non - Inferiority Trial. *Journal of Pediatrics and Neonatal Care*, *2*. https://doi.org/10.15406/jpnc.2015.02.00058

Shin (2020)
Shin, D. Y., Yi, D. Y., Jo, S., Lee, Y. M., Kim, J. H., Kim, W., Park, M. R., Yoon, S. M., Kim, Y., Yang, S., & Lim, I. S. (2020). Effect of a new Lactobacillus plantarum product, LRCC5310, on clinical symptoms and virus reduction in children with rotaviral enteritis. *Medicine (Baltimore)*, *99*(38), e22192. https://doi.org/10.1097/MD.0000000000022192

Simakachorn (2000)
Simakachorn, N., Pichaipat, V., Rithipornpaisarn, P., Kongkaew, C., Tongpradit, P., & Varavithya, W. (2000). Clinical evaluation of the addition of lyophilized, heat-killed Lactobacillus acidophilus LB to oral rehydration therapy in the treatment of acute diarrhea in children. *J Pediatr Gastroenterol Nutr*, *30*(1), 68-72. https://doi.org/10.1097/00005176-200001000-00020

Sindhu (2014)
Sindhu, K. N., Sowmyanarayanan, T. V., Paul, A., Babji, S., Ajjampur, S. S., Priyadarshini, S., Sarkar, R., Balasubramanian, K. A., Wanke, C. A., Ward, H. D., & Kang, G. (2014). Immune response and intestinal permeability in children with acute gastroenteritis treated with Lactobacillus rhamnosus GG: a randomized, double-blind, placebo-controlled trial. *Clin Infect Dis*, *58*(8), 1107-1115. https://doi.org/10.1093/cid/ciu065

Singh (2018)
Singh, M., Alam, M. S., & Iqbal, S. M. J. (2018). Comparison of clinical efficacy of saccharomyces boulardii with yogurt in acute watery diarrhea in children. *42*(3), 167-171. https://www.cochranelibrary.com/central/doi/10.1002/central/CN-01738135/full

Sirsat (2017)

Sirsat, G. M., & Sankpal, D. M. (2017). Role of Saccharomyces boulardii in management of acute diarrhoea of children—A randomized controlled trial. *MedPulse Int. J. Pediatr, 4,* 68-72.

Sudarmo (2003)
Sudarmo, S. M., Ranuh, R. G., Rochim, A., & Soeparto, P. (2003). Management of infant diarrhea with high-lactose probiotic-containing formula. *Southeast Asian J Trop Med Public Health*, *34*(4), 845-848.

Sudha (2019)
Sudha, M. R., Jayanthi, N., Pandey, D. C., & Verma, A. K. (2019). Bacillus clausii UBBC-07 reduces severity of diarrhoea in children under 5 years of age: a double blind placebo controlled study. *Benef Microbes, 10*(2), 149-154. https://doi.org/10.3920/BM2018.0094

Szymanski (2006)
Szymański, H., Pejcz, J., Jawień, M., Chmielarczyk, A., Strus, M., & Heczko, P. B. (2006). Treatment of acute infectious diarrhoea in infants and children with a mixture of three Lactobacillus rhamnosus strains--a randomized, double-blind, placebo-controlled trial. *Aliment Pharmacol Ther*, *23*(2), 247-253. https://doi.org/10.1111/j.1365-2036.2006.02740.x

Szymanski (2019)
Szymański, H., & Szajewska, H. (2019). Lack of Efficacy of Lactobacillus reuteri DSM 17938 for the Treatment of Acute Gastroenteritis: A Randomized Controlled Trial. *Pediatr Infect Dis J*, *38*(10), e237-e242. https://doi.org/10.1097/INF.0000000000002355

Tabassum (2022)
Tabassum, S., Hanif, H. H., Riaz, S., Ahmed, N., & Ahsan, M. (2022). Saccharomyces Boulardii Vs. Yogurt Fluid for Acute Diarrhoea: a Clinical Efficacy Comparison. *16*(11), 436-438. https://doi.org/10.53350/pjmhs20221611436

Teran (2009)
Teran, C. G., Teran-Escalera, C. N., & Villarroel, P. (2009). Nitazoxanide vs. probiotics for the treatment of acute rotavirus diarrhea in children: a randomized, single-blind, controlled trial in Bolivian children. *Int J Infect Dis*, *13*(4), 518-523. https://doi.org/10.1016/j.ijid.2008.09.014

Vidjeadevan (2018)
Vidjeadevan, D., Vinoth, S., & Ramesh, S. (2018). Role of Saccharomyces boulardii and Bacillus clausii in reducing the duration of diarrhea: a three-armed randomised controlled trial. *Int J Contemp Pediatr*, *5*, 1811-1814.

Villarruel (2007)
Villarruel, G., Rubio, D. M., Lopez, F., Cintioni, J., Gurevech, R., Romero, G., & Vandenplas, Y. (2007). Saccharomyces boulardii in acute childhood diarrhoea: a randomized, placebo-controlled study. *Acta Paediatr*, *96*(4), 538-541. https://doi.org/10.1111/j.1651-2227.2007.00191.x

Risk of Bias assessment for all outcomes

Figure S1

Risk of Bias for clinical cure: Judgments about each risk of bias item for each included study

Study	D1	D2	D3	D4	D5	Overall
Chen 2020	+	-	-	+	!	-
Correa 2011	!	+	+	+	+	!
Cui 2016	!	!	+	-	+	-
Dinleyici 2015a	!	!	+	-	+	-
Dinleyici 2015b	!	!	+	-	!	-
Eren 2010	!	-	+	-	!	-
Henker 2007	!	+	+	+	+	!
Henker 2008	!	+	+	+	+	!
HongChau 2018	+	+	+	+	+	+
Khan 2012	!	+	+	-	+	-
Lahiri 2022	+	+	+	+	+	+
Lai 2019	!	-	+	!	!	!
Ritchie 2010	+	+	-	+	+	-
Villarruel 2007	!	-	-	+	!	-

Figure S2

Risk of Bias for clinical deterioration: Judgments about each risk of bias item for each included study

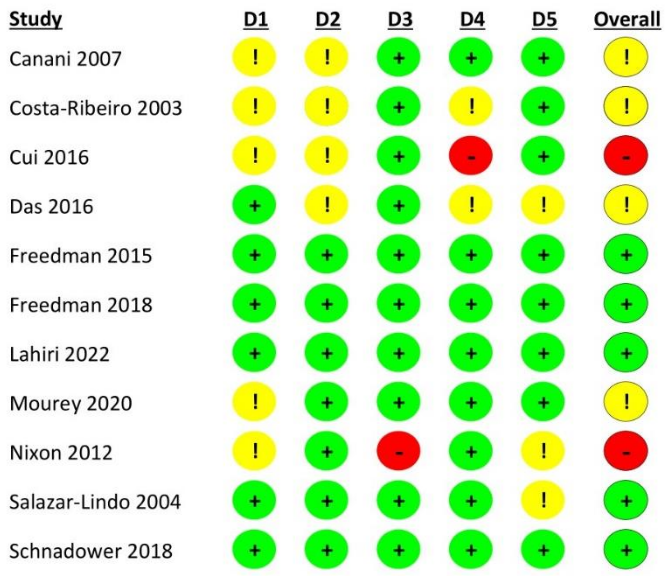

Figure S3

Risk of Bias for duration of diarrhea: Judgments about each risk of bias item for each included study

Study	D1	D2	D3	D4	D5	Overall
Agarwal 2002	!	!	+	!	!	!
Aggarwal 2014	!	!	-	-	!	-
Basu 2007b	+	+	+	+	+	+
Bhat 2018	!	!	+	-	+	-
Canani 2007	!	!	+	+	+	!
Chen 2010	!	+	+	+	+	!
Chen 2020	+	+	-	+	!	-
Costa-Ribeiro 2003	!	+	+	!	+	!
Dalgic 2011	!	!	+	!	!	!
Das 2016	+	!	+	!	!	!
Dhongade 2022	+	+	+	+	!	!
Dinleyici 2015	!	!	+	-	+	-
Dinleyici 2015a	!	!	-	-	!	-
El-Soud 2015	+	!	+	!	!	!
Erdogan 2012	!	!	+	-	!	-
Eren 2010	!	-	+	-	!	-
Freedman 2015	+	+	+	+	+	+
Freedman 2018	+	+	+	+	+	+
HongChau 2018	+	+	+	+	+	+
Huang 2014	!	!	+	-	!	-
Khan 2012	!	+	+	-	!	-
Khan 2021	!	-	+	-	!	-
Mao 2008	!	+	+	!	+	!
Maugo 2012	+	+	+	+	!	!
Misra 2009	+	+	+	+	!	!
Phavichitr 2013	+	+	+	!	!	!

Salazar-Lindo 2004	+	-	-	+	!	-
Salazar-Lindo 2007	+	+	!	+	+	!
Schnadower 2018	+	+	+	+	+	+
Sudarmo 2003	!	!	+	-	-	-
Sudha 2019	+	!	+	+	+	!
Teran 2009	-	!	+	-	!	-
Villarruel 2007	!	-	-	+	!	-

Figure S4

Risk of Bias for adverse events: Judgments about each risk of bias item for each included study

Study	D1	D2	D3	D4	D5	Overall
Aggarwal 2014	!	!	-	-	!	-
Basu 2007b	+	+	+	+	!	!
Basu 2009	+	+	+	+	!	!
Billoo 2006	!	!	+	-	!	-
Chen 2010	!	+	+	+	!	!
Chen 2023	+	+	+	+	!	!
Correa 2011	!	+	+	!	!	!
Dalgic 2011	!	!	+	!	!	!
Das 2016	+	!	+	-	!	-
Dhongade 2022	+	!	!	!	!	!
Dinleyici 2014	!	-	+	+	!	-
Dinleyici 2015	!	!	+	-	!	-
Dinleyici 2015b	!	!	+	-	!	-
Dutta 2011	+	+	+	!	!	!
El-Soud 2015	+	!	+	!	!	!
Francavilla 2012	!	+	+	+	!	!
Freedman 2018	+	+	+	+	+	+
Hegar 2015	+	+	+	+	!	!
Henker 2007	!	+	+	!	!	!
Henker 2008	!	+	+	+	!	!
HongChau 2018	+	+	+	+	+	+
Huang 2014	!	!	+	-	!	-
Kianifar 2009	!	+	+	+	-	-
Lahiri 2022	+	+	+	+	+	+
Lee 2015	!	+	+	!	!	!
Mahyar 2021	+	+	!	-	!	-

Mourey 2020	!	!	+	+	+	!
Narayanappa 2008	!	!	+	+	!	!
Nixon 2012	!	+	-	!	!	-
Ozkan 2007	!	!	+	!	!	!
Park 2017	!	!	+	!	!	!
Rerksuppaphol 2010	+	+	+	+	!	!
Riaz 2012	+	+	+	+	!	!
Ritchie 2010	+	+	-	+	!	-
Rosenfeldt 2002a	!	+	-	-	!	-
Salazar-Lindo 2004	+	+	+	+	!	!
Salazar-Lindo 2007	+	+	!	+	!	!
Schnadower 2018	+	+	+	+	+	+
Shin 2020	!	+	!	-	!	-
Szymanski 2006	!	+	+	!	!	!
Szymanski 2019	-	+	-	-	!	-
Teran 2009	-	!	+	-	!	-

Figure S5

Risk of Bias for serious adverse events: Judgments about each risk of bias item for each included study

Study	D1	D2	D3	D4	D5	Overall
Dhongade 2022	+	!	!	!	!	!
Freedman 2015	+	+	+	+	+	+
Freedman 2018	+	+	+	+	+	+
Ghosh 2021	+	!	+	+	!	!
Henker 2007	!	+	+	!	!	!
Henker 2008	!	+	+	+	!	!
Kurugol 2005	!	!	!	-	!	-
Lahiri 2022	+	+	+	+	+	+
Mourey 2020	!	!	+	+	+	!
Park 2017	!	!	+	!	!	!
Pernica 2017	+	+	+	+	!	!
Phavichitr 2013	+	+	+	!	!	!
Rosenfeldt 2002a	!	+	-	-	!	-
Schnadower 2018	+	+	+	+	+	+
Sindhu 2014	+	+	+	+	+	+

Forest plots for all outcomes

Figure S6

Forest plot for outcome: Clinical cure (n=44)

	Probiotic		Control			Risk Ratio
Study or Subgroup	Events	Total	Events	Total	Weight	M-H, Random, 95% CI
Boudraa 2001	47	56	46	56	2.5%	1.02 [0.86, 1.21]
Chen 2020	53	96	47	98	2.1%	1.15 [0.88, 1.51]
Chen 2023	20	35	9	35	0.9%	2.22 [1.18, 4.18]
Correa 2011	66	95	40	91	2.1%	1.58 [1.21, 2.07]
Cui 2016	35	43	42	43	2.6%	0.83 [0.72, 0.97]
Dinleyici 2014	56	64	46	63	2.5%	1.20 [1.00, 1.43]
Dinleyici 2015a	29	29	31	31	2.8%	1.00 [0.94, 1.07]
Dinleyici 2015b (1)	205	220	116	143	2.8%	1.15 [1.05, 1.25]
Dubey 2008	101	113	44	111	2.2%	2.25 [1.78, 2.86]
Dutta 2011	70	78	58	70	2.7%	1.08 [0.95, 1.23]
Eren 2010	23	28	21	27	2.1%	1.06 [0.81, 1.38]
Francavilla 2012	35	35	33	34	2.8%	1.03 [0.95, 1.12]
Guandalini 2000	143	147	125	140	2.8%	1.09 [1.02, 1.16]
Henker 2007	52	55	39	58	2.4%	1.41 [1.16, 1.70]
Henker 2008	74	75	54	76	2.6%	1.39 [1.20, 1.61]
Heydarian 2010	41	50	24	50	1.9%	1.71 [1.25, 2.34]
Hong 2018	140	150	139	150	2.8%	1.01 [0.95, 1.07]
Khan 2012	210	210	210	210	2.9%	1.00 [0.99, 1.01]
Khan 2017	30	48	19	48	1.5%	1.58 [1.05, 2.39]
Khan 2021	92	100	71	100	2.6%	1.30 [1.13, 1.49]
Khanna 2005	41	42	46	48	2.8%	1.02 [0.94, 1.10]
Kurugol 2005	99	100	96	100	2.9%	1.03 [0.99, 1.08]
Lahiri 2022	223	229	223	228	2.9%	1.00 [0.97, 1.02]
Lai 2019 (2)	39	42	33	39	2.6%	1.10 [0.94, 1.29]
Lee 2001	26	50	9	50	0.9%	2.89 [1.51, 5.53]
Lievin-Le Moal 2007	36	42	20	38	1.9%	1.63 [1.18, 2.26]
Mourey 2020	40	49	8	51	0.9%	5.20 [2.72, 9.97]
Nixon 2012	50	67	46	71	2.3%	1.15 [0.92, 1.44]
Rerksuppaphol 2010	36	45	11	22	1.4%	1.60 [1.03, 2.49]
Ritchie 2010	25	33	24	31	2.1%	0.98 [0.75, 1.28]
Rosenfeldt 2002a	27	30	26	39	2.2%	1.35 [1.05, 1.74]
Rosenfeldt 2002b	22	24	12	19	1.7%	1.45 [1.01, 2.09]
Salazar-Lindo 2004	52	90	51	89	2.2%	1.01 [0.78, 1.30]
Salazar-Lindo 2007	36	40	35	40	2.6%	1.03 [0.88, 1.20]
Sarker 2005	81	115	73	115	2.5%	1.11 [0.92, 1.33]
Sharma 2015	47	50	48	50	2.8%	0.98 [0.89, 1.07]
Shin 2020	14	15	4	8	0.8%	1.87 [0.92, 3.78]
Simakachorn 2000	36	37	27	36	2.4%	1.30 [1.07, 1.58]
Sirsat 2017	97	145	72	145	2.4%	1.35 [1.10, 1.65]
Sudha 2019	59	59	60	60	2.9%	1.00 [0.97, 1.03]
Szymanski 2006	43	46	34	41	2.6%	1.13 [0.96, 1.32]
Tabassum 2022	32	50	21	50	1.6%	1.52 [1.04, 2.24]
Vidjeadevan 2018	67	67	32	32	2.9%	1.00 [0.95, 1.05]
Villarruel 2007	35	44	21	44	1.8%	1.67 [1.18, 2.35]
**Total (95% CI)**		**3238**		**3080**	**100.0%**	**1.21 [1.12, 1.30]**
Total events	2785		2246			

Heterogeneity: $Tau^2 = 0.05$; $Chi^2 = 1675.89$, df = 43 ($P < 0.00001$); $I^2 = 97\%$

Test for overall effect: Z = 5.01 ($P < 0.00001$)

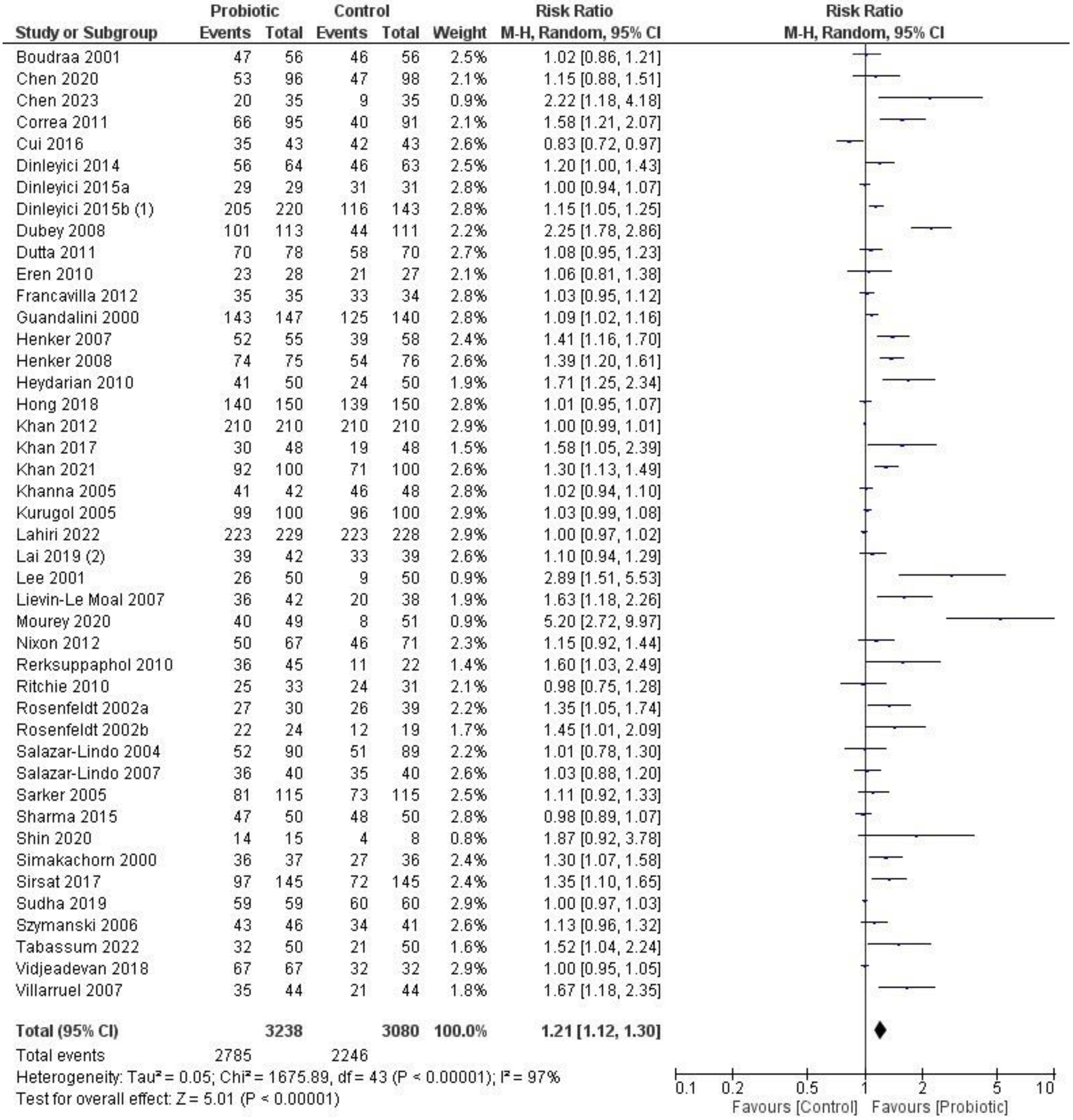

Footnotes

(1) Showed cumulative numbers for children from ED, hospitalized and outpatient settings

(2) Observed on Day 14

Figure S7

Forest plot for outcome: Clinical cure (Studies reporting WHO's definition of diarrhea; n=14)

	Probiotic		Control			Risk Ratio
Study or Subgroup	Events	Total	Events	Total	Weight	M-H, Random, 95% CI
Chen 2020	53	96	47	98	5.5%	1.15 [0.88, 1.51]
Correa 2011	66	95	40	91	5.6%	1.58 [1.21, 2.07]
Cui 2016	35	43	42	43	7.5%	0.83 [0.72, 0.97]
Dinleyici 2015a	29	29	31	31	8.7%	1.00 [0.94, 1.07]
Dinleyici 2015b (1)	205	220	116	143	8.5%	1.15 [1.05, 1.25]
Eren 2010	23	28	21	27	5.6%	1.06 [0.81, 1.38]
Henker 2007	52	55	39	58	6.9%	1.41 [1.16, 1.70]
Henker 2008	74	75	54	76	7.6%	1.39 [1.20, 1.61]
Hong 2018	140	150	139	150	8.7%	1.01 [0.95, 1.07]
Khan 2012	210	210	210	210	9.0%	1.00 [0.99, 1.01]
Lahiri 2022	223	229	223	228	8.9%	1.00 [0.97, 1.02]
Lai 2019 (2)	39	42	33	39	7.4%	1.10 [0.94, 1.29]
Ritchie 2010	25	33	24	31	5.5%	0.98 [0.75, 1.28]
Villarruel 2007	35	44	21	44	4.5%	1.67 [1.18, 2.35]
**Total (95% CI)**		**1349**		**1269**	**100.0%**	**1.12 [1.01, 1.24]**
Total events	1209		1040			

Heterogeneity: Tau² = 0.03; Chi² = 448.73, df = 13 (P < 0.00001); I² = 97%
Test for overall effect: Z = 2.13 (P = 0.03)

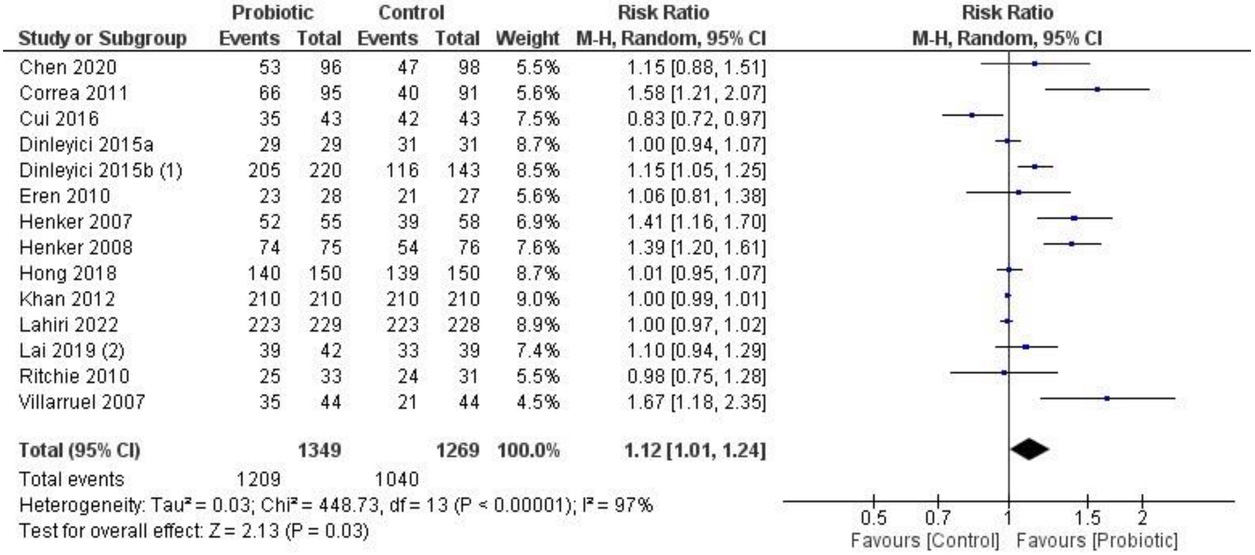

Footnotes
(1) Showed cumulative numbers for children from ED, hospitalized and outpatient settings.
(2) Observed on Day 14

Figure S8

Forest plot for sensitivity analysis for clinical cure based on ROB (WHO defined studies; n=5)

	Probiotic		Control			Risk Ratio
Study or Subgroup	Events	Total	Events	Total	Weight	M-H, Random, 95% CI
Correa 2011	66	95	40	91	15.9%	1.58 [1.21, 2.07]
Henker 2007	52	55	39	58	18.7%	1.41 [1.16, 1.70]
Henker 2008	74	75	54	76	20.2%	1.39 [1.20, 1.61]
Hong 2018	140	150	139	150	22.4%	1.01 [0.95, 1.07]
Lahiri 2022	223	229	223	228	22.8%	1.00 [0.97, 1.02]
**Total (95% CI)**		**604**		**603**	**100.0%**	**1.23 [1.01, 1.49]**
Total events	555		495			

Heterogeneity: Tau² = 0.04; Chi² = 96.87, df = 4 (P < 0.00001); I² = 96%
Test for overall effect: Z = 2.07 (P = 0.04)

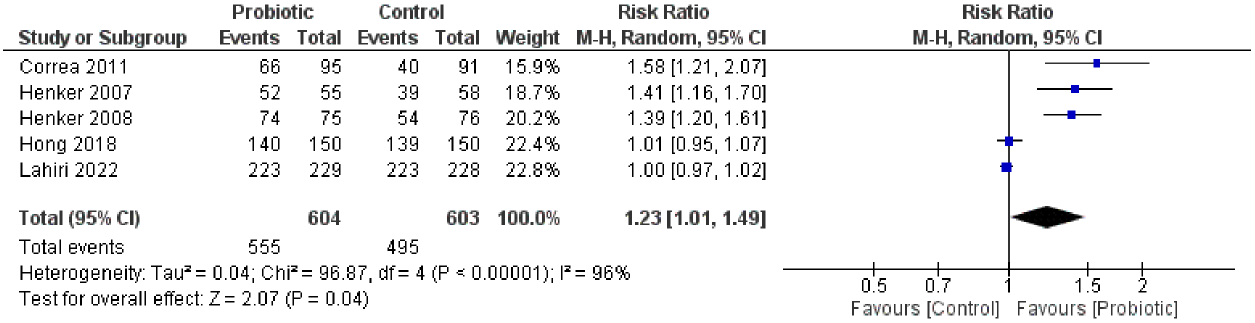

Figure S9

Forest plot for outcome: Clinical cure as reported at different timepoints (n=12)

	Probiotic		Control			Risk Ratio
Study or Subgroup	Events	Total	Events	Total	Weight	M-H, Random, 95% CI
**5.1.2 Day 3**						
Correa 2011	66	95	40	91	12.9%	1.58 [1.21, 2.07]
Cui 2016	35	43	42	43	13.8%	0.83 [0.72, 0.97]
Dinleyici 2015a	24	29	27	31	13.4%	0.95 [0.77, 1.18]
Dinleyici 2015b (1)	151	220	61	143	13.4%	1.61 [1.30, 1.98]
Khan 2012	199	210	90	210	13.8%	2.21 [1.89, 2.59]
Lahiri 2022	193	229	195	228	14.1%	0.99 [0.91, 1.06]
Lai 2019 (2)	13	42	6	39	6.9%	2.01 [0.85, 4.77]
Ritchie 2010	20	33	19	31	11.7%	0.99 [0.67, 1.46]
**Subtotal (95% CI)**		**901**		**816**	**100.0%**	**1.28 [0.93, 1.75]**
Total events	701		480			
Heterogeneity: $Tau^2 = 0.18$; $Chi^2 = 157.51$, df = 7 (P < 0.00001); $I^2 = 96\%$						
Test for overall effect: Z = 1.52 (P = 0.13)						
**5.1.3 Day 5**						
Dinleyici 2015a	29	29	31	31	17.9%	1.00 [0.94, 1.07]
Dinleyici 2015b (3)	205	220	116	143	14.9%	1.15 [1.05, 1.25]
Eren 2010	23	28	21	27	3.7%	1.06 [0.81, 1.38]
Hong 2018	140	150	139	150	18.1%	1.01 [0.95, 1.07]
Khan 2012	210	210	210	210	23.3%	1.00 [0.99, 1.01]
Lahiri 2022	223	229	223	228	22.1%	1.00 [0.97, 1.02]
**Subtotal (95% CI)**		**866**		**789**	**100.0%**	**1.02 [0.97, 1.08]**
Total events	830		740			
Heterogeneity: $Tau^2 = 0.00$; $Chi^2 = 48.54$, df = 5 (P < 0.00001); $I^2 = 90\%$						
Test for overall effect: Z = 0.81 (P = 0.42)						
**5.1.4 Day 7**						
Henker 2008	59	75	49	76	50.5%	1.22 [0.99, 1.50]
Lai 2019 (4)	34	42	23	39	27.6%	1.37 [1.02, 1.85]
Villarruel 2007	35	44	21	44	21.9%	1.67 [1.18, 2.35]
**Subtotal (95% CI)**		**161**		**159**	**100.0%**	**1.35 [1.14, 1.60]**
Total events	128		93			
Heterogeneity: $Tau^2 = 0.00$; $Chi^2 = 2.43$, df = 2 (P = 0.30); $I^2 = 18\%$						
Test for overall effect: Z = 3.42 (P = 0.0006)						
**5.1.5 Day 14**						
Lai 2019 (5)	39	42	33	39	100.0%	1.10 [0.94, 1.29]
**Subtotal (95% CI)**		**42**		**39**	**100.0%**	**1.10 [0.94, 1.29]**
Total events	39		33			
Heterogeneity: Not applicable						
Test for overall effect: Z = 1.15 (P = 0.25)						

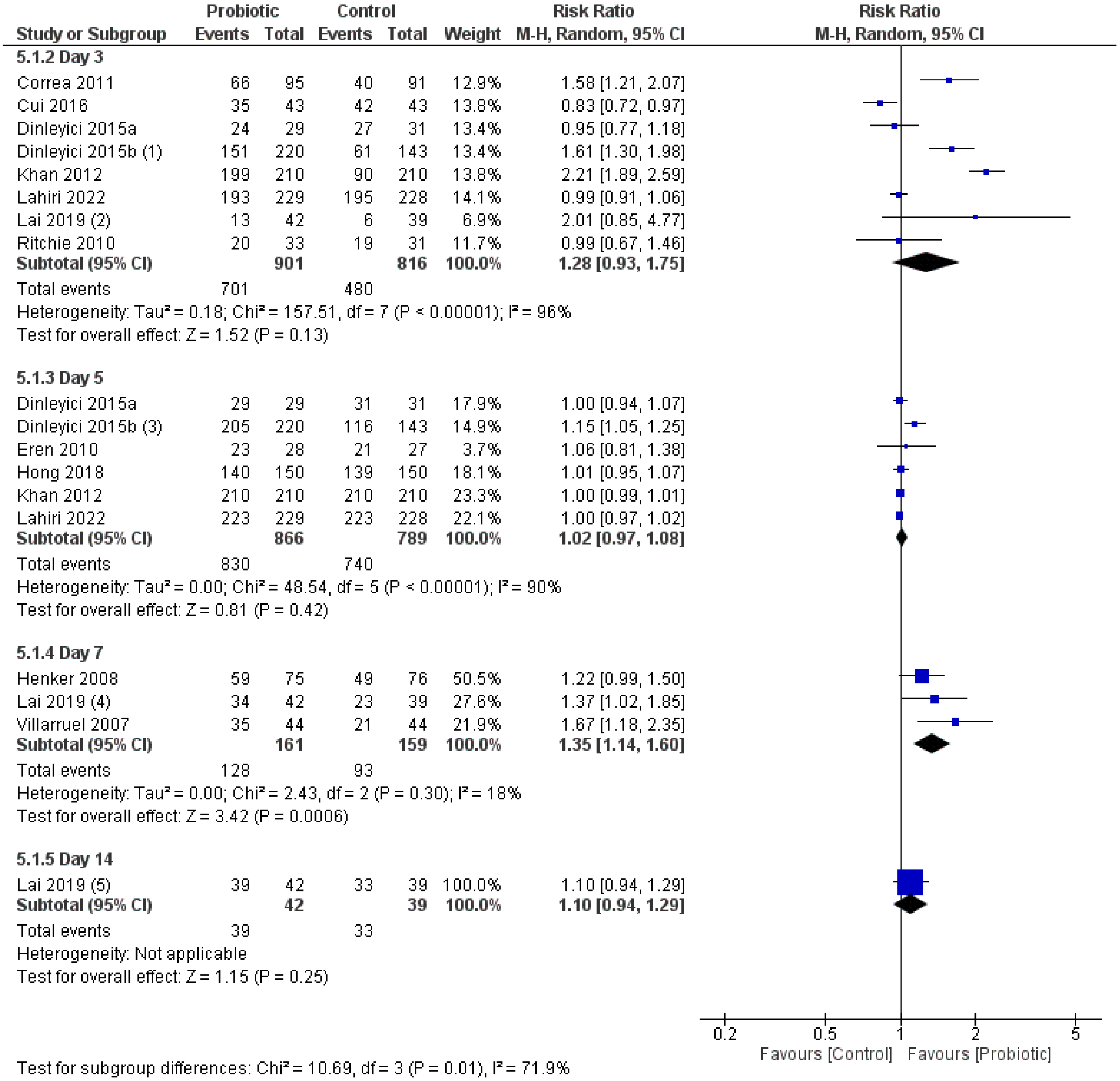

Test for subgroup differences: $Chi^2 = 10.69$, df = 3 (P = 0.01), $I^2 = 71.9\%$

Footnotes
(1) Showed cumulative numbers for children from ED, hospitalized and outpatient settings
(2) Observed on Day 14
(3) Showed cumulative numbers for children from ED, hospitalized and outpatient settings
(4) Observed on Day 14
(5) Observed on Day 14

Figure S10

Forest plot for outcome: Clinical Deterioration (n=18)

Study or Subgroup	Probiotic Events	Probiotic Total	Control Events	Control Total	Weight	Risk Ratio M-H, Random, 95% CI
Canani 2007	15	479	4	92	4.9%	0.72 [0.24, 2.12]
Costa-Ribeiro 2003	8	61	6	63	5.7%	1.38 [0.51, 3.74]
Cui 2016	8	43	1	43	1.4%	8.00 [1.04, 61.24]
Das 2016	2	30	5	28	2.4%	0.37 [0.08, 1.77]
Freedman 2015	1	61	0	62	0.6%	3.05 [0.13, 73.40]
Freedman 2018	33	414	22	413	18.8%	1.50 [0.89, 2.52]
Ghosh 2021	0	67	2	71	0.6%	0.21 [0.01, 4.33]
Guandalini 2000	12	147	11	140	9.0%	1.04 [0.47, 2.28]
Khanna 2005	4	48	0	50	0.7%	9.37 [0.52, 169.45]
Lahiri 2022	1	227	2	227	1.0%	0.50 [0.05, 5.48]
Mourey 2020	3	49	8	51	3.6%	0.39 [0.11, 1.39]
Nixon 2012	2	67	1	71	1.0%	2.12 [0.20, 22.83]
Riaz 2012	5	54	4	54	3.6%	1.25 [0.35, 4.40]
Salazar-Lindo 2004	19	90	16	89	14.9%	1.17 [0.65, 2.13]
Sarker 2005	1	115	4	115	1.2%	0.25 [0.03, 2.20]
Schnadower 2018	15	468	15	475	11.0%	1.01 [0.50, 2.05]
Sharma 2015	2	50	2	50	1.6%	1.00 [0.15, 6.82]
Singh 2018	20	143	25	143	17.7%	0.80 [0.47, 1.37]
**Total (95% CI)**		**2613**		**2237**	**100.0%**	**1.04 [0.82, 1.33]**
Total events	151		128			

Heterogeneity: Tau² = 0.01; Chi² = 17.70, df = 17 (P = 0.41); I² = 4%
Test for overall effect: Z = 0.35 (P = 0.73)

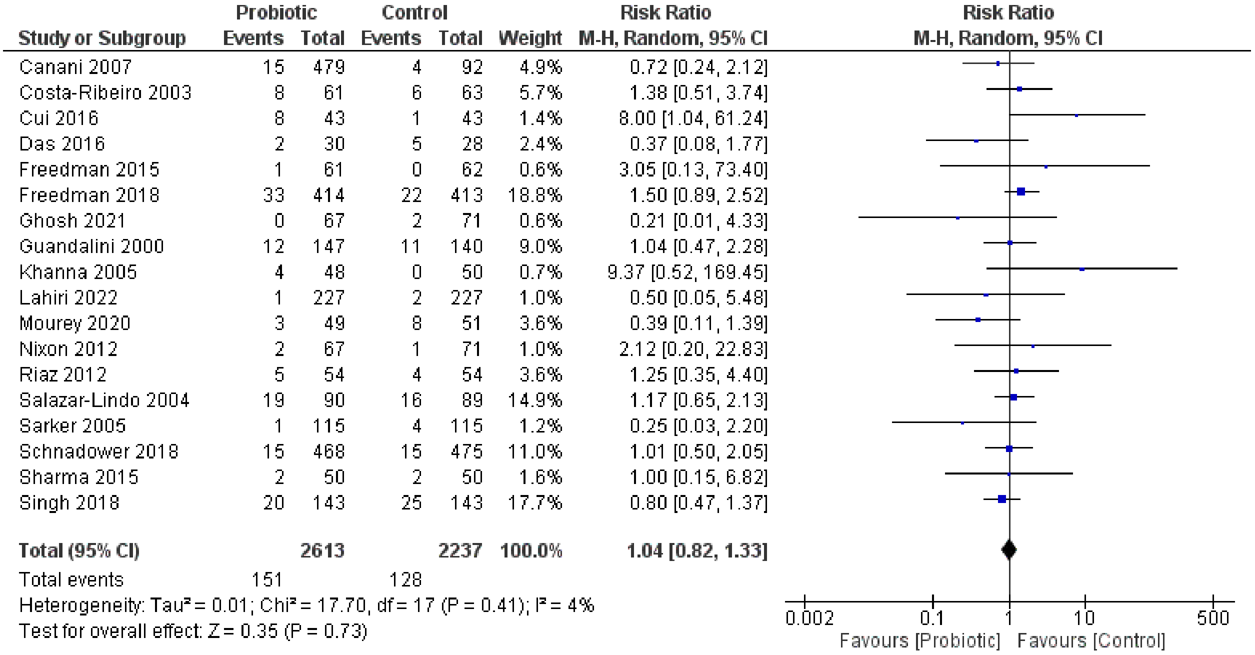

Figure S11

Forest plot for outcome: Clinical Deterioration (Studies reporting WHO's definition of diarrhea; n=11)

Study or Subgroup	Probiotic Events	Probiotic Total	Control Events	Control Total	Weight	Risk Ratio M-H, Random, 95% CI
Canani 2007	15	479	4	92	8.2%	0.72 [0.24, 2.12]
Costa-Ribeiro 2003	8	61	6	63	9.5%	1.38 [0.51, 3.74]
Cui 2016	8	43	1	43	2.5%	8.00 [1.04, 61.24]
Das 2016	2	30	5	28	4.2%	0.37 [0.08, 1.77]
Freedman 2015	1	61	0	62	1.0%	3.05 [0.13, 73.40]
Freedman 2018	33	414	22	413	26.2%	1.50 [0.89, 2.52]
Lahiri 2022	1	227	2	227	1.8%	0.50 [0.05, 5.48]
Mourey 2020	3	49	8	51	6.1%	0.39 [0.11, 1.39]
Nixon 2012	2	67	1	71	1.9%	2.12 [0.20, 22.83]
Salazar-Lindo 2004	19	90	16	89	21.7%	1.17 [0.65, 2.13]
Schnadower 2018	15	468	15	475	16.9%	1.01 [0.50, 2.05]
**Total (95% CI)**		**1989**		**1614**	**100.0%**	**1.12 [0.81, 1.55]**
Total events	107		80			

Heterogeneity: Tau² = 0.04; Chi² = 11.34, df = 10 (P = 0.33); I² = 12%
Test for overall effect: Z = 0.67 (P = 0.50)

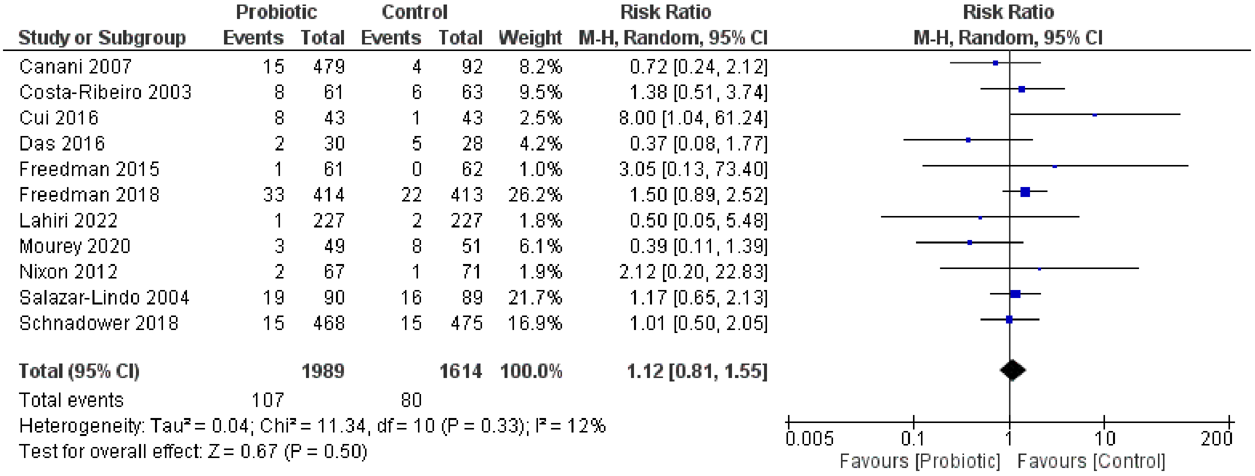

Figure S12

Forest plot for sensitivity analysis for Clinical Deterioration based on ROB (WHO-defined studies; n=6)

Study or Subgroup	Probiotic Events	Probiotic Total	Control Events	Control Total	Weight	Risk Ratio M-H, Random, 95% CI
Freedman 2015	1	61	0	62	1.1%	3.05 [0.13, 73.40]
Freedman 2018	33	414	22	413	39.1%	1.50 [0.89, 2.52]
Lahiri 2022	1	227	2	227	1.9%	0.50 [0.05, 5.48]
Mourey 2020	3	49	8	51	6.6%	0.39 [0.11, 1.39]
Salazar-Lindo 2004	19	90	16	89	29.9%	1.17 [0.65, 2.13]
Schnadower 2018	15	468	15	475	21.5%	1.01 [0.50, 2.05]
**Total (95% CI)**		**1309**		**1317**	**100.0%**	**1.16 [0.83, 1.60]**
Total events	72		63			

Heterogeneity: Tau² = 0.00; Chi² = 4.72, df = 5 (P = 0.45); I² = 0%
Test for overall effect: Z = 0.87 (P = 0.38)

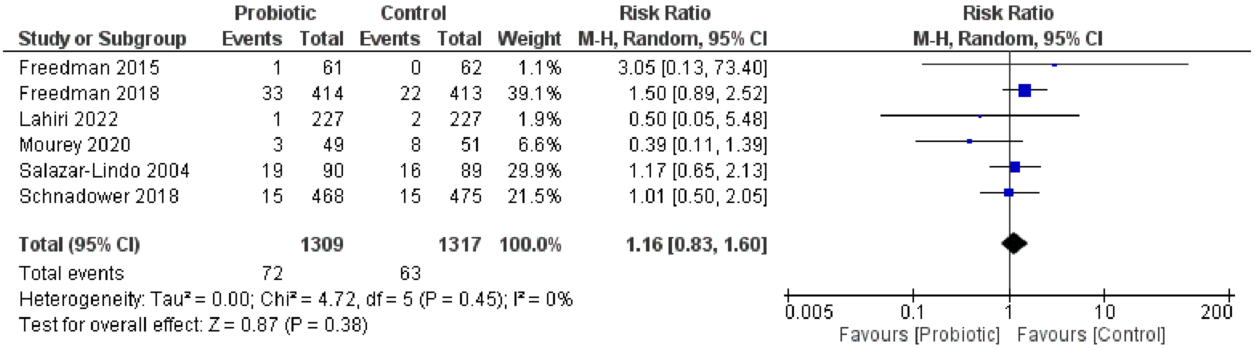

Figure S13

Forest plot for outcome: Mean duration of diarrhea (n= 67)

	Probiotic			Control				Mean Difference
Study or Subgroup	Mean [Hours]	SD [Hours]	Total	Mean [Hours]	SD [Hours]	Total	Weight	IV, Random, 95% CI
Abbaskhanian 2012	69.12	22.08	60	71.04	19.68	60	1.6%	-1.92 [-9.40, 5.56]
Agarwal 2002 (1)	39.888	13.6536	50	50.4	17.28	25	1.6%	-10.51 [-18.27, -2.75]
Agarwal 2002 (2)	49.2	21.816	50	57.6	21.6	25	1.5%	-8.40 [-18.80, 2.00]
Aggarwal 2014	62	13.54	100	80	13.539	100	1.8%	-18.00 [-21.75, -14.25]
Basu 2007b	163.2	50.4	323	158.4	55.2	323	1.6%	4.80 [-3.35, 12.95]
Basu 2009	121.6728	29.8152	374	173.52	30.48	185	1.7%	-51.85 [-57.18, -46.52]
Bhat 2018	47.505	15.2109	80	57.65	26.31	40	1.6%	-10.14 [-18.95, -1.34]
Billoo 2006	86.4	12.9	50	115.2	15.43	50	1.7%	-28.80 [-34.37, -23.23]
Boudraa 2001	44.1	34.36	56	61.7	36.27	56	1.3%	-17.60 [-30.69, -4.51]
Burki 2017	77.52	31.42	100	140.16	43.54	100	1.5%	-62.64 [-73.16, -52.12]
Canani 2007	96.3122	36.8789	479	112.56	23.95	92	1.7%	-16.25 [-22.15, -10.34]
Chen 2010	60.1	31.7	150	86.3	37.6	143	1.6%	-26.20 [-34.18, -18.22]
Chen 2020	121.4	13.7	96	143.9	19.8	98	1.8%	-22.50 [-27.28, -17.72]
Chen 2023	121.3	11.5	35	133.4	14.1	35	1.7%	-12.10 [-18.13, -6.07]
Costa-Ribeiro 2003	38.27	3.78	61	39.09	4.6	63	1.9%	-0.82 [-2.30, 0.66]
Dalgic 2011 (3)	74.64	43.44	60	81.84	33.12	60	1.3%	-7.20 [-21.02, 6.62]
Dalgic 2011 (4)	109.2	31.68	60	107.04	29.28	60	1.4%	2.16 [-8.76, 13.08]
Dalgic 2011 (5)	104.64	44.4	60	114	32.4	60	1.3%	-9.36 [-23.27, 4.55]
Dalgic 2011 (6)	114.72	35.04	60	128.4	43.2	60	1.3%	-13.68 [-27.75, 0.39]
Das 2016	59.33	12.45	30	84	21.091	28	1.6%	-24.67 [-33.66, -15.68]
Dhongade 2022	79.2	12.96	16	105.6	15.43	16	1.5%	-26.40 [-36.27, -16.53]
Dinleyici 2014	70.7	26.1	64	103.8	28.4	63	1.5%	-33.10 [-42.59, -23.61]
Dinleyici 2015a	60.4	24.5	29	74.3	15.3	31	1.5%	-13.90 [-24.32, -3.48]
Dinleyici 2015b (7)	83.1	36.3	148	114.8	35.4	72	1.5%	-31.70 [-41.75, -21.65]
Dinleyici 2015b (8)	60.4	23.1	25	78.4	20.9	26	1.4%	-18.00 [-30.11, -5.89]
Dinleyici 2015b (9)	58.9	15.3	47	88	19.1	45	1.7%	-29.10 [-36.19, -22.01]
Dutta 2011	34	20.4	78	36.5	21.4	70	1.7%	-2.50 [-9.25, 4.25]
El-Soud 2015	74.88	22.08	25	98.4	22.56	25	1.4%	-23.52 [-35.89, -11.15]
Erdogan 2012	128.4	47.016	50	168	38.4	25	0.9%	-39.60 [-59.51, -19.69]
Eren 2010	106.8	59.04	28	129.12	75.36	27	0.4%	-22.32 [-58.18, 13.54]
Francavilla 2012	50.4	40.8	35	79.2	50.4	34	0.9%	-28.80 [-50.47, -7.13]
Freedman 2015	71.1	78.31	61	63.5	64.3	62	0.7%	7.60 [-17.74, 32.94]
Freedman 2018	55.533	57.654	414	59.33	61.07	413	1.6%	-3.80 [-11.89, 4.30]
Ghosh 2021	54.16	38.2	67	59.48	40	71	1.3%	-5.32 [-18.37, 7.73]
Guandalini 2000	110.4	28.1	147	122.9	33	140	1.7%	-12.50 [-19.61, -5.39]
Hamid 2019	76.8	31.2	160	79.2	26.4	150	1.7%	-2.40 [-8.82, 4.02]
Hegar 2015	78.75	33.23	56	61.5	31.707	56	1.4%	17.25 [5.22, 29.28]
Hong 2018	80.33	42.667	150	79.66	41.17	150	1.5%	0.67 [-8.82, 10.16]
Huang 2014	43.2	38.4	82	69.4	33.6	77	1.4%	-26.20 [-37.40, -15.00]
Khan 2012	82.32	31.2	210	108	50.4	210	1.6%	-25.68 [-33.70, -17.66]
Khan 2021	144	34.08	100	144	38.16	100	1.5%	0.00 [-10.03, 10.03]
Khanna 2005	54.45	1.48	48	55.08	1.51	50	1.9%	-0.63 [-1.22, -0.04]
Kianifar 2009	81.6	19.2	32	108	19.2	30	1.5%	-26.40 [-35.96, -16.84]
Kurugol 2005	112.8	60	100	132	76.8	100	1.0%	-19.20 [-38.30, -0.10]
Lahiri 2008	48.6	38.2	0	56.1	40	0		Not estimable
Lahiri 2015a	22.26	20.4	80	34.16	21.4	80	1.7%	-11.90 [-18.38, -5.42]
Lahiri 2015b	22.64	20.4	69	47.05	21.4	62	1.7%	-24.41 [-31.59, -17.23]
Lee 2001	74.4	16.8	50	86.4	19.2	50	1.7%	-12.00 [-19.07, -4.93]
Lee 2015	146.4	12	13	172.8	45.6	16	0.8%	-26.40 [-49.68, -3.12]
Lievin-Le Moal 2007	39.5	10.5	42	63.4	14.9	38	1.7%	-23.90 [-29.60, -18.20]
Mao 2008	67.0071	3.6664	141	68	4	71	1.9%	-0.99 [-2.10, 0.12]
Maugo 2021	77.59	34.1	44	86.74	40.16	46	1.2%	-9.15 [-24.52, 6.22]
Misra 2009	70.56	31.2	105	78	26.4	105	1.6%	-7.44 [-15.26, 0.38]
Narayanappa 2008	104.4	30.048	40	130.8	40.656	40	1.2%	-26.40 [-42.07, -10.73]
Park 2017	105.15	30.96	28	134.64	29.52	29	1.2%	-29.49 [-45.20, -13.78]
Phavichitr 2013	103.92	58.896	53	120	36.576	53	1.0%	-16.08 [-34.74, 2.58]
Riaz 2012	52.08	24.57	54	64.04	30.43	54	1.5%	-11.96 [-22.39, -1.53]
Rosenfeldt 2002a	81.5	37.3	30	101.1	47.6	39	0.9%	-19.60 [-39.63, 0.43]
Rosenfeldt 2002b	75.9	39.7	24	115.7	85	19	0.4%	-39.80 [-81.19, 1.59]
Salazar-Lindo 2004	58.5	30.2	52	50.4	28	51	1.4%	8.10 [-3.14, 19.34]
Salazar-Lindo 2007	24.233	39.101	37	24.66	33.21	40	1.1%	-0.43 [-16.70, 15.84]
Sarker 2005	123.8	46	115	126.1	44.8	115	1.4%	-2.30 [-14.04, 9.44]
Schnadower 2018	51.633	50.268	468	54.7	46.99	475	1.7%	-3.07 [-9.28, 3.15]
Sharif 2016	76.08	36.3	30	106.32	35.4	30	1.0%	-30.24 [-48.38, -12.10]
Shin 2020	24	39.264	15	55.92	21.456	8	0.7%	-31.92 [-56.74, -7.10]
Simakachorn 2000	43.4	25.9	37	57	36.3	36	1.2%	-13.60 [-28.10, 0.90]
Sudarmo 2003	26.4	24	50	62.4	24	50	1.5%	-36.00 [-45.41, -26.59]
Sudha 2019	75.25	12.96	59	81.6	15.43	60	1.8%	-6.35 [-11.47, -1.23]
Szymanski 2006	83.6	55.6	46	96	71.5	41	0.7%	-12.40 [-39.55, 14.75]
Szymanski 2019	58.7	45.01	44	66.9	58.94	47	0.9%	-8.20 [-29.67, 13.27]
Teran 2009	57.1	25.4	25	74.6	26.6	25	1.2%	-17.50 [-31.92, -3.08]
Vidjeadevan 2018	83.9403	17.4709	67	108	18.62	32	1.6%	-24.06 [-31.75, -16.37]
Villarruel 2007	112.8	46.56	44	147.84	76.8	44	0.7%	-35.04 [-61.58, -8.50]
**Total (95% CI)**			**6368**			**5462**	**100.0%**	**-15.56 [-18.30, -12.82]**

Heterogeneity: Tau² = 104.25; Chi² = 1350.90, df = 71 (P < 0.00001); I² = 95%
Test for overall effect: Z = 11.13 (P < 0.00001)

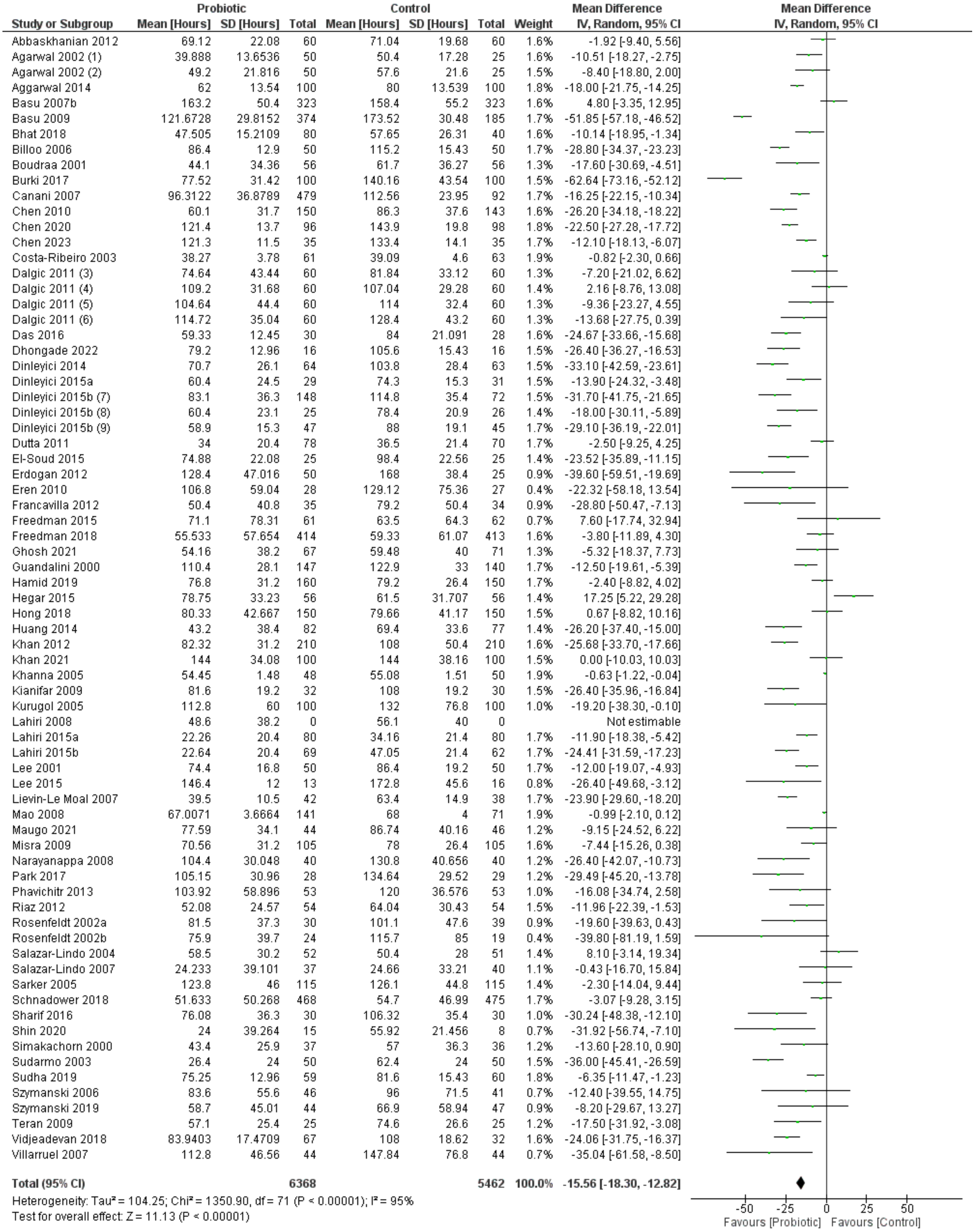

Footnotes
(1) Children in the hospital setting
(2) Children in the community setting
(3) Both arms given Zinc,ORS and/or IV rehydration
(4) Both arms given Lactose-free formula, ORS and/or IV rehydration
(5) Both arms given zinc, lactose-free formula, ORS and/or IV rehydration
(6) Both arms given ORS and/or IV rehydration
(7) Children in the hospital setting
(8) Children in the ED setting
(9) Children in the outpatient setting

Figure S14

Forest plot for outcome: Mean duration of diarrhea (Studies reporting WHO's definition of diarrhea; n=33)

	Probiotic			Control				Mean Difference
Study or Subgroup	Mean [Hours]	SD [Hours]	Total	Mean [Hours]	SD [Hours]	Total	Weight	IV, Random, 95% CI
Agarwal 2002 (1)	39.888	13.6536	50	50.4	17.28	25	3.0%	-10.51 [-18.27, -2.75]
Agarwal 2002 (2)	49.2	21.816	50	57.6	21.6	25	2.6%	-8.40 [-18.80, 2.00]
Aggarwal 2014	62	13.54	100	80	13.539	100	3.3%	-18.00 [-21.75, -14.25]
Basu 2007b	163.2	50.4	323	158.4	55.2	323	2.9%	4.80 [-3.35, 12.95]
Bhat 2018	47.505	15.2109	80	57.65	26.31	40	2.8%	-10.14 [-18.95, -1.34]
Canani 2007	96.3122	36.8789	479	112.56	23.95	92	3.2%	-16.25 [-22.15, -10.34]
Chen 2010	60.1	31.7	150	86.3	37.6	143	2.9%	-26.20 [-34.18, -18.22]
Chen 2020	121.4	13.7	96	143.9	19.8	98	3.3%	-22.50 [-27.28, -17.72]
Costa-Ribeiro 2003	38.27	3.78	61	39.09	4.6	63	3.5%	-0.82 [-2.30, 0.66]
Dalgic 2011 (3)	114.72	35.04	60	128.4	43.2	60	2.2%	-13.68 [-27.75, 0.39]
Dalgic 2011 (4)	74.64	43.44	60	81.84	33.12	60	2.2%	-7.20 [-21.02, 6.62]
Dalgic 2011 (5)	109.2	31.68	60	107.04	29.28	60	2.6%	2.16 [-8.76, 13.08]
Dalgic 2011 (6)	104.64	44.4	60	114	32.4	60	2.2%	-9.36 [-23.27, 4.55]
Das 2016	59.33	12.45	30	84	21.091	28	2.8%	-24.67 [-33.66, -15.68]
Dhongade 2022	79.2	12.96	16	105.6	15.43	16	2.7%	-26.40 [-36.27, -16.53]
Dinleyici 2015a	60.4	24.5	29	74.3	15.3	31	2.6%	-13.90 [-24.32, -3.48]
Dinleyici 2015b (7)	83.1	36.3	148	114.8	35.4	72	2.7%	-31.70 [-41.75, -21.65]
Dinleyici 2015b (8)	60.4	23.1	25	78.4	20.9	26	2.4%	-18.00 [-30.11, -5.89]
Dinleyici 2015b (9)	58.9	15.3	47	88	19.1	45	3.0%	-29.10 [-36.19, -22.01]
El-Soud 2015	74.88	22.08	25	98.4	22.56	25	2.4%	-23.52 [-35.89, -11.15]
Erdogan 2012	128.4	47.016	50	168	38.4	25	1.6%	-39.60 [-59.51, -19.69]
Eren 2010	106.8	59.04	28	129.12	75.36	27	0.7%	-22.32 [-58.18, 13.54]
Freedman 2015	71.1	78.31	61	63.5	64.3	62	1.2%	7.60 [-17.74, 32.94]
Freedman 2018	55.533	57.654	414	59.33	61.07	413	2.9%	-3.80 [-11.89, 4.30]
Hong 2018	80.33	42.667	150	79.66	41.17	150	2.8%	0.67 [-8.82, 10.16]
Huang 2014	43.2	38.4	82	69.4	33.6	77	2.5%	-26.20 [-37.40, -15.00]
Khan 2012	82.32	31.2	210	108	50.4	210	2.9%	-25.68 [-33.70, -17.66]
Khan 2021	144	34.08	100	144	38.16	100	2.7%	0.00 [-10.03, 10.03]
Mao 2008	67.0071	3.6664	141	68	4	71	3.5%	-0.99 [-2.10, 0.12]
Maugo 2021	77.59	34.1	44	86.74	40.16	46	2.1%	-9.15 [-24.52, 6.22]
Misra 2009	70.56	31.2	105	78	26.4	105	3.0%	-7.44 [-15.26, 0.38]
Phavichitr 2013	103.92	58.896	53	120	36.576	53	1.7%	-16.08 [-34.74, 2.58]
Salazar-Lindo 2004	58.5	30.2	52	50.4	28	51	2.5%	8.10 [-3.14, 19.34]
Salazar-Lindo 2007	24.233	39.101	37	24.66	33.21	40	2.0%	-0.43 [-16.70, 15.84]
Schnadower 2018	51.633	50.268	468	54.7	46.99	475	3.1%	-3.07 [-9.28, 3.15]
Sudarmo 2003	26.4	24	50	62.4	24	50	2.8%	-36.00 [-45.41, -26.59]
Sudha 2019	75.25	12.96	59	81.6	15.43	60	3.2%	-6.35 [-11.47, -1.23]
Teran 2009	57.1	25.4	25	74.6	26.6	25	2.2%	-17.50 [-31.92, -3.08]
Villarruel 2007	112.8	46.56	44	147.84	76.8	44	1.1%	-35.04 [-61.58, -8.50]
**Total (95% CI)**			**4122**			**3476**	**100.0%**	**-13.27 [-16.72, -9.83]**

Heterogeneity: Tau² = 88.80; Chi² = 468.34, df = 38 (P < 0.00001); I² = 92%
Test for overall effect: Z = 7.55 (P < 0.00001)

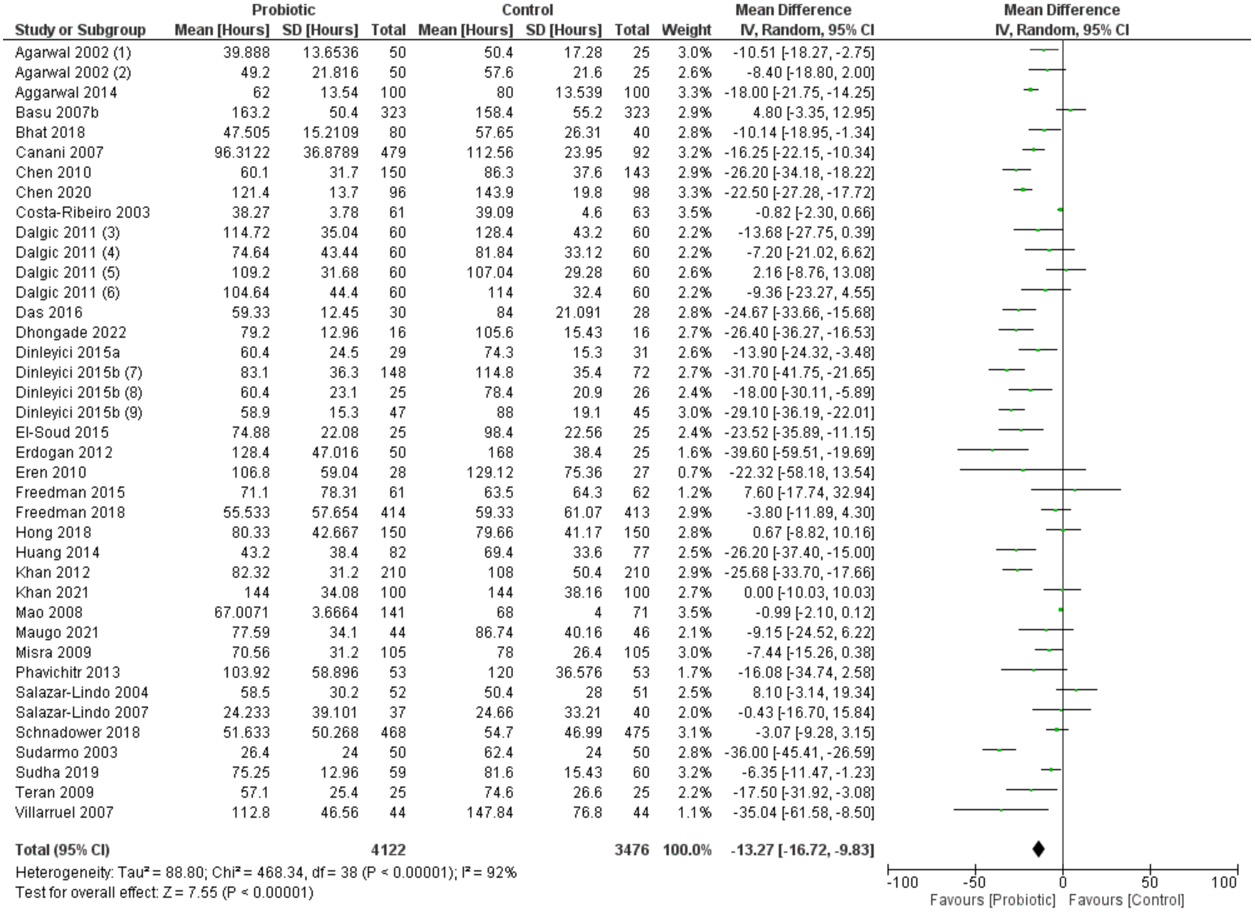

Footnotes
(1) Children in the hospital setting
(2) Children in the community setting
(3) Both arms given ORS and/or IV rehydration
(4) Both arms given Zinc,ORS and/or IV rehydration
(5) Both arms given Lactose-free formula, ORS and/or IV rehydration
(6) Both arms given zinc, lactose-free formula, ORS and/or IV rehydration
(7) Children in the hospital setting
(8) Children in the ED setting
(9) Children in the outpatient setting

Figure S15

Forest plot for sensitivity analysis for duration of diarrhea based on ROB (WHO defined studies; n=11)

	Probiotic			Control				Mean Difference	Mean Difference
**Study or Subgroup**	**Mean [Hours]**	**SD [Hours]**	**Total**	**Mean [Hours]**	**SD [Hours]**	**Total**	**Weight**	**IV, Random, 95% CI**	**IV, Random, 95% CI**
Basu 2007b	163.2	50.4	323	158.4	55.2	323	10.2%	4.80 [-3.35, 12.95]	
Chen 2010	60.1	31.7	150	86.3	37.6	143	10.3%	-26.20 [-34.18, -18.22]	
Dhongade 2022	79.2	12.96	16	105.6	15.43	16	9.4%	-26.40 [-36.27, -16.53]	
Freedman 2015	71.1	78.31	61	63.5	64.3	62	4.0%	7.60 [-17.74, 32.94]	
Freedman 2018	55.533	57.654	414	59.33	61.07	413	10.2%	-3.80 [-11.89, 4.30]	
Hong 2018	80.33	42.667	150	79.66	41.17	150	9.6%	0.67 [-8.82, 10.16]	
Maugo 2021	77.59	34.1	44	86.74	40.16	46	7.0%	-9.15 [-24.52, 6.22]	
Misra 2009	70.56	31.2	105	78	26.4	105	10.3%	-7.44 [-15.26, 0.38]	
Salazar-Lindo 2007	24.233	39.101	37	24.66	33.21	40	6.7%	-0.43 [-16.70, 15.84]	
Schnadower 2018	51.633	50.268	468	54.7	46.99	475	11.0%	-3.07 [-9.28, 3.15]	
Sudha 2019	75.25	12.96	59	81.6	15.43	60	11.4%	-6.35 [-11.47, -1.23]	
**Total (95% CI)**			**1827**			**1833**	**100.0%**	**-7.20 [-13.36, -1.03]**	

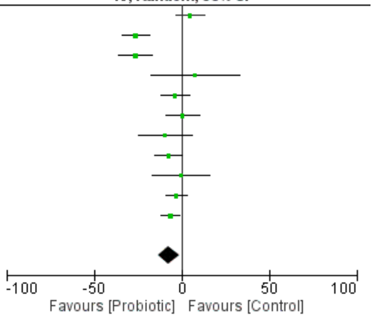

Heterogeneity: Tau² = 79.97; Chi² = 51.79, df = 10 (P < 0.00001); I² = 81%

Test for overall effect: Z = 2.29 (P = 0.02)

Figure S16

Forest plot for outcome: Adverse events (n= 42)

Study or Subgroup	Probiotic Events	Probiotic Total	Control Events	Control Total	Weight	Risk Ratio M-H, Random, 95% CI
Aggarwal 2014	0	100	0	100		Not estimable
Basu 2007b (1)	5	330	5	332	1.3%	1.01 [0.29, 3.44]
Basu 2009 (2)	12	392	7	196	2.4%	0.86 [0.34, 2.14]
Billoo 2006 (3)	0	50	0	50		Not estimable
Chen 2010	0	150	0	143		Not estimable
Chen 2023	0	35	0	35		Not estimable
Correa 2011	0	95	0	91		Not estimable
Dalgic 2011 (4)	0	60	0	60		Not estimable
Dalgic 2011 (5)	0	60	0	60		Not estimable
Dalgic 2011 (6)	0	60	0	60		Not estimable
Dalgic 2011 (7)	0	60	0	60		Not estimable
Das 2016	0	30	0	28		Not estimable
Dhongade 2022	0	16	0	16		Not estimable
Dinleyici 2014	0	64	0	63		Not estimable
Dinleyici 2015a	0	29	0	31		Not estimable
Dinleyici 2015b (8)	0	220	0	143		Not estimable
Dutta 2011	0	78	0	70		Not estimable
El-Soud 2015	0	25	0	25		Not estimable
Francavilla 2012	0	35	0	34		Not estimable
Freedman 2018	144	414	160	413	62.9%	0.90 [0.75, 1.07]
Hegar 2015	0	56	0	56		Not estimable
Henker 2007	2	55	2	58	0.5%	1.05 [0.15, 7.23]
Henker 2008	3	75	4	76	0.9%	0.76 [0.18, 3.28]
Hong 2018	0	150	0	150		Not estimable
Huang 2014	0	82	0	77		Not estimable
Kianifar 2009	0	32	0	30		Not estimable
Lahiri 2022	22	227	28	227	7.3%	0.79 [0.46, 1.33]
Lee 2015	0	13	0	16		Not estimable
Mahyar 2021	0	30	0	30		Not estimable
Mourey 2020	7	49	9	51	2.5%	0.81 [0.33, 2.00]
Narayanappa 2008	0	40	0	40		Not estimable
Nixon 2012	0	67	0	71		Not estimable
Ozkan 2007	0	16	0	11		Not estimable
Park 2017	0	28	0	29		Not estimable
Rerksuppaphol 2010	0	45	0	22		Not estimable
Riaz 2012	0	54	0	54		Not estimable
Ritchie 2010	0	33	0	31		Not estimable
Rosenfeldt 2002b	1	24	0	19	0.2%	2.40 [0.10, 55.79]
Salazar-Lindo 2004	0	90	0	89		Not estimable
Salazar-Lindo 2007	12	40	6	40	2.6%	2.00 [0.83, 4.81]
Schnadower 2018	63	472	62	479	18.9%	1.03 [0.74, 1.43]
Shin 2020	0	15	0	8		Not estimable
Szymanski 2006	0	46	0	41		Not estimable
Szymanski 2019	3	44	1	47	0.4%	3.20 [0.35, 29.67]
Teran 2009	0	25	0	25		Not estimable
**Total (95% CI)**		**4111**		**3787**	**100.0%**	**0.94 [0.81, 1.08]**
Total events	274		284			

Heterogeneity: Tau² = 0.00; Chi² = 5.63, df = 10 (P = 0.85); I² = 0%

Test for overall effect: Z = 0.91 (P = 0.36)

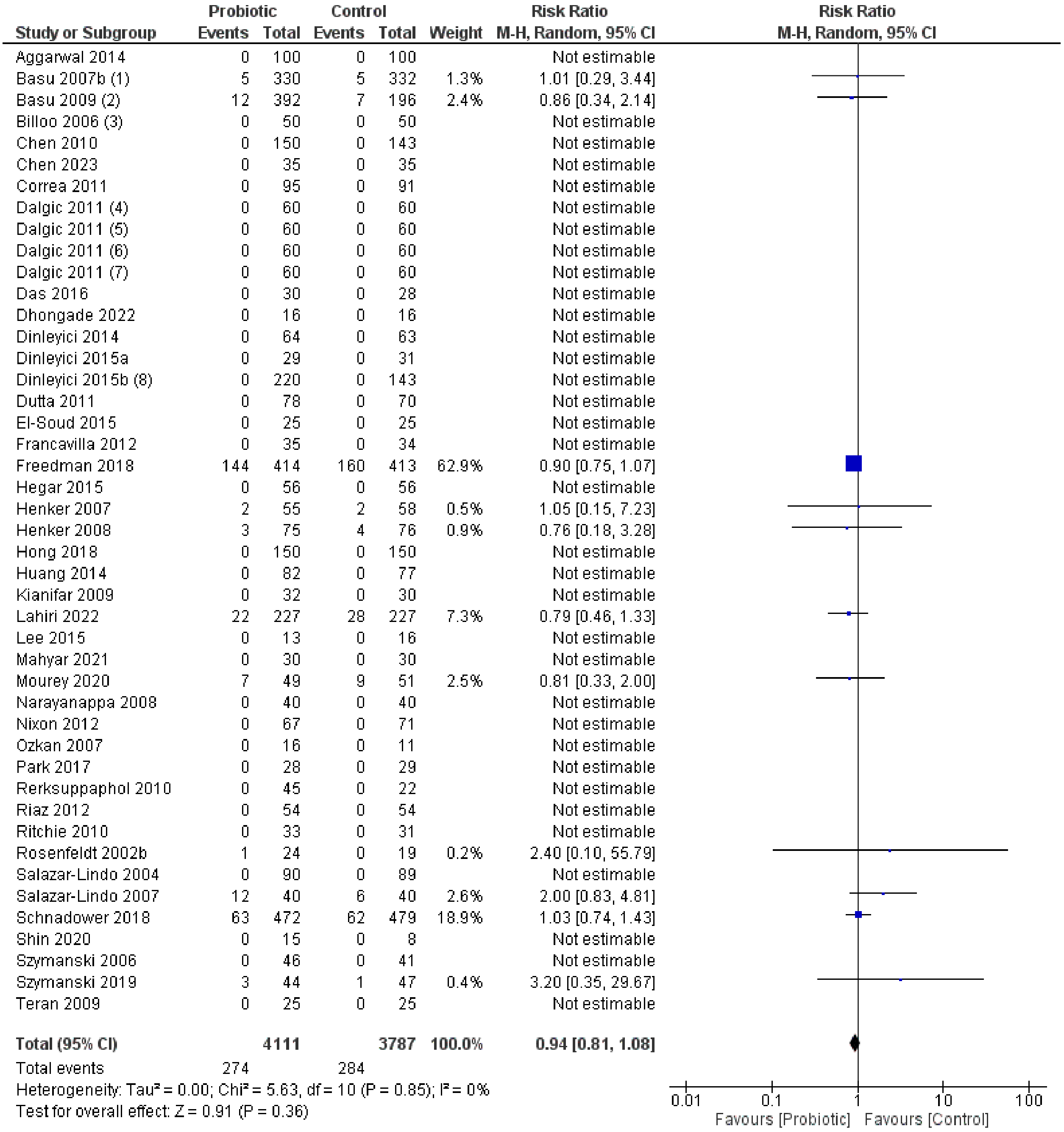

Footnotes

(1) Denominator denotes allocated participants. Authors reported electrolyte imbalance and septicemia as complications

(2) Denominator denotes allocated participants. Although authors reported no adverse effect of the probiotic, electrolyte imbalance and...

(3) Authors reported no side effects due to probiotic during the study period

(4) Both arms given Zinc,ORS and/or IV rehydration

(5) Both arms given Lactose-free formula, ORS and/or IV rehydration

(6) Both arms given zinc, lactose-free formula, ORS and/or IV rehydration

(7) Both arms given ORS and/or IV rehydration

(8) Showed cumulative numbers for children from ED, hospitalized and outpatient settings

Figure S17

Forest plot for sensitivity analysis for adverse events based on ROB (WHO defined studies; n=11)

Study or Subgroup	Probiotic Events	Probiotic Total	Control Events	Control Total	Weight	Risk Ratio M-H, Random, 95% CI
Basu 2007b (1)	5	330	5	332	1.4%	1.01 [0.29, 3.44]
Basu 2009 (2)	12	392	7	196	2.6%	0.86 [0.34, 2.14]
Chen 2023	0	35	0	35		Not estimable
Freedman 2018	144	414	160	413	67.8%	0.90 [0.75, 1.07]
Hegar 2015	0	56	0	56		Not estimable
Hong 2018	0	150	0	150		Not estimable
Lahiri 2022	22	227	28	227	7.8%	0.79 [0.46, 1.33]
Rerksuppaphol 2010	0	45	0	22		Not estimable
Riaz 2012	0	54	0	54		Not estimable
Salazar-Lindo 2004	0	90	0	89		Not estimable
Schnadower 2018	63	472	62	479	20.4%	1.03 [0.74, 1.43]
**Total (95% CI)**		**2265**		**2053**	**100.0%**	**0.91 [0.79, 1.06]**
Total events	246		262			

Heterogeneity: Tau² = 0.00; Chi² = 0.92, df = 4 (P = 0.92); I² = 0%
Test for overall effect: Z = 1.19 (P = 0.23)

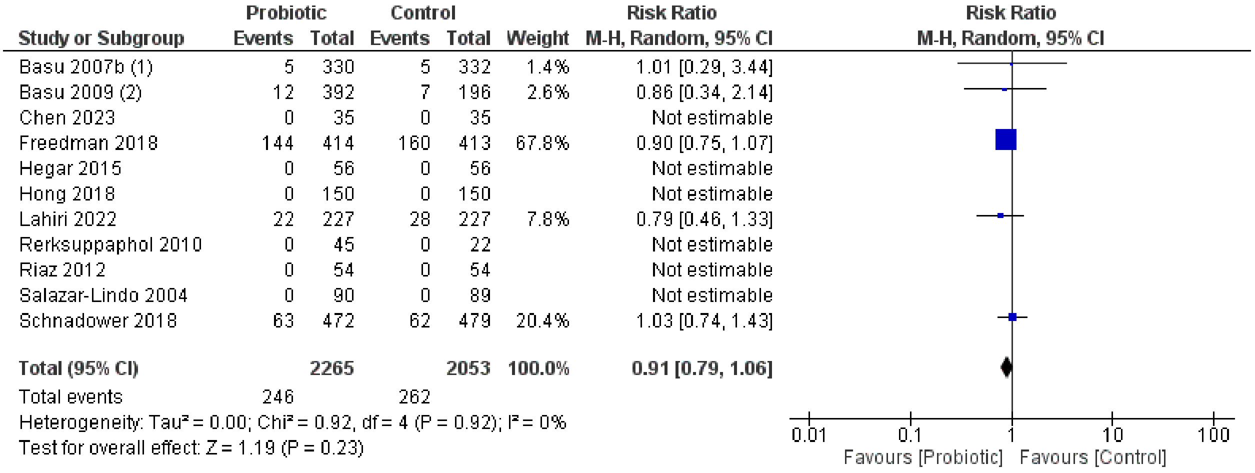

Footnotes
(1) Denominator denotes allocated participants. Authors reported electrolyte imbalance and septicemia as complications
(2) Denominator denotes allocated participants. Although authors reported no adverse effect of the probiotic, electrolyte imbalance and...

Figure S18

Forest plot for outcome: Serious adverse events (n= 15)

Study or Subgroup	Probiotic Events	Probiotic Total	Placebo Events	Placebo Total	Weight	Risk Ratio M-H, Random, 95% CI
Dhongade 2022	0	16	0	16		Not estimable
Freedman 2015	0	61	0	62		Not estimable
Freedman 2018	0	414	2	413	9.6%	0.20 [0.01, 4.14]
Ghosh 2021	0	67	2	71	9.7%	0.21 [0.01, 4.33]
Henker 2007	0	55	0	58		Not estimable
Henker 2008	0	75	0	76		Not estimable
Kurugol 2005	0	100	0	100		Not estimable
Lahiri 2022	0	227	0	227		Not estimable
Mourey 2020	0	49	0	51		Not estimable
Park 2017	0	28	0	29		Not estimable
Pernica 2017 (1)	0	15	0	15		Not estimable
Pernica 2017 (2)	0	12	0	19		Not estimable
Phavichitr 2013	0	53	0	53		Not estimable
Rosenfeldt 2002b	0	24	0	19		Not estimable
Schnadower 2018	8	472	13	479	63.1%	0.62 [0.26, 1.49]
Sindhu 2014	4	65	1	59	17.6%	3.63 [0.42, 31.57]
**Total (95% CI)**		**1733**		**1747**	**100.0%**	**0.69 [0.26, 1.83]**
Total events	12		18			

Heterogeneity: Tau² = 0.20; Chi² = 3.54, df = 3 (P = 0.32); I² = 15%
Test for overall effect: Z = 0.75 (P = 0.45)

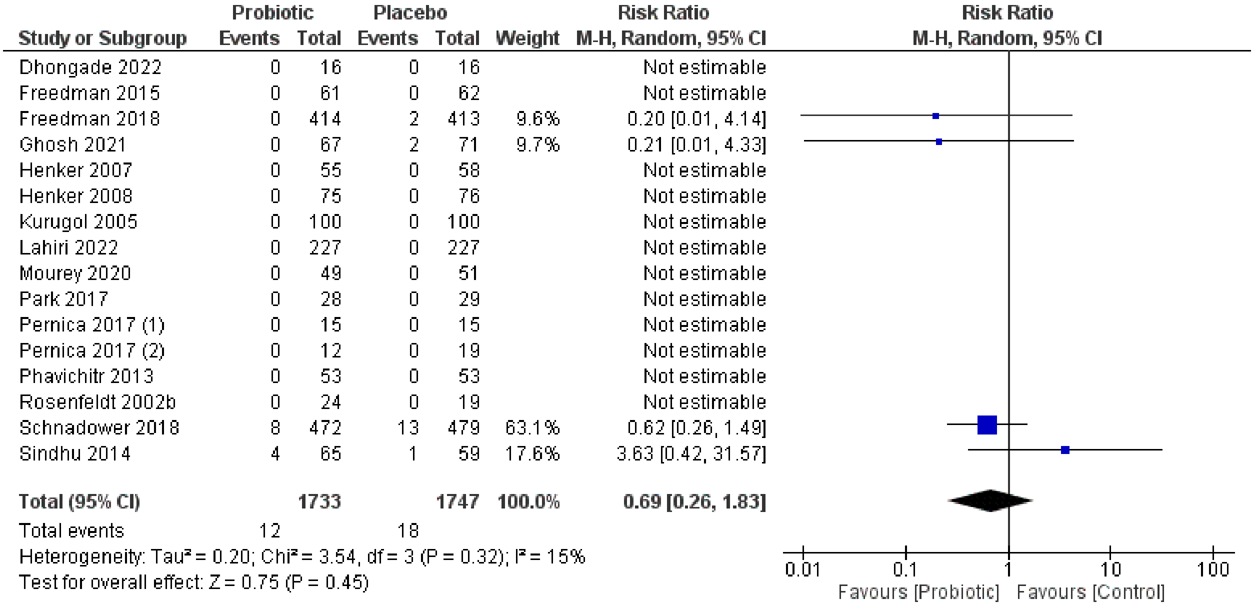

Footnotes
(1) Rapid test and treat
(2) Standard care

Figure S19

Forest plot for sensitivity analysis for serious adverse events based on ROB (WHO defined studies; n=6)

Study or Subgroup	Probiotic Events	Probiotic Total	Control Events	Control Total	Weight	Risk Ratio M-H, Random, 95% CI
Freedman 2015	0	61	0	62		Not estimable
Freedman 2018	0	414	2	413	14.1%	0.20 [0.01, 4.14]
Lahiri 2022	0	227	0	227		Not estimable
Pernica 2017 (1)	0	15	0	15		Not estimable
Pernica 2017 (2)	0	12	0	19		Not estimable
Schnadower 2018	8	472	13	479	61.9%	0.62 [0.26, 1.49]
Sindhu 2014	4	65	1	59	24.0%	3.63 [0.42, 31.57]
**Total (95% CI)**		**1266**		**1274**	**100.0%**	**0.81 [0.23, 2.81]**
Total events	12		16			

Heterogeneity: Tau² = 0.45; Chi² = 2.95, df = 2 (P = 0.23); I² = 32%
Test for overall effect: Z = 0.33 (P = 0.74)

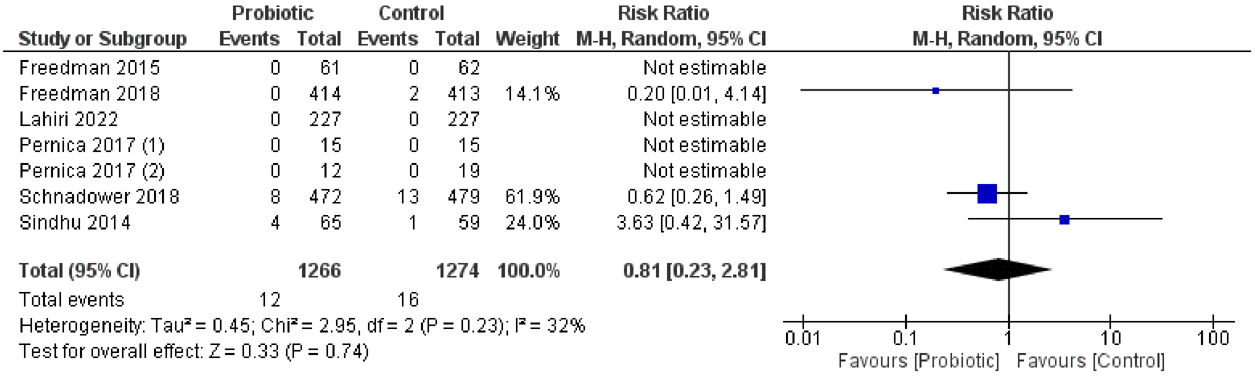

Footnotes
(1) Rapid test and treat
(2) Standard care

Figure S20

Forest plot for outcome: Mortality (n= 4)

Study or Subgroup	Probiotic Events	Probiotic Total	Placebo Events	Placebo Total	Weight	Risk Ratio M-H, Random, 95% CI
Basu 2009	0	392	1	196	30.2%	0.17 [0.01, 4.08]
Lahiri 2022	0	227	0	227		Not estimable
Pernica 2017 (1)	0	15	0	15		Not estimable
Pernica 2017 (2)	0	12	0	19		Not estimable
Pernica 2022 (3)	1	135	6	137	69.8%	0.17 [0.02, 1.39]
**Total (95% CI)**		**781**		**594**	**100.0%**	**0.17 [0.03, 0.98]**
Total events	1		7			

Heterogeneity: Tau² = 0.00; Chi² = 0.00, df = 1 (P = 0.99); I² = 0%
Test for overall effect: Z = 1.99 (P = 0.05)

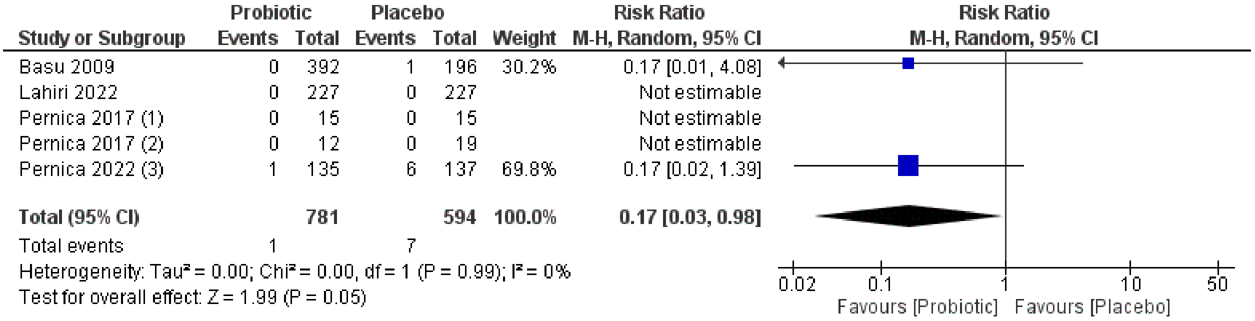

Footnotes
(1) Rapid Test and Treat
(2) Standard Care
(3) Considered overall mortality

Figure S21

Forest plot for outcome: Persistent diarrhea (n= 2)

Study or Subgroup	Probiotic Mean [Hours]	Probiotic SD [Hours]	Probiotic Total	Control Mean [Hours]	Control SD [Hours]	Control Total	Weight	Mean Difference IV, Random, 95% CI
Basu 2007a	127.2	50.4	117	220.8	67.2	118	86.0%	-93.60 [-108.78, -78.42]
Gaon 2003	90	33.42	60	204	100.8	29	14.0%	-114.00 [-151.65, -76.35]
**Total (95% CI)**			**177**			**147**	**100.0%**	**-96.45 [-110.53, -82.37]**

Heterogeneity: Tau² = 0.00; Chi² = 0.97, df = 1 (P = 0.32); I² = 0%
Test for overall effect: Z = 13.43 (P < 0.00001)

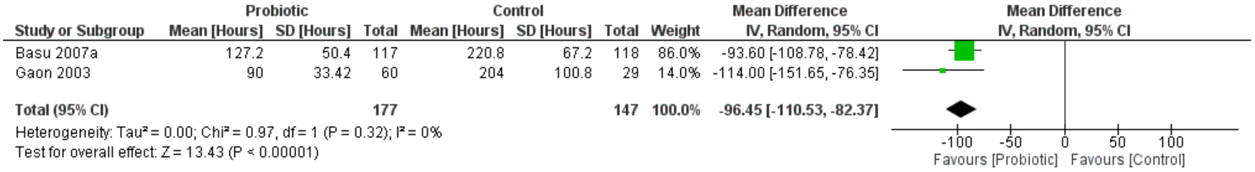

Figure S22

Comparison 1: Single vs. Multiple Strain, Outcome 1: Clinical Cure (WHO-defined studies; n=14)

Study or Subgroup	Probiotic Events	Probiotic Total	Control Events	Control Total	Weight	Risk Ratio M-H, Random, 95% CI
**6.1.1 Single strain**						
Correa 2011	66	95	40	91	5.6%	1.58 [1.21, 2.07]
Dinleyici 2015a	29	29	31	31	8.7%	1.00 [0.94, 1.07]
Dinleyici 2015b (1)	205	220	116	143	8.5%	1.15 [1.05, 1.25]
Eren 2010	23	28	21	27	5.6%	1.06 [0.81, 1.38]
Henker 2007	52	55	39	58	6.9%	1.41 [1.16, 1.70]
Henker 2008	74	75	54	76	7.6%	1.39 [1.20, 1.61]
Hong 2018	140	150	139	150	8.7%	1.01 [0.95, 1.07]
Khan 2012	210	210	210	210	9.0%	1.00 [0.99, 1.01]
Lai 2019 (2)	39	42	33	39	7.4%	1.10 [0.94, 1.29]
Ritchie 2010	25	33	24	31	5.5%	0.98 [0.75, 1.28]
Villarruel 2007	35	44	21	44	4.5%	1.67 [1.18, 2.35]
**Subtotal (95% CI)**		**981**		**900**	**78.0%**	**1.18 [0.95, 1.46]**
Total events	898		728			
Heterogeneity: Tau² = 0.12; Chi² = 740.00, df = 10 (P < 0.00001); I² = 99%						
Test for overall effect: Z = 1.51 (P = 0.13)						
**6.1.2 Multiple strain**						
Chen 2020	53	96	47	98	5.5%	1.15 [0.88, 1.51]
Cui 2016	35	43	42	43	7.5%	0.83 [0.72, 0.97]
Lahiri 2022	223	229	223	228	8.9%	1.00 [0.97, 1.02]
**Subtotal (95% CI)**		**368**		**369**	**22.0%**	**0.96 [0.84, 1.11]**
Total events	311		312			
Heterogeneity: Tau² = 0.01; Chi² = 6.54, df = 2 (P = 0.04); I² = 69%						
Test for overall effect: Z = 0.50 (P = 0.62)						
**Total (95% CI)**		**1349**		**1269**	**100.0%**	**1.12 [1.01, 1.24]**
Total events	1209		1040			
Heterogeneity: Tau² = 0.03; Chi² = 448.73, df = 13 (P < 0.00001); I² = 97%						
Test for overall effect: Z = 2.13 (P = 0.03)						
Test for subgroup differences: Chi² = 2.35, df = 1 (P = 0.12), I² = 57.5%						

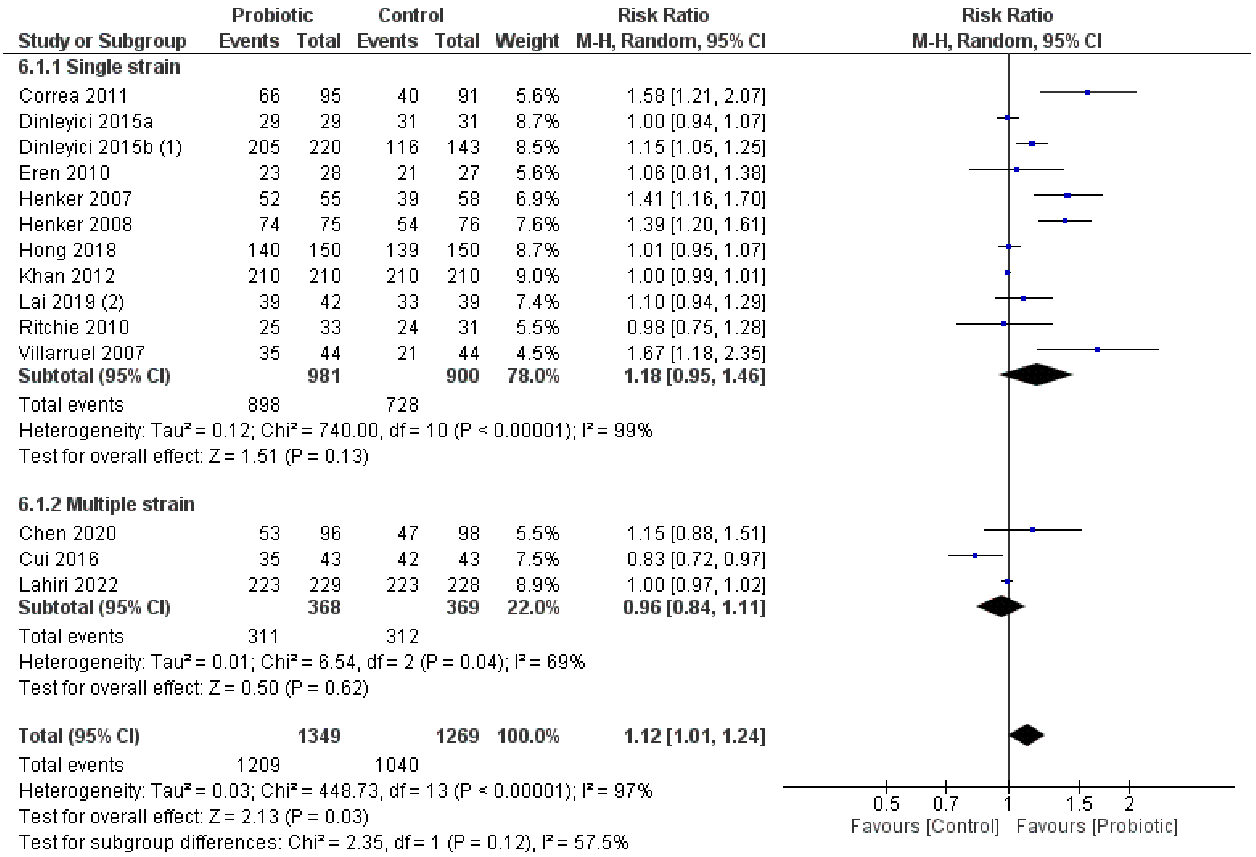

Footnotes
(1) Showed cumulative numbers for children from ED, hospitalized and outpatient settings.
(2) Observed on Day 14

Figure S23

Comparison 1: Single vs. Multiple Strain, Outcome 2: Clinical Deterioration (WHO-defined studies; n=10)

	Probiotic		Control			Risk Ratio
**Study or Subgroup**	**Events**	**Total**	**Events**	**Total**	**Weight**	**M-H, Random, 95% CI**
**6.7.2 Single Strain**						
Costa-Ribeiro 2003	8	61	6	63	10.6%	1.38 [0.51, 3.74]
Das 2016	2	30	5	28	4.8%	0.37 [0.08, 1.77]
Mourey 2020	3	49	8	51	7.0%	0.39 [0.11, 1.39]
Nixon 2012	2	67	1	71	2.1%	2.12 [0.20, 22.83]
Salazar-Lindo 2004	19	90	16	89	23.3%	1.17 [0.65, 2.13]
Schnadower 2018	15	468	15	475	18.5%	1.01 [0.50, 2.05]
**Subtotal (95% CI)**		**765**		**777**	**66.2%**	**0.99 [0.68, 1.45]**
Total events	49		51			
Heterogeneity: Tau² = 0.00; Chi² = 4.73, df = 5 (P = 0.45); I² = 0%						
Test for overall effect: Z = 0.04 (P = 0.97)						
**6.7.3 Multiple strain**						
Cui 2016	8	43	1	43	2.9%	8.00 [1.04, 61.24]
Freedman 2015	1	61	0	62	1.2%	3.05 [0.13, 73.40]
Freedman 2018	33	414	22	413	27.6%	1.50 [0.89, 2.52]
Lahiri 2022	1	227	2	227	2.1%	0.50 [0.05, 5.48]
**Subtotal (95% CI)**		**745**		**745**	**33.8%**	**1.75 [0.79, 3.88]**
Total events	43		25			
Heterogeneity: Tau² = 0.16; Chi² = 3.59, df = 3 (P = 0.31); I² = 16%						
Test for overall effect: Z = 1.37 (P = 0.17)						
**Total (95% CI)**		**1510**		**1522**	**100.0%**	**1.16 [0.81, 1.65]**
Total events	92		76			
Heterogeneity: Tau² = 0.05; Chi² = 10.58, df = 9 (P = 0.31); I² = 15%						
Test for overall effect: Z = 0.82 (P = 0.41)						
Test for subgroup differences: Chi² = 1.59, df = 1 (P = 0.21), I² = 37.0%						

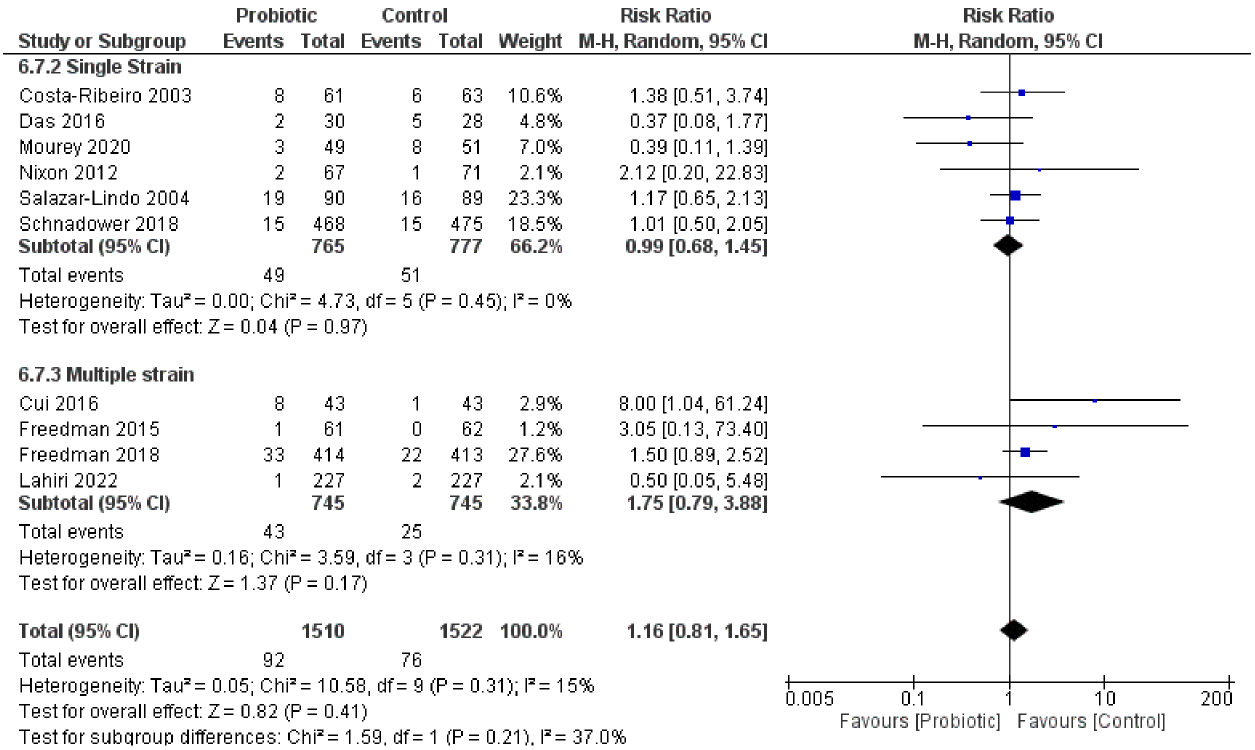

Figure S24

Comparison 1: Single vs. Multiple Strain, Outcome 3: Duration of Diarrhea (WHO-defined studies; n=30)

Study or Subgroup	Probiotic Mean [Hours]	Probiotic SD [Hours]	Probiotic Total	Control Mean [Hours]	Control SD [Hours]	Control Total	Weight	Mean Difference IV, Random, 95% CI
**6.6.1 Single strain**								
Aggarwal 2014	62	13.54	100	80	13.539	100	3.6%	-18.00 [-21.75, -14.25]
Basu 2007b	163.2	50.4	323	158.4	55.2	323	3.2%	4.80 [-3.35, 12.95]
Costa-Ribeiro 2003	38.27	3.78	61	39.09	4.6	63	3.8%	-0.82 [-2.30, 0.66]
Dalgic 2011 (1)	109.2	31.68	60	107.04	29.28	60	2.8%	2.16 [-8.76, 13.08]
Dalgic 2011 (2)	104.64	44.4	60	114	32.4	60	2.4%	-9.36 [-23.27, 4.55]
Dalgic 2011 (3)	114.72	35.04	60	128.4	43.2	60	2.4%	-13.68 [-27.75, 0.39]
Dalgic 2011 (4)	74.64	43.44	60	81.84	33.12	60	2.4%	-7.20 [-21.02, 6.62]
Das 2016	59.33	12.45	30	84	21.091	28	3.0%	-24.67 [-33.66, -15.68]
Dinleyici 2015a	60.4	24.5	29	74.3	15.3	31	2.9%	-13.90 [-24.32, -3.48]
Dinleyici 2015b (5)	83.1	36.3	148	114.8	35.4	72	2.9%	-31.70 [-41.75, -21.65]
Dinleyici 2015b (6)	60.4	23.1	25	78.4	20.9	26	2.6%	-18.00 [-30.11, -5.89]
Dinleyici 2015b (7)	58.9	15.3	47	88	19.1	45	3.3%	-29.10 [-36.19, -22.01]
El-Soud 2015	74.88	22.08	25	98.4	22.56	25	2.6%	-23.52 [-35.89, -11.15]
Eren 2010	106.8	59.04	28	129.12	75.36	27	0.8%	-22.32 [-58.18, 13.54]
Hong 2018	80.33	42.667	150	79.66	41.17	150	3.0%	0.67 [-8.82, 10.16]
Khan 2012	82.32	31.2	210	108	50.4	210	3.2%	-25.68 [-33.70, -17.66]
Khan 2021	144	34.08	100	144	38.16	100	2.9%	0.00 [-10.03, 10.03]
Maugo 2021	77.59	34.1	44	86.74	40.16	46	2.2%	-9.15 [-24.52, 6.22]
Misra 2009	70.56	31.2	105	78	26.4	105	3.2%	-7.44 [-15.26, 0.38]
Salazar-Lindo 2004	58.5	30.2	52	50.4	28	51	2.7%	8.10 [-3.14, 19.34]
Schnadower 2018	51.633	50.268	468	54.7	46.99	475	3.4%	-3.07 [-9.28, 3.15]
Sudarmo 2003	26.4	24	50	62.4	24	50	3.0%	-36.00 [-45.41, -26.59]
Sudha 2019	75.25	12.96	59	81.6	15.43	60	3.5%	-6.35 [-11.47, -1.23]
Villarruel 2007	112.8	46.56	44	147.84	76.8	44	1.2%	-35.04 [-61.58, -8.50]
**Subtotal (95% CI)**			**2338**			**2271**	**67.0%**	**-12.56 [-17.68, -7.43]**
Heterogeneity: Tau² = 130.99; Chi² = 270.83, df = 23 (P < 0.00001); I² = 92%								
Test for overall effect: Z = 4.80 (P < 0.00001)								
**6.6.2 Multiple strain**								
Agarwal 2002 (8)	49.2	21.816	50	57.6	21.6	25	2.9%	-8.40 [-18.80, 2.00]
Agarwal 2002 (9)	39.888	13.6536	50	50.4	17.28	25	3.2%	-10.51 [-18.27, -2.75]
Chen 2010	60.1	31.7	150	86.3	37.6	143	3.2%	-26.20 [-34.18, -18.22]
Chen 2020	121.4	13.7	96	143.9	19.8	98	3.5%	-22.50 [-27.28, -17.72]
Dhongade 2022	79.2	12.96	16	105.6	15.43	16	2.9%	-26.40 [-36.27, -16.53]
Freedman 2015	71.1	78.31	61	63.5	64.3	62	1.3%	7.60 [-17.74, 32.94]
Freedman 2018	55.533	57.654	414	59.33	61.07	413	3.2%	-3.80 [-11.89, 4.30]
Huang 2014	43.2	38.4	82	69.4	33.6	77	2.8%	-26.20 [-37.40, -15.00]
Mao 2008	67.0071	3.6664	141	68	4	71	3.8%	-0.99 [-2.10, 0.12]
Phavichitr 2013	103.92	58.896	53	120	36.576	53	1.9%	-16.08 [-34.74, 2.58]
Salazar-Lindo 2007	24.233	39.101	37	24.66	33.21	40	2.1%	-0.43 [-16.70, 15.84]
Teran 2009	57.1	25.4	25	74.6	26.6	25	2.3%	-17.50 [-31.92, -3.08]
**Subtotal (95% CI)**			**1175**			**1048**	**33.0%**	**-13.38 [-21.25, -5.50]**
Heterogeneity: Tau² = 157.95; Chi² = 157.82, df = 11 (P < 0.00001); I² = 93%								
Test for overall effect: Z = 3.33 (P = 0.0009)								
**Total (95% CI)**			**3513**			**3319**	**100.0%**	**-12.81 [-16.37, -9.24]**
Heterogeneity: Tau² = 87.50; Chi² = 440.13, df = 35 (P < 0.00001); I² = 92%								
Test for overall effect: Z = 7.04 (P < 0.00001)								
Test for subgroup differences: Chi² = 0.03, df = 1 (P = 0.86), I² = 0%								

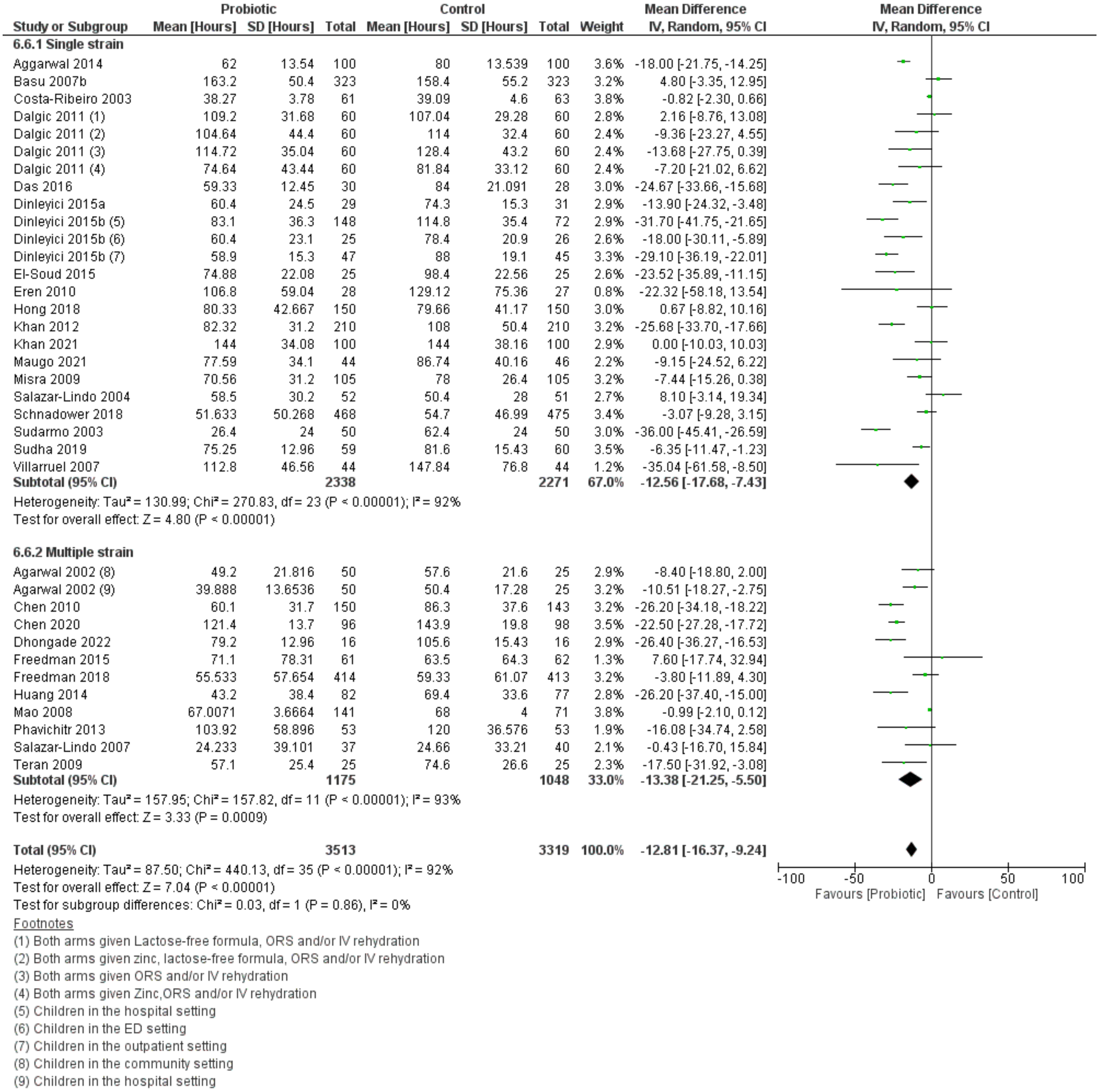

Footnotes
(1) Both arms given Lactose-free formula, ORS and/or IV rehydration
(2) Both arms given zinc, lactose-free formula, ORS and/or IV rehydration
(3) Both arms given ORS and/or IV rehydration
(4) Both arms given Zinc,ORS and/or IV rehydration
(5) Children in the hospital setting
(6) Children in the ED setting
(7) Children in the outpatient setting
(8) Children in the community setting
(9) Children in the hospital setting

Figure S25

Comparison 1: Single vs. Multiple Strain, Outcome 4: Mortality (n=4)

	Probiotic		Control			Risk Ratio
**Study or Subgroup**	**Events**	**Total**	**Events**	**Total**	**Weight**	**M-H, Random, 95% CI**
**6.5.1 Single strain**						
Basu 2009	0	392	1	196	30.2%	0.17 [0.01, 4.08]
Pernica 2017 (1)	0	15	0	15		Not estimable
Pernica 2017 (2)	0	12	0	19		Not estimable
Pernica 2022 (3)	1	135	6	137	69.8%	0.17 [0.02, 1.39]
**Subtotal (95% CI)**		**554**		**367**	**100.0%**	**0.17 [0.03, 0.98]**
Total events	1		7			
Heterogeneity: Tau² = 0.00; Chi² = 0.00, df = 1 (P = 0.99); I² = 0%						
Test for overall effect: Z = 1.99 (P = 0.05)						
**6.5.2 Multiple strain**						
Lahiri 2022	0	227	0	227		Not estimable
**Subtotal (95% CI)**		**227**		**227**		**Not estimable**
Total events	0		0			
Heterogeneity: Not applicable						
Test for overall effect: Not applicable						
**Total (95% CI)**		**781**		**594**	**100.0%**	**0.17 [0.03, 0.98]**
Total events	1		7			
Heterogeneity: Tau² = 0.00; Chi² = 0.00, df = 1 (P = 0.99); I² = 0%						
Test for overall effect: Z = 1.99 (P = 0.05)						
Test for subgroup differences: Not applicable						

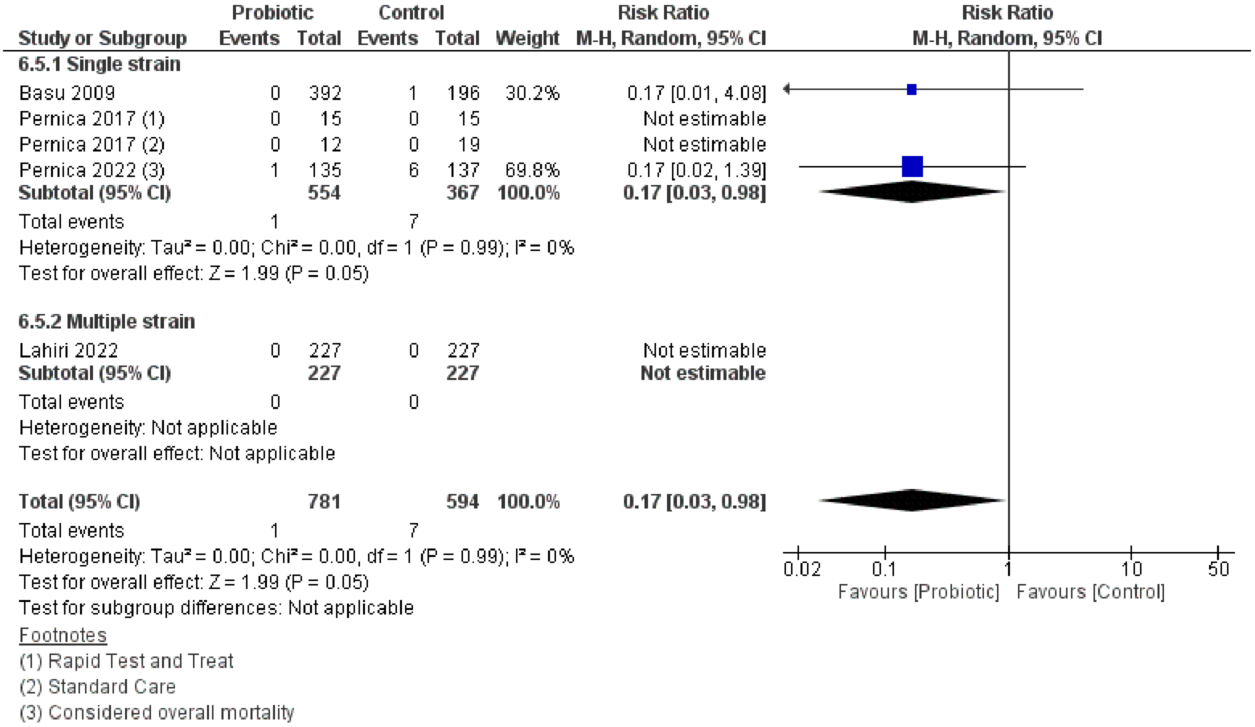

Footnotes
(1) Rapid Test and Treat
(2) Standard Care
(3) Considered overall mortality

Figure S26

Comparison 2: Probiotic Genus, Outcome 1: Clinical Cure (WHO-defined studies; n=12)

Study or Subgroup	Probiotic Events	Probiotic Total	Control Events	Control Total	Weight	Risk Ratio M-H, Random, 95% CI
**7.1.2 Sacchromyces**						
Correa 2011	66	95	40	91	6.6%	1.58 [1.21, 2.07]
Dinleyici 2015b (1)	205	220	116	143	9.6%	1.15 [1.05, 1.25]
Eren 2010	23	28	21	27	6.7%	1.06 [0.81, 1.38]
Khan 2012	210	210	210	210	10.1%	1.00 [0.99, 1.01]
Villarruel 2007	35	44	21	44	5.4%	1.67 [1.18, 2.35]
**Subtotal (95% CI)**		**597**		**515**	**38.4%**	**1.26 [0.64, 2.47]**
Total events	539		408			
Heterogeneity: Tau² = 0.58; Chi² = 750.57, df = 4 (P < 0.00001); I² = 99%						
Test for overall effect: Z = 0.67 (P = 0.51)						
**7.1.3 Bacillus**						
Lahiri 2022	223	229	223	228	10.0%	1.00 [0.97, 1.02]
**Subtotal (95% CI)**		**229**		**228**	**10.0%**	**1.00 [0.97, 1.02]**
Total events	223		223			
Heterogeneity: Not applicable						
Test for overall effect: Z = 0.30 (P = 0.77)						
**7.1.4 Lactobacillus**						
Dinleyici 2015a	29	29	31	31	9.8%	1.00 [0.94, 1.07]
Hong 2018	140	150	139	150	9.8%	1.01 [0.95, 1.07]
Lai 2019 (2)	39	42	33	39	8.6%	1.10 [0.94, 1.29]
Ritchie 2010	25	33	24	31	6.6%	0.98 [0.75, 1.28]
**Subtotal (95% CI)**		**254**		**251**	**34.8%**	**1.01 [0.97, 1.05]**
Total events	233		227			
Heterogeneity: Tau² = 0.00; Chi² = 1.32, df = 3 (P = 0.72); I² = 0%						
Test for overall effect: Z = 0.44 (P = 0.66)						
**7.1.5 Escherichia**						
Henker 2007	52	55	39	58	8.0%	1.41 [1.16, 1.70]
Henker 2008	74	75	54	76	8.7%	1.39 [1.20, 1.61]
**Subtotal (95% CI)**		**130**		**134**	**16.7%**	**1.40 [1.24, 1.57]**
Total events	126		93			
Heterogeneity: Tau² = 0.00; Chi² = 0.01, df = 1 (P = 0.92); I² = 0%						
Test for overall effect: Z = 5.63 (P < 0.00001)						
**Total (95% CI)**		**1210**		**1128**	**100.0%**	**1.15 [1.02, 1.29]**
Total events	1121		951			
Heterogeneity: Tau² = 0.04; Chi² = 506.47, df = 11 (P < 0.00001); I² = 98%						
Test for overall effect: Z = 2.29 (P = 0.02)						
Test for subgroup differences: Chi² = 31.12, df = 3 (P < 0.00001), I² = 90.4%						

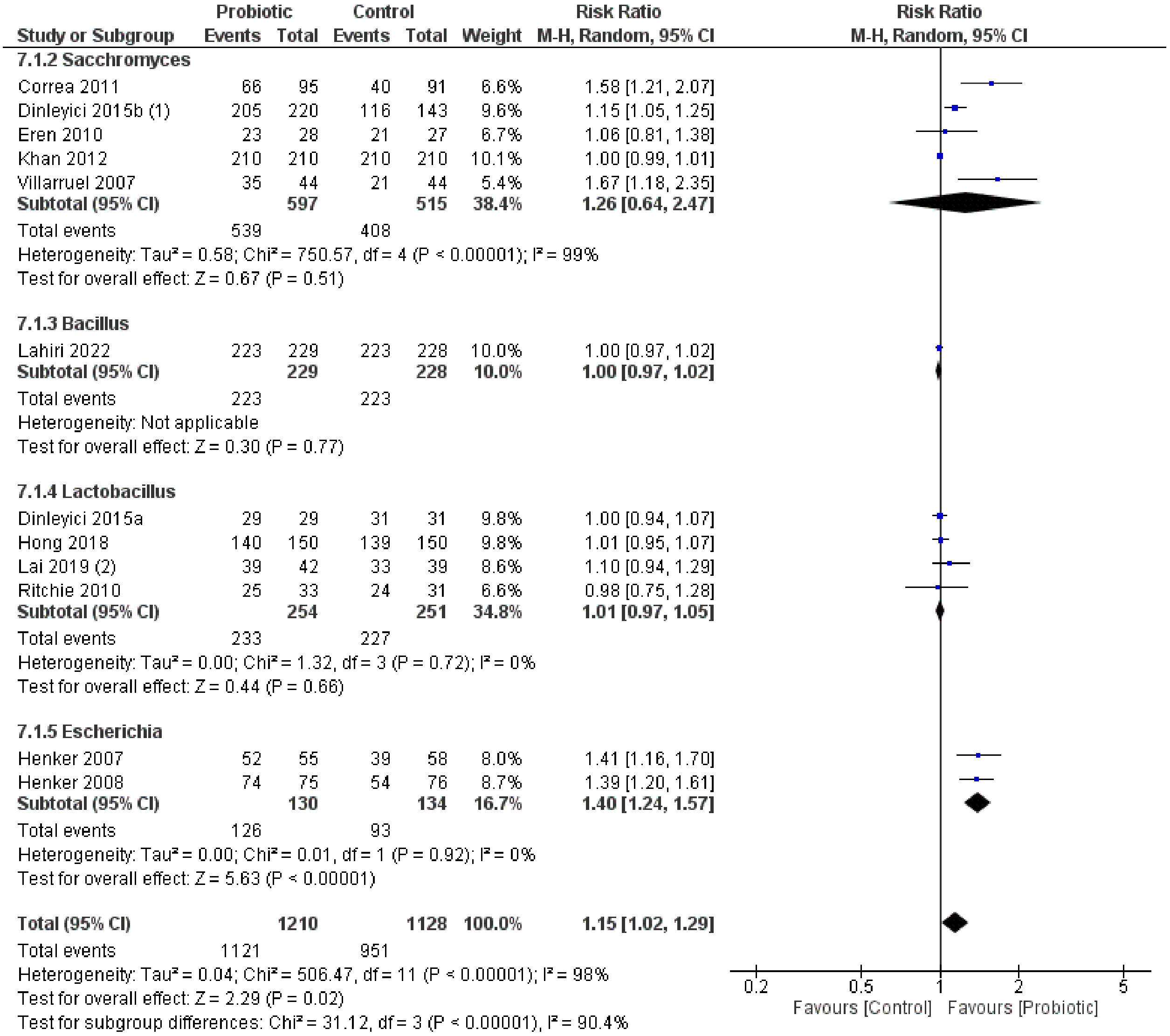

Footnotes
(1) Showed cumulative numbers for children from ED, hospitalized and outpatient settings
(2) Observed on Day 14

Figure S27

Comparison 2: Probiotic Genus, Outcome 2: Clinical Deterioration (WHO-defined studies; n=9)

Study or Subgroup	Probiotic Events	Probiotic Total	Control Events	Control Total	Weight	Risk Ratio M-H, Random, 95% CI
**7.7.2 Sacchromyces**						
Das 2016	2	30	5	28	3.8%	0.37 [0.08, 1.77]
Mourey 2020	3	49	8	51	5.7%	0.39 [0.11, 1.39]
**Subtotal (95% CI)**		**79**		**79**	**9.4%**	**0.38 [0.14, 1.02]**
Total events	5		13			
Heterogeneity: Tau² = 0.00; Chi² = 0.00, df = 1 (P = 0.97); I² = 0%						
Test for overall effect: Z = 1.91 (P = 0.06)						
**7.7.3 Bacillus**						
Lahiri 2022	1	227	2	227	1.6%	0.50 [0.05, 5.48]
**Subtotal (95% CI)**		**227**		**227**	**1.6%**	**0.50 [0.05, 5.48]**
Total events	1		2			
Heterogeneity: Not applicable						
Test for overall effect: Z = 0.57 (P = 0.57)						
**7.7.4 Lactobacillus**						
Costa-Ribeiro 2003	8	61	6	63	9.1%	1.38 [0.51, 3.74]
Freedman 2015	1	61	0	62	0.9%	3.05 [0.13, 73.40]
Freedman 2018	33	414	22	413	33.4%	1.50 [0.89, 2.52]
Nixon 2012	2	67	1	71	1.6%	2.12 [0.20, 22.83]
Salazar-Lindo 2004	19	90	16	89	25.5%	1.17 [0.65, 2.13]
Schnadower 2018	15	468	15	475	18.4%	1.01 [0.50, 2.05]
**Subtotal (95% CI)**		**1161**		**1173**	**89.0%**	**1.29 [0.94, 1.78]**
Total events	78		60			
Heterogeneity: Tau² = 0.00; Chi² = 1.32, df = 5 (P = 0.93); I² = 0%						
Test for overall effect: Z = 1.58 (P = 0.11)						
**7.7.5 Escherichia**						
**Subtotal (95% CI)**		**0**		**0**		**Not estimable**
Total events	0		0			
Heterogeneity: Not applicable						
Test for overall effect: Not applicable						
**Total (95% CI)**		**1467**		**1479**	**100.0%**	**1.14 [0.84, 1.54]**
Total events	84		75			
Heterogeneity: Tau² = 0.00; Chi² = 7.11, df = 8 (P = 0.53); I² = 0%						
Test for overall effect: Z = 0.83 (P = 0.40)						
Test for subgroup differences: Chi² = 5.78, df = 2 (P = 0.06), I² = 65.4%						

Risk Ratio
M-H, Random, 95% CI

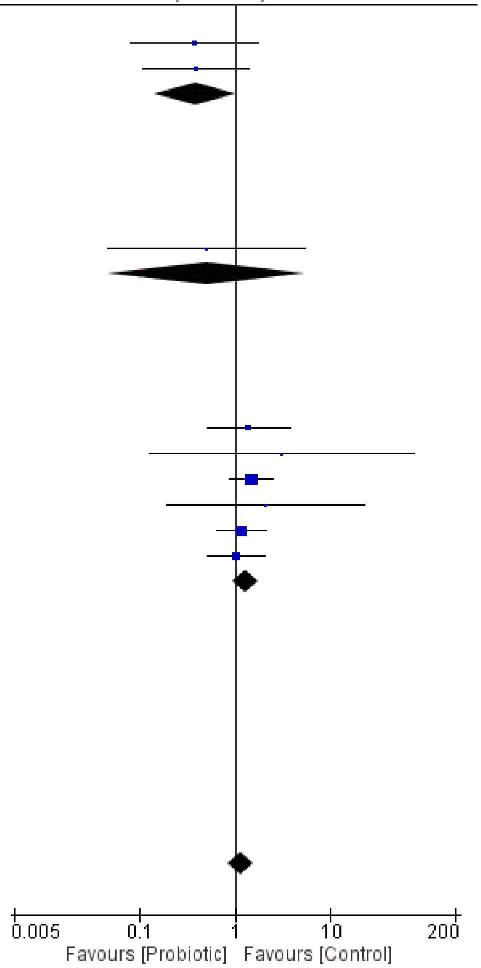

Figure S28

Comparison 2: Probiotic genus, Outcome 3: Duration of diarrhea (WHO-defined studies; n=23)

	Probiotic			Control				Mean Difference
Study or Subgroup	Mean [Hours]	SD [Hours]	Total	Mean [Hours]	SD [Hours]	Total	Weight	IV, Random, 95% CI
**7.2.3 Sacchromyces**								
Dalgic 2011 (1)	114.72	35.04	60	128.4	43.2	60	3.3%	-13.68 [-27.75, 0.39]
Dalgic 2011 (2)	74.64	43.44	60	81.84	33.12	60	3.3%	-7.20 [-21.02, 6.62]
Dalgic 2011 (3)	109.2	31.68	60	107.04	29.28	60	3.7%	2.16 [-8.76, 13.08]
Dalgic 2011 (4)	104.64	44.4	60	114	32.4	60	3.3%	-9.36 [-23.27, 4.55]
Das 2016	59.33	12.45	30	84	21.091	28	3.9%	-24.67 [-33.66, -15.68]
Dinleyici 2015b (5)	83.1	36.3	148	114.8	35.4	72	3.8%	-31.70 [-41.75, -21.65]
Dinleyici 2015b (6)	60.4	23.1	25	78.4	20.9	26	3.5%	-18.00 [-30.11, -5.89]
Dinleyici 2015b (7)	58.9	15.3	47	88	19.1	45	4.1%	-29.10 [-36.19, -22.01]
Eren 2010	106.8	59.04	28	129.12	75.36	27	1.3%	-22.32 [-58.18, 13.54]
Khan 2012	82.32	31.2	210	108	50.4	210	4.0%	-25.68 [-33.70, -17.66]
Khan 2021	144	34.08	100	144	38.16	100	3.8%	0.00 [-10.03, 10.03]
Villarruel 2007	112.8	46.56	44	147.84	76.8	44	1.9%	-35.04 [-61.58, -8.50]
**Subtotal (95% CI)**			**872**			**792**	**39.9%**	**-17.30 [-24.70, -9.90]**
Heterogeneity: Tau² = 123.23; Chi² = 53.00, df = 11 (P < 0.00001); I² = 79%								
Test for overall effect: Z = 4.58 (P < 0.00001)								
**7.2.4 Bacillus**								
Dhongade 2022	79.2	12.96	16	105.6	15.43	16	3.8%	-26.40 [-36.27, -16.53]
Maugo 2021	77.59	34.1	44	86.74	40.16	46	3.1%	-9.15 [-24.52, 6.22]
Sudha 2019	75.25	12.96	59	81.6	15.43	60	4.3%	-6.35 [-11.47, -1.23]
**Subtotal (95% CI)**			**119**			**122**	**11.2%**	**-13.91 [-27.72, -0.11]**
Heterogeneity: Tau² = 120.84; Chi² = 12.52, df = 2 (P = 0.002); I² = 84%								
Test for overall effect: Z = 1.98 (P = 0.05)								
**7.2.5 Lactobacillus**								
Aggarwal 2014	62	13.54	100	80	13.539	100	4.4%	-18.00 [-21.75, -14.25]
Basu 2007b	163.2	50.4	323	158.4	55.2	323	4.0%	4.80 [-3.35, 12.95]
Costa-Ribeiro 2003	38.27	3.78	61	39.09	4.6	63	4.6%	-0.82 [-2.30, 0.66]
Dinleyici 2015a	60.4	24.5	29	74.3	15.3	31	3.7%	-13.90 [-24.32, -3.48]
Freedman 2015	71.1	78.31	61	63.5	64.3	62	2.0%	7.60 [-17.74, 32.94]
Freedman 2018	55.533	57.654	414	59.33	61.07	413	4.0%	-3.80 [-11.89, 4.30]
Hong 2018	80.33	42.667	150	79.66	41.17	150	3.9%	0.67 [-8.82, 10.16]
Misra 2009	70.56	31.2	105	78	26.4	105	4.1%	-7.44 [-15.26, 0.38]
Salazar-Lindo 2004	58.5	30.2	52	50.4	28	51	3.6%	8.10 [-3.14, 19.34]
Salazar-Lindo 2007	24.233	39.101	37	24.66	33.21	40	3.0%	-0.43 [-16.70, 15.84]
Schnadower 2018	51.633	50.268	468	54.7	46.99	475	4.2%	-3.07 [-9.28, 3.15]
**Subtotal (95% CI)**			**1800**			**1813**	**41.5%**	**-3.43 [-9.01, 2.15]**
Heterogeneity: Tau² = 65.30; Chi² = 83.79, df = 10 (P < 0.00001); I² = 88%								
Test for overall effect: Z = 1.20 (P = 0.23)								
**7.2.6 Bifidobacterium**								
El-Soud 2015	74.88	22.08	25	98.4	22.56	25	3.5%	-23.52 [-35.89, -11.15]
Sudarmo 2003	26.4	24	50	62.4	24	50	3.9%	-36.00 [-45.41, -26.59]
**Subtotal (95% CI)**			**75**			**75**	**7.4%**	**-30.43 [-42.59, -18.28]**
Heterogeneity: Tau² = 46.43; Chi² = 2.48, df = 1 (P = 0.12); I² = 60%								
Test for overall effect: Z = 4.91 (P < 0.00001)								
**Total (95% CI)**			**2866**			**2802**	**100.0%**	**-11.97 [-16.70, -7.23]**
Heterogeneity: Tau² = 127.80; Chi² = 289.03, df = 27 (P < 0.00001); I² = 91%								
Test for overall effect: Z = 4.95 (P < 0.00001)								
Test for subgroup differences: Chi² = 19.83, df = 3 (P = 0.0002), I² = 84.9%								

Mean Difference
IV, Random, 95% CI

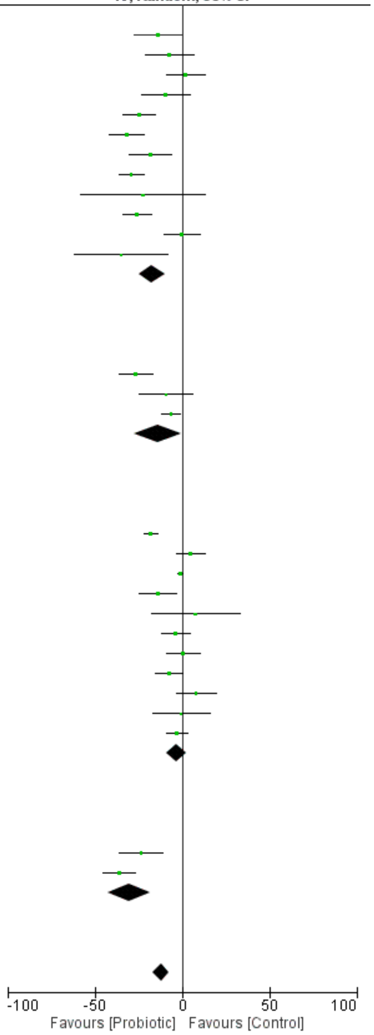

Footnotes
(1) Both arms given ORS and/or IV rehydration
(2) Both arms given Zinc,ORS and/or IV rehydration
(3) Both arms given Lactose-free formula, ORS and/or IV rehydration
(4) Both arms given zinc, lactose-free formula, ORS and/or IV rehydration
(5) Children in the hospital setting
(6) Children in the ED setting
(7) Children in the outpatient setting

Figure S29

Comparison 2: Probiotic genus, Outcome 4: Mortality (n=4)

Study or Subgroup	Probiotic Events	Probiotic Total	Control Events	Control Total	Weight	Risk Ratio M-H, Random, 95% CI
**7.5.2 Lactobacillus**						
Basu 2009	0	392	1	196	30.2%	0.17 [0.01, 4.08]
Pernica 2017 (1)	0	12	0	19		Not estimable
Pernica 2017 (2)	0	15	0	15		Not estimable
Pernica 2022 (3)	1	135	6	137	69.8%	0.17 [0.02, 1.39]
**Subtotal (95% CI)**		**554**		**367**	**100.0%**	**0.17 [0.03, 0.98]**
Total events	1		7			
Heterogeneity: Tau² = 0.00; Chi² = 0.00, df = 1 (P = 0.99); I² = 0%						
Test for overall effect: Z = 1.99 (P = 0.05)						
**7.5.3 Bacillus**						
Lahiri 2022	0	227	0	227		Not estimable
**Subtotal (95% CI)**		**227**		**227**		**Not estimable**
Total events	0		0			
Heterogeneity: Not applicable						
Test for overall effect: Not applicable						
**Total (95% CI)**		**781**		**594**	**100.0%**	**0.17 [0.03, 0.98]**
Total events	1		7			
Heterogeneity: Tau² = 0.00; Chi² = 0.00, df = 1 (P = 0.99); I² = 0%						
Test for overall effect: Z = 1.99 (P = 0.05)						
Test for subgroup differences: Not applicable						

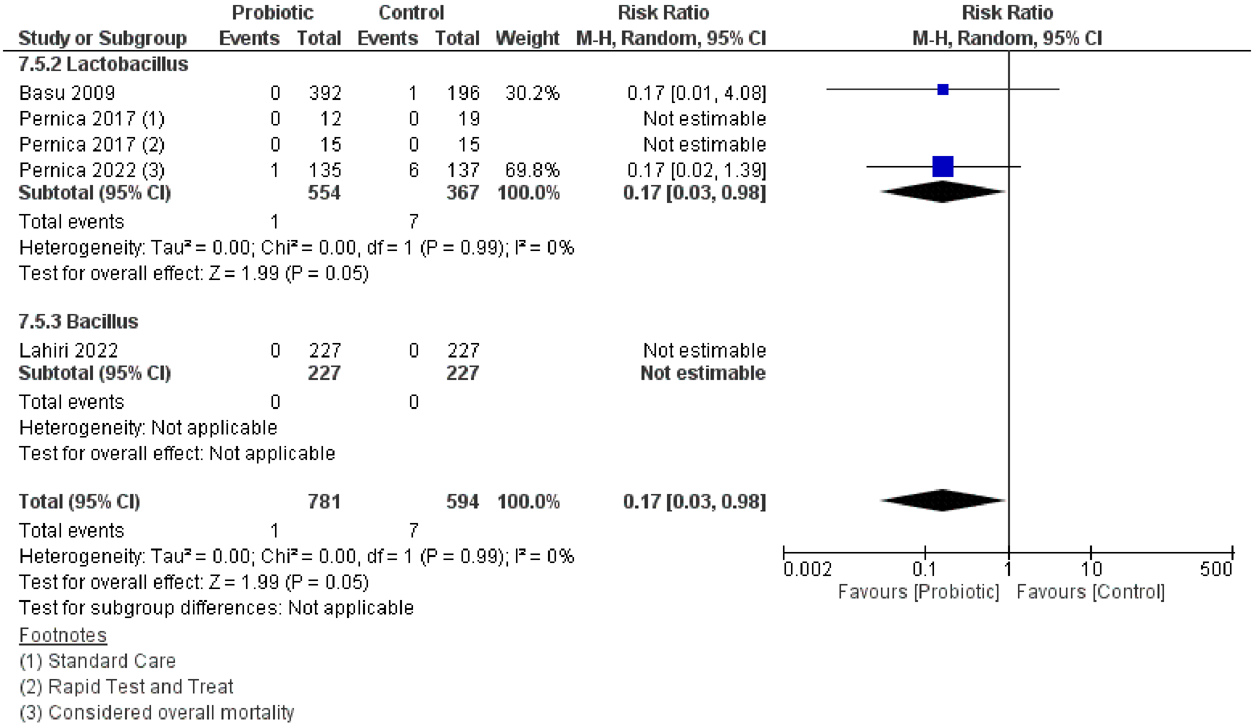

Footnotes
(1) Standard Care
(2) Rapid Test and Treat
(3) Considered overall mortality

Figure S30

Comparison 3: Probiotic strains, Outcome 1: Clinical cure (WHO-defined studies; n=11)

Study or Subgroup	Probiotic Events	Probiotic Total	Control Events	Control Total	Weight	Risk Ratio M-H, Random, 95% CI
**8.1.1 Sacchromyces boulardii**						
Correa 2011	66	95	40	91	8.5%	1.58 [1.21, 2.07]
Dinleyici 2015b (1)	205	220	116	143	9.6%	1.15 [1.05, 1.25]
Eren 2010	23	28	21	27	8.5%	1.06 [0.81, 1.38]
Khan 2012	210	210	210	210	9.8%	1.00 [0.99, 1.01]
Villarruel 2007	35	44	21	44	7.8%	1.67 [1.18, 2.35]
**Subtotal (95% CI)**		**597**		**515**	**44.3%**	**1.26 [0.64, 2.47]**
Total events	539		408			
Heterogeneity: Tau² = 0.58; Chi² = 750.57, df = 4 (P < 0.00001); I² = 99%						
Test for overall effect: Z = 0.67 (P = 0.51)						
**8.1.2 Lactobacillus acidophilus**						
Hong 2018	140	150	139	150	9.7%	1.01 [0.95, 1.07]
**Subtotal (95% CI)**		**150**		**150**	**9.7%**	**1.01 [0.95, 1.07]**
Total events	140		139			
Heterogeneity: Not applicable						
Test for overall effect: Z = 0.23 (P = 0.82)						
**8.1.3 Lactobacillus rhamnosus**						
Lai 2019 (2)	39	42	33	39	9.3%	1.10 [0.94, 1.29]
Ritchie 2010	25	33	24	31	8.5%	0.98 [0.75, 1.28]
**Subtotal (95% CI)**		**75**		**70**	**17.8%**	**1.07 [0.93, 1.22]**
Total events	64		57			
Heterogeneity: Tau² = 0.00; Chi² = 0.58, df = 1 (P = 0.45); I² = 0%						
Test for overall effect: Z = 0.92 (P = 0.36)						
**8.1.5 Lactobacillus reuteri**						
Dinleyici 2015a	29	29	31	31	9.7%	1.00 [0.94, 1.07]
**Subtotal (95% CI)**		**29**		**31**	**9.7%**	**1.00 [0.94, 1.07]**
Total events	29		31			
Heterogeneity: Not applicable						
Test for overall effect: Z = 0.00 (P = 1.00)						
**8.1.6 Escherichia coli**						
Henker 2007	52	55	39	58	9.1%	1.41 [1.16, 1.70]
Henker 2008	74	75	54	76	9.4%	1.39 [1.20, 1.61]
**Subtotal (95% CI)**		**130**		**134**	**18.5%**	**1.40 [1.24, 1.57]**
Total events	126		93			
Heterogeneity: Tau² = 0.00; Chi² = 0.01, df = 1 (P = 0.92); I² = 0%						
Test for overall effect: Z = 5.63 (P < 0.00001)						
**Total (95% CI)**		**981**		**900**	**100.0%**	**1.18 [0.95, 1.46]**
Total events	898		728			
Heterogeneity: Tau² = 0.12; Chi² = 740.00, df = 10 (P < 0.00001); I² = 99%						
Test for overall effect: Z = 1.51 (P = 0.13)						
Test for subgroup differences: Chi² = 27.41, df = 4 (P < 0.0001), I² = 85.4%						

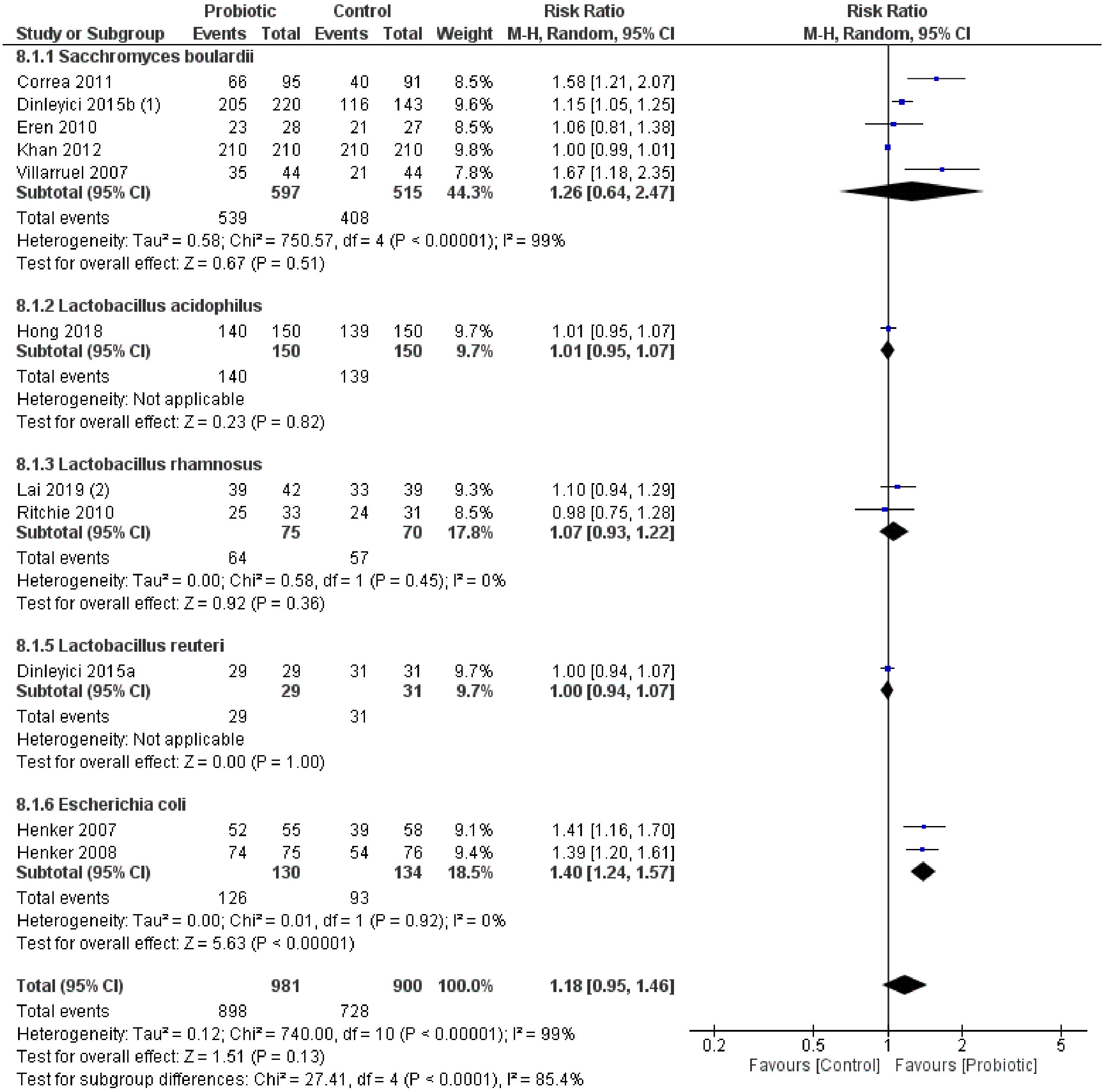

Footnotes
(1) Showed cumulative numbers for children from ED, hospitalized and outpatient settings
(2) Observed on Day 14

Figure S31

Comparison 3: Probiotic strains, Outcome 2: Clinical deterioration (WHO-defined studies; n=6)

Study or Subgroup	Probiotic Events	Probiotic Total	Control Events	Control Total	Weight	Risk Ratio M-H, Random, 95% CI
**8.7.2 Sacchromyces boulardii**						
Das 2016	2	30	5	28	5.9%	0.37 [0.08, 1.77]
Mourey 2020	3	49	8	51	8.8%	0.39 [0.11, 1.39]
**Subtotal (95% CI)**		**79**		**79**	**14.7%**	**0.38 [0.14, 1.02]**
Total events	5		13			
Heterogeneity: Tau² = 0.00; Chi² = 0.00, df = 1 (P = 0.97); I² = 0%						
Test for overall effect: Z = 1.91 (P = 0.06)						
**8.7.3 Lactobacillus acidophillus**						
**Subtotal (95% CI)**		**0**		**0**		**Not estimable**
Total events	0		0			
Heterogeneity: Not applicable						
Test for overall effect: Not applicable						
**8.7.4 Lactobacillus rhamnosus**						
Costa-Ribeiro 2003	8	61	6	63	14.3%	1.38 [0.51, 3.74]
Nixon 2012	2	67	1	71	2.5%	2.12 [0.20, 22.83]
Salazar-Lindo 2004	19	90	16	89	39.9%	1.17 [0.65, 2.13]
Schnadower 2018	15	468	15	475	28.7%	1.01 [0.50, 2.05]
**Subtotal (95% CI)**		**686**		**698**	**85.3%**	**1.17 [0.78, 1.76]**
Total events	44		38			
Heterogeneity: Tau² = 0.00; Chi² = 0.50, df = 3 (P = 0.92); I² = 0%						
Test for overall effect: Z = 0.75 (P = 0.45)						
**Total (95% CI)**		**765**		**777**	**100.0%**	**0.99 [0.68, 1.45]**
Total events	49		51			
Heterogeneity: Tau² = 0.00; Chi² = 4.73, df = 5 (P = 0.45); I² = 0%						
Test for overall effect: Z = 0.04 (P = 0.97)						
Test for subgroup differences: Chi² = 4.21, df = 1 (P = 0.04), I² = 76.2%						

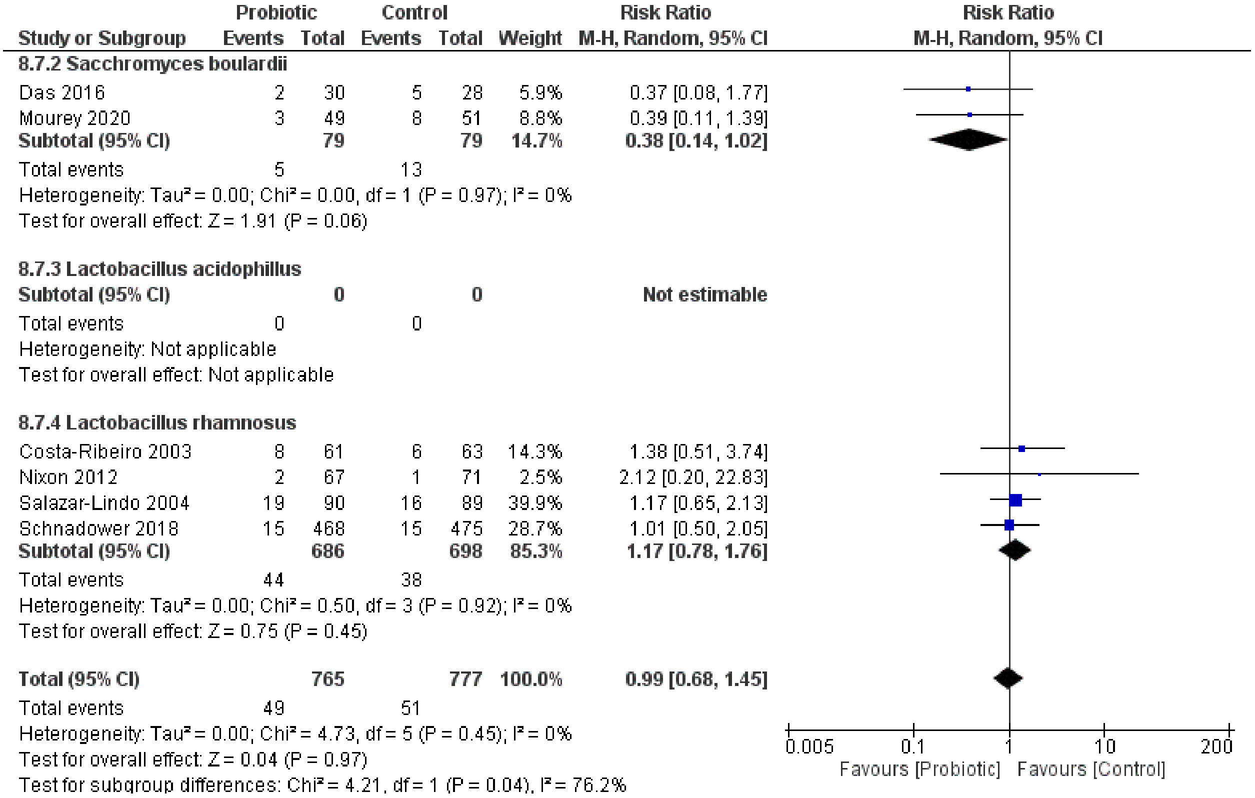

Figure S32

Comparison 3: Probiotic strains, Outcome 3: Duration of diarrhea (WHO-defined studies; n=19)

	Probiotic			Control				Mean Difference
Study or Subgroup	Mean [Hours]	SD [Hours]	Total	Mean [Hours]	SD [Hours]	Total	Weight	IV, Random, 95% CI
**8.2.1 Sacchromyces boulardii**								
Dalgic 2011 (1)	114.72	35.04	60	128.4	43.2	60	3.7%	-13.68 [-27.75, 0.39]
Dalgic 2011 (2)	74.64	43.44	60	81.84	33.12	60	3.8%	-7.20 [-21.02, 6.62]
Dalgic 2011 (3)	109.2	31.68	60	107.04	29.28	60	4.2%	2.16 [-8.76, 13.08]
Dalgic 2011 (4)	104.64	44.4	60	114	32.4	60	3.8%	-9.36 [-23.27, 4.55]
Das 2016	59.33	12.45	30	84	21.091	28	4.5%	-24.67 [-33.66, -15.68]
Dinleyici 2015b (5)	83.1	36.3	148	114.8	35.4	72	4.3%	-31.70 [-41.75, -21.65]
Dinleyici 2015b (6)	60.4	23.1	25	78.4	20.9	26	4.0%	-18.00 [-30.11, -5.89]
Dinleyici 2015b (7)	58.9	15.3	47	88	19.1	45	4.7%	-29.10 [-36.19, -22.01]
Eren 2010	106.8	59.04	28	129.12	75.36	27	1.5%	-22.32 [-58.18, 13.54]
Khan 2012	82.32	31.2	210	108	50.4	210	4.6%	-25.68 [-33.70, -17.66]
Khan 2021	144	34.08	100	144	38.16	100	4.3%	0.00 [-10.03, 10.03]
Villarruel 2007	112.8	46.56	44	147.84	76.8	44	2.2%	-35.04 [-61.58, -8.50]
**Subtotal (95% CI)**			**872**			**792**	**45.8%**	**-17.30 [-24.70, -9.90]**
Heterogeneity: Tau² = 123.23; Chi² = 53.00, df = 11 (P < 0.00001); I² = 79%								
Test for overall effect: Z = 4.58 (P < 0.00001)								
**8.2.2 Bacillus clausii**								
Maugo 2021	77.59	34.1	44	86.74	40.16	46	3.6%	-9.15 [-24.52, 6.22]
Sudha 2019	75.25	12.96	59	81.6	15.43	60	5.0%	-6.35 [-11.47, -1.23]
**Subtotal (95% CI)**			**103**			**106**	**8.5%**	**-6.63 [-11.48, -1.77]**
Heterogeneity: Tau² = 0.00; Chi² = 0.11, df = 1 (P = 0.73); I² = 0%								
Test for overall effect: Z = 2.68 (P = 0.007)								
**8.2.3 Lactobacillus acidophilus**								
Hong 2018	80.33	42.667	150	79.66	41.17	150	4.4%	0.67 [-8.82, 10.16]
**Subtotal (95% CI)**			**150**			**150**	**4.4%**	**0.67 [-8.82, 10.16]**
Heterogeneity: Not applicable								
Test for overall effect: Z = 0.14 (P = 0.89)								
**8.2.5 Lactobacillus rhamnosus**								
Aggarwal 2014	62	13.54	100	80	13.539	100	5.1%	-18.00 [-21.75, -14.25]
Basu 2007b	163.2	50.4	323	158.4	55.2	323	4.6%	4.80 [-3.35, 12.95]
Costa-Ribeiro 2003	38.27	3.78	61	39.09	4.6	63	5.2%	-0.82 [-2.30, 0.66]
Misra 2009	70.56	31.2	105	78	26.4	105	4.7%	-7.44 [-15.26, 0.38]
Salazar-Lindo 2004	58.5	30.2	52	50.4	28	51	4.2%	8.10 [-3.14, 19.34]
Schnadower 2018	51.633	50.268	468	54.7	46.99	475	4.8%	-3.07 [-9.28, 3.15]
**Subtotal (95% CI)**			**1109**			**1117**	**28.6%**	**-3.30 [-11.10, 4.49]**
Heterogeneity: Tau² = 82.62; Chi² = 78.18, df = 5 (P < 0.00001); I² = 94%								
Test for overall effect: Z = 0.83 (P = 0.41)								
**8.2.6 Lactobacillus reuteri**								
Dinleyici 2015a	60.4	24.5	29	74.3	15.3	31	4.3%	-13.90 [-24.32, -3.48]
**Subtotal (95% CI)**			**29**			**31**	**4.3%**	**-13.90 [-24.32, -3.48]**
Heterogeneity: Not applicable								
Test for overall effect: Z = 2.62 (P = 0.009)								
**8.2.7 Bifidobacterium bifidum**								
Sudarmo 2003	26.4	24	50	62.4	24	50	4.4%	-36.00 [-45.41, -26.59]
**Subtotal (95% CI)**			**50**			**50**	**4.4%**	**-36.00 [-45.41, -26.59]**
Heterogeneity: Not applicable								
Test for overall effect: Z = 7.50 (P < 0.00001)								
**8.2.8 Bifidobacterium lactis**								
El-Soud 2015	74.88	22.08	25	98.4	22.56	25	4.0%	-23.52 [-35.89, -11.15]
**Subtotal (95% CI)**			**25**			**25**	**4.0%**	**-23.52 [-35.89, -11.15]**
Heterogeneity: Not applicable								
Test for overall effect: Z = 3.73 (P = 0.0002)								
**Total (95% CI)**			**2338**			**2271**	**100.0%**	**-12.56 [-17.68, -7.43]**
Heterogeneity: Tau² = 130.99; Chi² = 270.83, df = 23 (P < 0.00001); I² = 92%								
Test for overall effect: Z = 4.80 (P < 0.00001)								
Test for subgroup differences: Chi² = 46.67, df = 6 (P < 0.00001), I² = 87.1%								

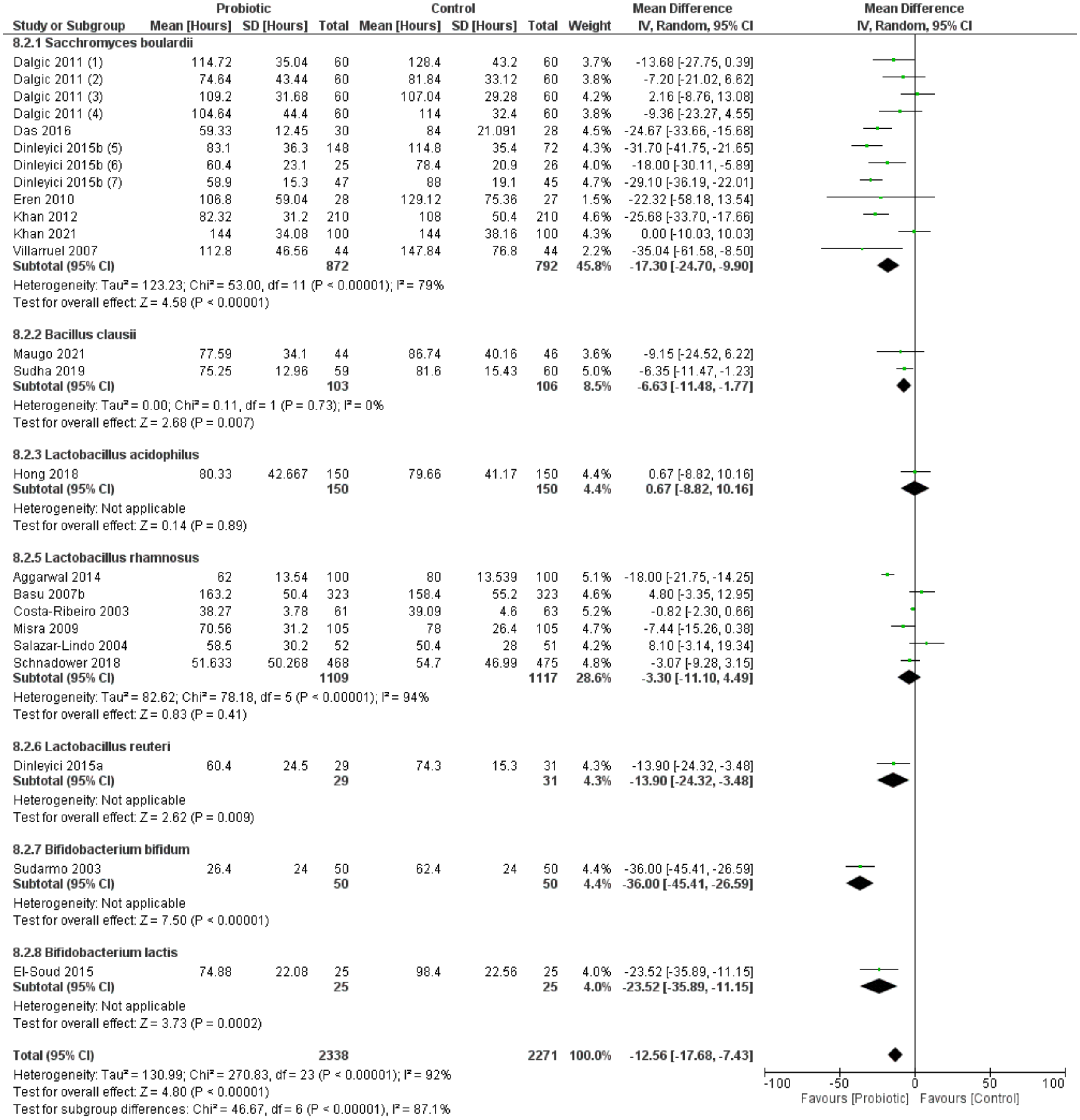

Footnotes
(1) Both arms given ORS and/or IV rehydration
(2) Both arms given Zinc,ORS and/or IV rehydration
(3) Both arms given Lactose-free formula, ORS and/or IV rehydration
(4) Both arms given zinc, lactose-free formula, ORS and/or IV rehydration
(5) Children in the hospital setting
(6) Children in the ED setting
(7) Children in the outpatient setting

Figure S33

Comparison 3: Probiotic strains, Outcome 4: Mortality (n=3)

	Probiotic		Placebo			Risk Ratio
**Study or Subgroup**	**Events**	**Total**	**Events**	**Total**	**Weight**	**M-H, Random, 95% CI**
**26.5.1 Lactobacillus reuteri**						
Pernica 2017 (1)	0	15	0	15		Not estimable
Pernica 2017 (2)	0	12	0	19		Not estimable
Pernica 2022 (3)	1	135	6	137	69.8%	0.17 [0.02, 1.39]
**Subtotal (95% CI)**		**162**		**171**	**69.8%**	**0.17 [0.02, 1.39]**
Total events	1		6			
Heterogeneity: Not applicable						
Test for overall effect: Z = 1.66 (P = 0.10)						
**26.5.2 Lactobacillus rhamnosus**						
Basu 2009	0	392	1	196	30.2%	0.17 [0.01, 4.08]
**Subtotal (95% CI)**		**392**		**196**	**30.2%**	**0.17 [0.01, 4.08]**
Total events	0		1			
Heterogeneity: Not applicable						
Test for overall effect: Z = 1.10 (P = 0.27)						
**Total (95% CI)**		**554**		**367**	**100.0%**	**0.17 [0.03, 0.98]**
Total events	1		7			
Heterogeneity: Tau² = 0.00; Chi² = 0.00, df = 1 (P = 0.99); I² = 0%						
Test for overall effect: Z = 1.99 (P = 0.05)						
Test for subgroup differences: Chi² = 0.00, df = 1 (P = 1.00), I² = 0%						

Risk Ratio
M-H, Random, 95% CI

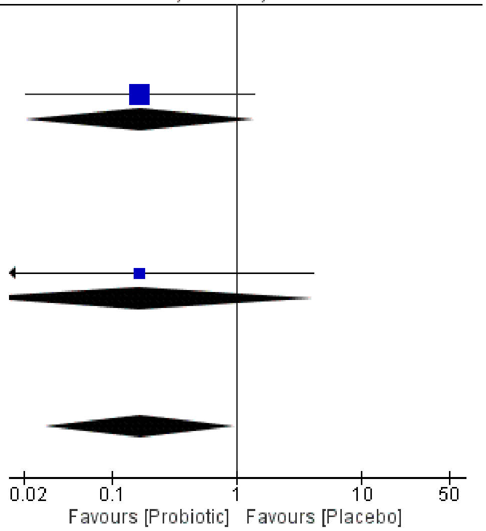

Footnotes
(1) Rapid Test and Treat
(2) Standard Care
(3) Considered overall mortality

Figure S34

Comparison 4: Source of funding, Outcome 1: Clinical cure (WHO-defined studies; n=8)

Study or Subgroup	Probiotic Events	Probiotic Total	Control Events	Control Total	Weight	Risk Ratio M-H, Random, 95% CI
**15.1.2 Trial supported by Pharmaceutical/Industry**						
Henker 2007	52	55	39	58	10.3%	1.41 [1.16, 1.70]
Lahiri 2022	223	229	223	228	16.6%	1.00 [0.97, 1.02]
**Subtotal (95% CI)**		**284**		**286**	**26.9%**	**1.18 [0.73, 1.89]**
Total events	275		262			
Heterogeneity: Tau² = 0.11; Chi² = 24.38, df = 1 (P < 0.00001); I² = 96%						
Test for overall effect: Z = 0.67 (P = 0.51)						
**15.1.3 Trial with partial support or author affiliation with pharmaceutical with no sponsor information**						
Dinleyici 2015a	29	29	31	31	15.7%	1.00 [0.94, 1.07]
Dinleyici 2015b (1)	205	220	116	143	14.9%	1.15 [1.05, 1.25]
**Subtotal (95% CI)**		**249**		**174**	**30.6%**	**1.07 [0.88, 1.30]**
Total events	234		147			
Heterogeneity: Tau² = 0.02; Chi² = 12.81, df = 1 (P = 0.0003); I² = 92%						
Test for overall effect: Z = 0.69 (P = 0.49)						
**15.1.4 Trial supported by University/Research council/Foundations**						
Correa 2011	66	95	40	91	7.5%	1.58 [1.21, 2.07]
Hong 2018	140	150	139	150	15.8%	1.01 [0.95, 1.07]
Lai 2019 (2)	39	42	33	39	11.7%	1.10 [0.94, 1.29]
Ritchie 2010	25	33	24	31	7.4%	0.98 [0.75, 1.28]
**Subtotal (95% CI)**		**320**		**311**	**42.4%**	**1.13 [0.92, 1.38]**
Total events	270		236			
Heterogeneity: Tau² = 0.03; Chi² = 17.80, df = 3 (P = 0.0005); I² = 83%						
Test for overall effect: Z = 1.14 (P = 0.26)						
**Total (95% CI)**		**853**		**771**	**100.0%**	**1.11 [1.00, 1.22]**
Total events	779		645			
Heterogeneity: Tau² = 0.01; Chi² = 70.99, df = 7 (P < 0.00001); I² = 90%						
Test for overall effect: Z = 1.99 (P = 0.05)						
Test for subgroup differences: Chi² = 0.20, df = 2 (P = 0.90), I² = 0%						

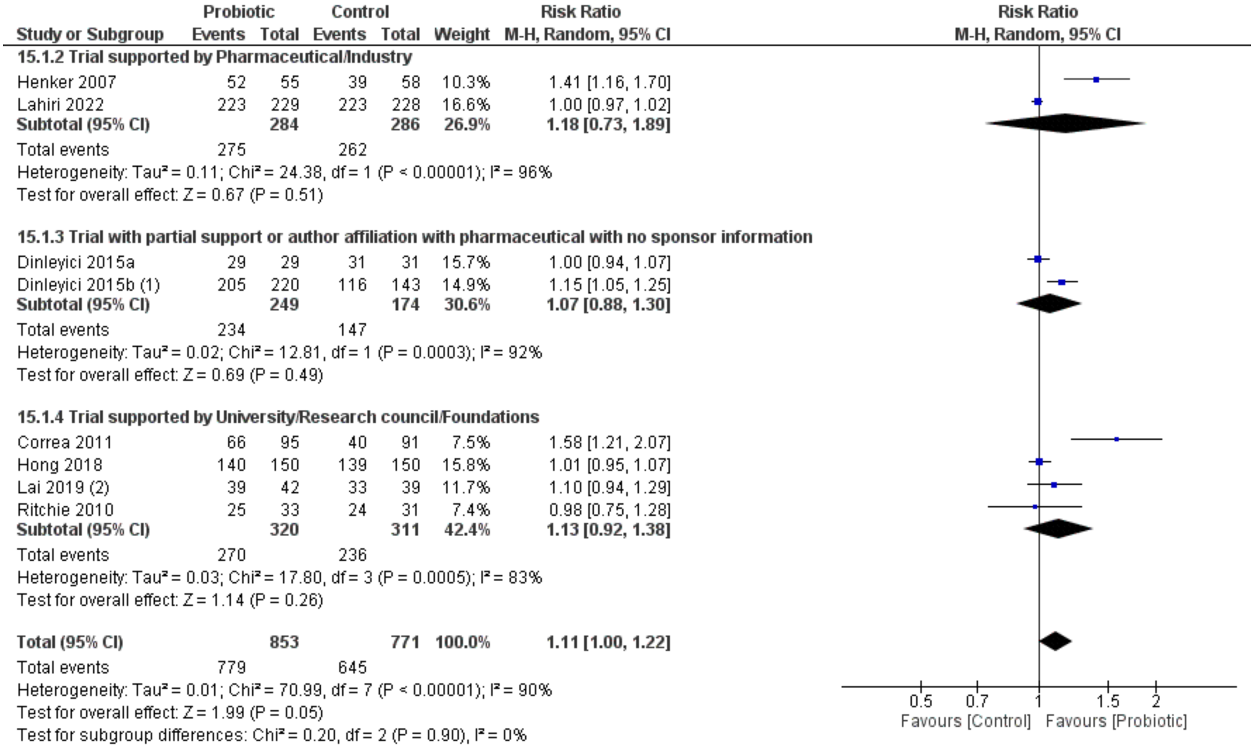

Footnotes
(1) Showed cumulative numbers for children from ED, hospitalized and outpatient settings.
(2) Observed on Day 14

Figure S35

Comparison 4: Source of funding, Outcome 2: Clinical deterioration (WHO-defined studies; n=9)

Study or Subgroup	Probiotic Events	Probiotic Total	Control Events	Control Total	Weight	Risk Ratio M-H, Random, 95% CI
**15.4.2 Trial supported by Pharmaceutical/Industry**						
Freedman 2015	1	61	0	62	0.9%	3.05 [0.13, 73.40]
Lahiri 2022	1	227	2	227	1.5%	0.50 [0.05, 5.48]
Mourey 2020	3	49	8	51	5.4%	0.39 [0.11, 1.39]
Nixon 2012	2	67	1	71	1.5%	2.12 [0.20, 22.83]
Salazar-Lindo 2004	19	90	16	89	24.6%	1.17 [0.65, 2.13]
**Subtotal (95% CI)**		**494**		**500**	**33.9%**	**1.00 [0.60, 1.66]**
Total events	26		27			
Heterogeneity: Tau² = 0.00; Chi² = 3.58, df = 4 (P = 0.47); I² = 0%						
Test for overall effect: Z = 0.01 (P = 0.99)						
**15.4.3 Trial with partial support or author affiliation with pharmaceutical with no sponsor information**						
Freedman 2018	33	414	22	413	32.1%	1.50 [0.89, 2.52]
**Subtotal (95% CI)**		**414**		**413**	**32.1%**	**1.50 [0.89, 2.52]**
Total events	33		22			
Heterogeneity: Not applicable						
Test for overall effect: Z = 1.51 (P = 0.13)						
**15.4.4 Trial supported by University/Research council/Foundations**						
Canani 2007	15	479	4	92	7.5%	0.72 [0.24, 2.12]
Costa-Ribeiro 2003	8	61	6	63	8.8%	1.38 [0.51, 3.74]
Schnadower 2018	15	468	15	475	17.6%	1.01 [0.50, 2.05]
**Subtotal (95% CI)**		**1008**		**630**	**33.9%**	**1.02 [0.61, 1.69]**
Total events	38		25			
Heterogeneity: Tau² = 0.00; Chi² = 0.75, df = 2 (P = 0.69); I² = 0%						
Test for overall effect: Z = 0.07 (P = 0.94)						
**Total (95% CI)**		**1916**		**1543**	**100.0%**	**1.14 [0.85, 1.54]**
Total events	97		74			
Heterogeneity: Tau² = 0.00; Chi² = 5.82, df = 8 (P = 0.67); I² = 0%						
Test for overall effect: Z = 0.89 (P = 0.37)						
Test for subgroup differences: Chi² = 1.50, df = 2 (P = 0.47), I² = 0%						

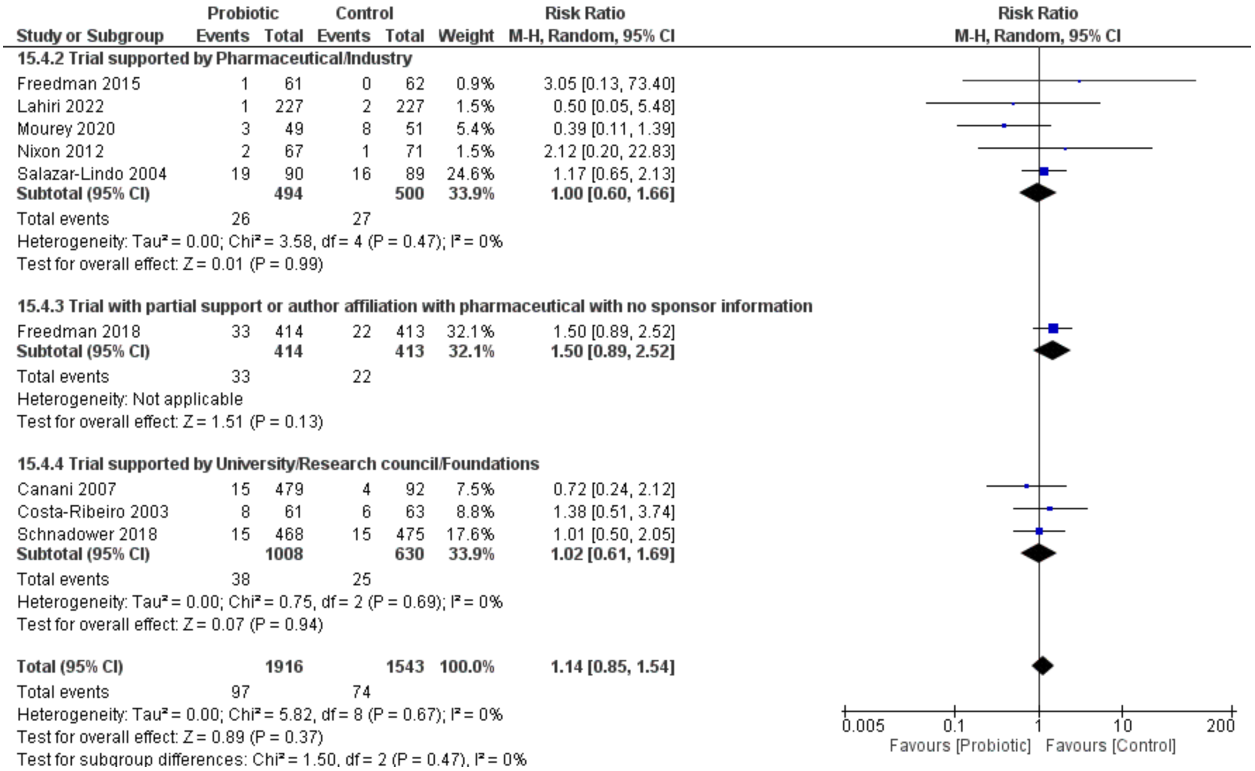

Figure S36

Comparison 4: Source of funding, Outcome 3: Duration of diarrhea (WHO-defined studies; n=16)

Study or Subgroup	Probiotic Mean [Hours]	Probiotic SD [Hours]	Probiotic Total	Control Mean [Hours]	Control SD [Hours]	Control Total	Weight	Mean Difference IV, Random, 95% CI
**15.6.2 Trial supported by Pharmaceutical/Industry**								
Dhongade 2022	79.2	12.96	16	105.6	15.43	16	5.7%	-26.40 [-36.27, -16.53]
Freedman 2015	71.1	78.31	61	63.5	64.3	62	3.0%	7.60 [-17.74, 32.94]
Phavichitr 2013	103.92	58.896	53	120	36.576	53	4.0%	-16.08 [-34.74, 2.58]
Salazar-Lindo 2004	58.5	30.2	52	50.4	28	51	5.4%	8.10 [-3.14, 19.34]
Salazar-Lindo 2007	24.233	39.101	37	24.66	33.21	40	4.5%	-0.43 [-16.70, 15.84]
**Subtotal (95% CI)**			**219**			**222**	**22.5%**	**-6.19 [-22.46, 10.08]**
Heterogeneity: Tau² = 274.14; Chi² = 24.08, df = 4 (P < 0.0001); I² = 83%								
Test for overall effect: Z = 0.75 (P = 0.46)								
**15.6.3 Trial with partial support or author affiliation with pharmaceutical with no sponsor information**								
Dinleyici 2015a	60.4	24.5	29	74.3	15.3	31	5.6%	-13.90 [-24.32, -3.48]
Dinleyici 2015b (1)	58.9	15.3	47	88	19.1	45	6.1%	-29.10 [-36.19, -22.01]
Dinleyici 2015b (2)	83.1	36.3	148	114.8	35.4	72	5.6%	-31.70 [-41.75, -21.65]
Dinleyici 2015b (3)	60.4	23.1	25	78.4	20.9	26	5.2%	-18.00 [-30.11, -5.89]
Freedman 2018	55.533	57.654	414	59.33	61.07	413	6.0%	-3.80 [-11.89, 4.30]
**Subtotal (95% CI)**			**663**			**587**	**28.5%**	**-19.32 [-30.36, -8.28]**
Heterogeneity: Tau² = 134.57; Chi² = 28.32, df = 4 (P < 0.0001); I² = 86%								
Test for overall effect: Z = 3.43 (P = 0.0006)								
**15.6.4 Trial supported by University/Research council/Foundations**								
Aggarwal 2014	62	13.54	100	80	13.539	100	6.5%	-18.00 [-21.75, -14.25]
Canani 2007	96.3122	36.8789	479	112.56	23.95	92	6.3%	-16.25 [-22.15, -10.34]
Chen 2010	60.1	31.7	150	86.3	37.6	143	6.0%	-26.20 [-34.18, -18.22]
Costa-Ribeiro 2003	38.27	3.78	61	39.09	4.6	63	6.7%	-0.82 [-2.30, 0.66]
Hong 2018	80.33	42.667	150	79.66	41.17	150	5.7%	0.67 [-8.82, 10.16]
Huang 2014	43.2	38.4	82	69.4	33.6	77	5.4%	-26.20 [-37.40, -15.00]
Misra 2009	70.56	31.2	105	78	26.4	105	6.0%	-7.44 [-15.26, 0.38]
Schnadower 2018	51.633	50.268	468	54.7	46.99	475	6.3%	-3.07 [-9.28, 3.15]
**Subtotal (95% CI)**			**1595**			**1205**	**48.9%**	**-11.90 [-19.67, -4.14]**
Heterogeneity: Tau² = 112.35; Chi² = 133.34, df = 7 (P < 0.00001); I² = 95%								
Test for overall effect: Z = 3.00 (P = 0.003)								
**Total (95% CI)**			**2477**			**2014**	**100.0%**	**-12.85 [-18.76, -6.94]**
Heterogeneity: Tau² = 135.19; Chi² = 235.15, df = 17 (P < 0.00001); I² = 93%								
Test for overall effect: Z = 4.26 (P < 0.0001)								
Test for subgroup differences: Chi² = 2.00, df = 2 (P = 0.37), I² = 0%								

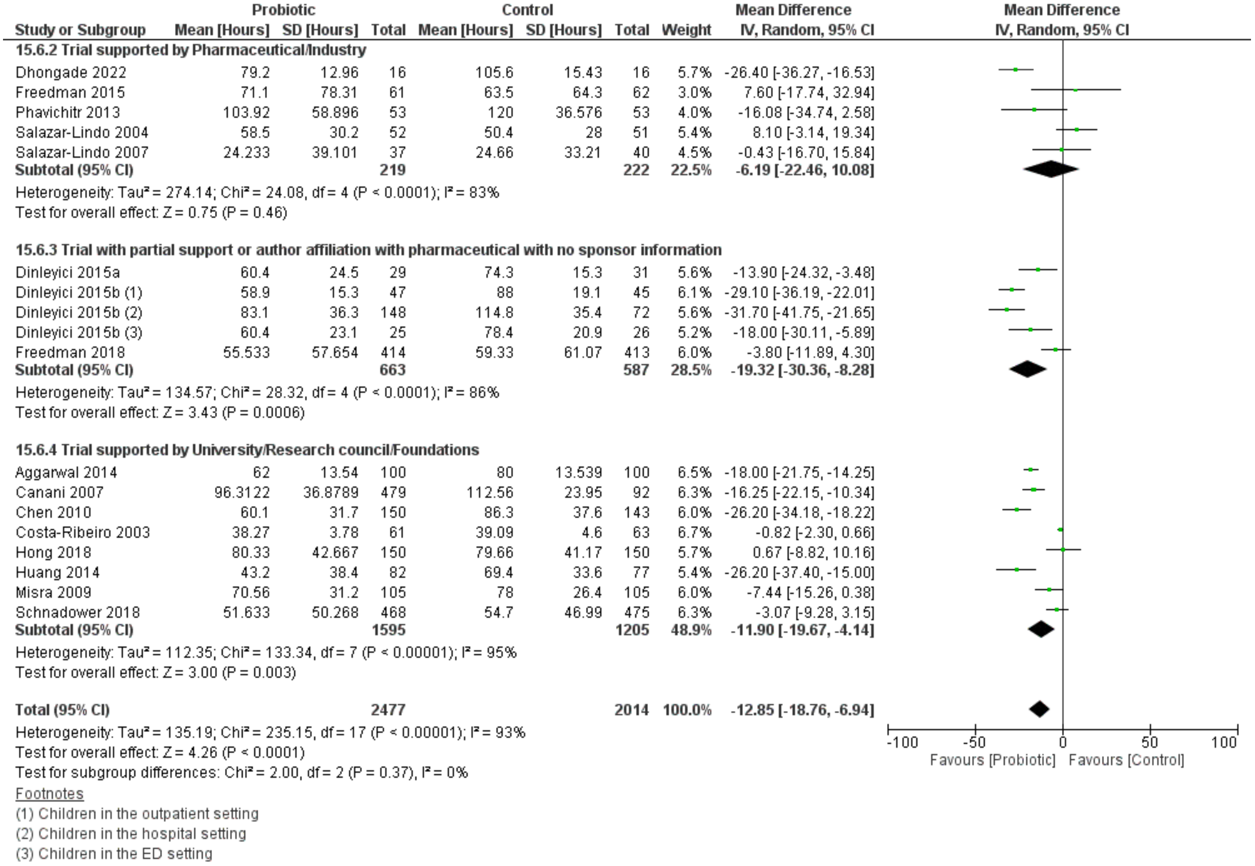

Footnotes
(1) Children in the outpatient setting
(2) Children in the hospital setting
(3) Children in the ED setting

Figure S37

Comparison 4: Source of funding, Outcome 4: Mortality (n=3)

Study or Subgroup	Probiotic Events	Probiotic Total	Control Events	Control Total	Weight	Risk Ratio M-H, Random, 95% CI
**15.5.2 Trial supported by Pharmaceutical/Industry**						
Lahiri 2022	0	227	0	227		Not estimable
**Subtotal (95% CI)**		**227**		**227**		**Not estimable**
Total events	0		0			
Heterogeneity: Not applicable						
Test for overall effect: Not applicable						
**15.5.3 Trial with partial support or author affiliation with pharmaceutical with no sponsor information**						
Pernica 2022 (1)	1	135	6	137	100.0%	0.17 [0.02, 1.39]
**Subtotal (95% CI)**		**135**		**137**	**100.0%**	**0.17 [0.02, 1.39]**
Total events	1		6			
Heterogeneity: Not applicable						
Test for overall effect: Z = 1.66 (P = 0.10)						
**15.5.4 Trial supported by University/Research council/Foundations**						
Pernica 2017 (2)	0	15	0	15		Not estimable
Pernica 2017 (3)	0	12	0	19		Not estimable
**Subtotal (95% CI)**		**27**		**34**		**Not estimable**
Total events	0		0			
Heterogeneity: Not applicable						
Test for overall effect: Not applicable						
**Total (95% CI)**		**389**		**398**	**100.0%**	**0.17 [0.02, 1.39]**
Total events	1		6			
Heterogeneity: Not applicable						
Test for overall effect: Z = 1.66 (P = 0.10)						
Test for subgroup differences: Not applicable						

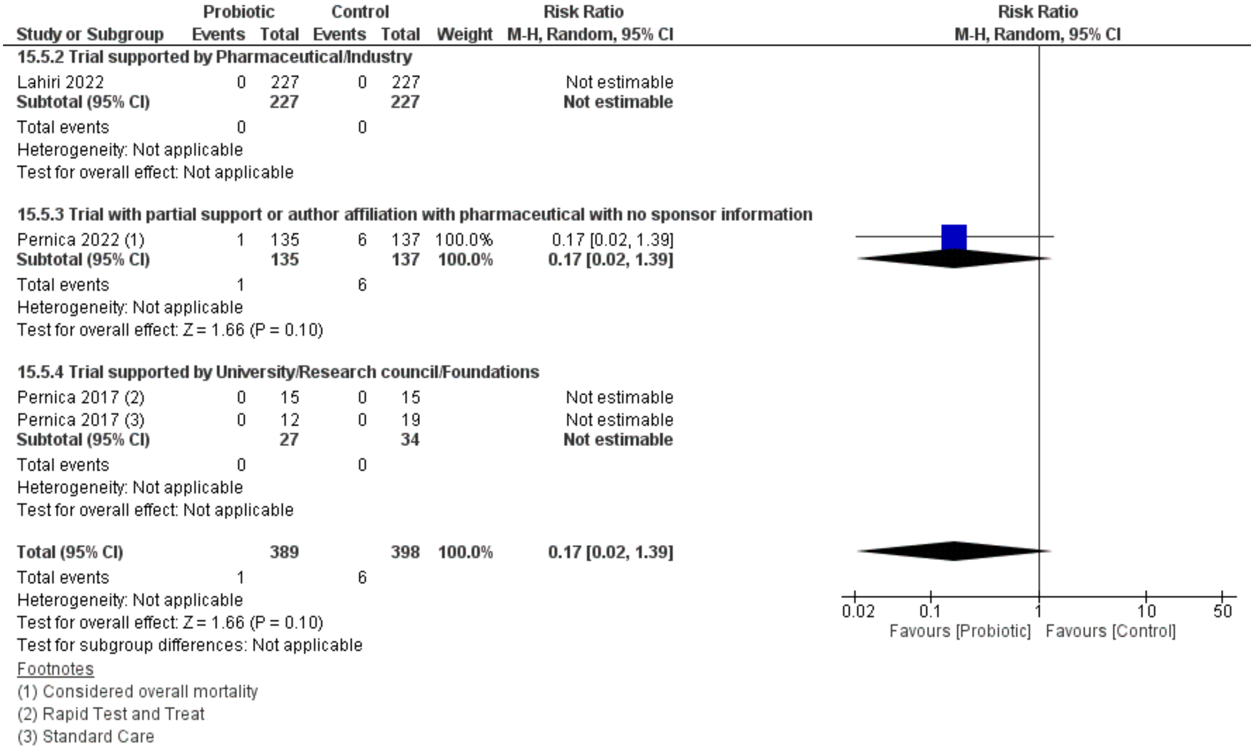

Footnotes
(1) Considered overall mortality
(2) Rapid Test and Treat
(3) Standard Care
